# The *rolB*‐transgenic *Nicotiana tabacum* plants exhibit upregulated *ARF7* and *ARF19* gene expression

**DOI:** 10.1002/pld3.414

**Published:** 2022-06-18

**Authors:** Rahul Bose, Mainak Sengupta, Debabrata Basu, Sumita Jha

**Affiliations:** ^1^ Department of Genetics University of Calcutta Kolkata West Bengal India; ^2^ Division of Plant Biology Bose Institute Kolkata West Bengal India; ^3^ Department of Botany University of Calcutta Kolkata West Bengal India

**Keywords:** *ARF*s, auxin, gene ontology, gene‐upregulation, hairy root, *rolB*

## Abstract

*Agrobacterium rhizogenes* root oncogenic locus B (*rolB*) is known to induce hairy roots along with triggering several physiological and morphological changes when present as a transgene. However, it is still unknown how this gene triggers these changes within the plant system. In this study, the effect of *rolB in‐planta*, when present as a transgene, was assessed on the gene expression levels of auxin response factors (*ARF*s)—transcription factors which are key players in auxin‐mediated responses. The goal was to uncover Auxin/*ARF*‐driven transcriptional networks potentially active and working selectively, if any, in *rolB* transgenic background, which might potentially be associated with hairy root development. Hence, the approach involved establishing *rolB*‐transgenic *Nicotiana tabacum* plants, selecting *ARF*s (Nt*ARF*s) for context‐relevance using bioinformatics followed by gene expression profiling. It was observed that out of the chosen Nt*ARF*s, Nt*ARF7* and Nt*ARF19* exhibited a consistent pattern of gene upregulation across organ types. In order to understand the significance of these selective gene upregulation, ontology‐based transcriptional network maps of the differentially and nondifferentially expressed *ARF*s were constructed, guided by co‐expression databases. The network maps suggested that Nt*ARF7*‐Nt*ARF19* might have major deterministic, underappreciated roles to play in root development in a *rolB*‐transgenic background—as observed by higher number of “root‐related” biological processes present as nodes compared to network maps for similarly constructed other non‐differentially expressed *ARF*s. Based on the inferences drawn, it is hypothesized that *rolB*, when present as a transgene, might drive hairy root development by selective induction of Nt*ARF7* and Nt*ARF19*, suggesting a functional link between the two, leading to the specialized and characteristic *rolB*‐associated traits.

## Significance statement

In this study, it was observed that Nt*ARF7* and Nt*ARF19* are upregulated in *rolB*‐transgenic *Nicotiana tabacum* plants. To understand their potential relevance, Gene Ontology‐based transcriptional network maps for differentially and nondifferentially expressed *ARF*s were constructed. Higher number of “root‐related” biological processes were observed for differentially upregulated *ARF*s as compared with nondifferentially expressed *ARF*s. Based on the inferences, the existence of a functional link was postulated between *rolB* and auxin via Nt*ARF7*‐Nt*ARF19* leading to the characteristic *rolB*‐associated hairy root development in *rolB*‐transgenic background.

## 1 INTRODUCTION

*Agrobacterium rhizogenes* root oncogenic locus B (*rolB*) along with other *rol* genes, *rolA*, *rolC*, and *rolD*, is encoded by the root‐inducing (Ri) Transfer‐DNA (T‐DNA) of mannopine and T_L_‐DNA (Transfer_Left_‐DNA) of agropine type plasmid strains of *A. rhizogenes* (Bahramnejad et al., 2019; Slightom et al., 1986; White et al., 1985). In cucumopine type plasmid strains of *A. rhizogenes* such as K599/NCPPB2659, *rolB* is present with *rolA* and *rolC* followed by *orf13* on its T‐DNA (Filetici et al., 1987; Serino et al., 1994). Genetic transformation of host plants with these oncogenic strains of *A. rhizogenes* give rise to T‐DNA transformed roots which have characteristic traits such as rapid plagiotropic growth with increased branching and phytohormone independent growth (Tepfer, 1984, 1990). These traits are attributed primarily to the presence of *rol* genes. Moreover, spontaneous or induced regeneration of T‐DNA transformed plants from T‐DNA transformed roots is also facilitated by these *rol* genes (Tempé et al., 1984) in a number of species (Desmet et al., 2020; Sarkar & Jha, 2021; Sarkar et al., 2018). Functional studies have previously demonstrated the individual effects of genes, for example, constitutive expression of *rolA* leads to bushy short *Nicotiana tabacum* plants with compact inflorescences accompanied with reduction in phytohormone content (Dehio et al., 1993) and increased resistance toward the pathogen *Fusarium oxysporum* in tomato (Bettini et al., 2016). RolC was demonstrated to hydrolyze certain cytokinin‐conjugates liberating cytokinin determined in vitro and in soluble extracts from *N. tabacum* plants expressing it as a transgene (Estruch, Chriqui, et al., 1991), but literature is not yet conclusive on the cytokinin glucosidase functioning of RolC (Faiss et al., 1996)*. rolD* encodes for a protein which exhibits ornithine cyclodeaminase activity in vitro and in soluble extracts from *rolD* transgenic *N. tabacum* plants (Trovato et al., 2001). Interestingly, although the four *rol* genes exhibit synergism in hairy root development (Oono et al., 1990; Schmülling et al., 1988, 1993), transgenic expression of *rolB* leads to the induction of hairy roots (White et al., 1985), a hallmark phenotype exhibited by host plants when infected by oncogenic *A. rhizogenes* (Aoki & Syono, 1999; Capone et al., 1989; Cardarelli, Mariotti, et al., 1987).

The development of hairy roots induced by *rolB* is of special interest. These hairy roots exhibit altered metabolic pathways and can serve as a source of diverse secondary metabolites with biotechnological and pharmaceutical relevance (Halder et al., 2019; Mishra & Ranjan, 2008). Studies have been conducted previously to understand the root inducing potential of *rolB* in plants. Though these studies were not conclusive enough, few initial landmark studies did establish a functional link of *rolB* with auxin by demonstrating that *rolB*‐transgenic plants exhibit increased sensitivity to auxin (Delbarre et al., 1994; Schmülling et al., 1993). In *rolB*‐transformed shoots of apple rootstock M26, Jork9 and pear rootstock BP10030 (*Pyrus communis*), enhanced rooting and increased roots per shoot in the absence of auxin were observed, suggesting that endogenous auxin levels are sufficient to induce spontaneous rooting when *rolB* is present as transgene (Sedira et al., 2001; Welander et al., 1998; Zhu et al., 2003). Despite establishing a functional link between an auxin response and *rolB*, the molecular mechanisms and functions of *rolB* during hairy root development is still unknown. As the formation of roots requires the establishment of a local auxin maxima at the initiating cells triggering the formation of a root primordia (Dubrovsky et al., 2008; Su et al., 2011), it can be suggested that RolB by acting as a glucosidase (Estruch, Schell, & Spena, 1991) provides the required auxin maxima by converting inactive auxin‐conjugates to active auxin. However, these findings were refuted in subsequent studies, as no change in intracellular auxin concentration was detected (Nilsson et al., 1993). It was later suggested that RolB acts as a tyrosine phosphatase (Filippini et al., 1996) and possibly perturbs the auxin sensitivity of the plant by post‐translational modification of endogenous auxin signaling pathways, thereby bypassing the need to modulate endogenous auxin levels. Filippini et al. (1996) also stated that RolB is localized in the plasma membrane whilst the work of Moriuchi et al. (2004) demonstrated activity of RolB within the nucleus by binding with *Nt*14‐3‐3‐like ωII—a molecular adaptor protein. Both studies are contradictory in regard to the location where RolB is active. Moriuchi et al. (2004), however, did not explain how this intranuclear association between *rolB* and *Nt*14‐3‐3‐like ωII influences the hairy root specific‐auxin signaling pathway when *rolB* is present as transgene. Therefore we can conclude that at the moment, the exact molecular function of RolB during hairy root development is not fully known.

This knowledge gap in understanding how RolB triggers hairy root formation necessitates an in‐depth study at different levels including gene expression. Root development is a very complex developmental process driven by the coordinated expression of several genes. In this context, auxin and root‐development specific transcription factors (TFs), auxin response factors (*ARFs*), are known as major initiation factors for root development (Gutierrez et al., 2009; Mayer et al., 1991; Okushima et al., 2007). A study investigating the expressional status of these *ARF*s in *rolB*‐transgenic plants is promising as it has the potential to answer outstanding questions concerning the root inducing properties of *rolB* in hairy root development and whether a functional link exists with auxin via *ARF*s—by identification of the *ARF*s which are under the oncogenic influence of *rolB*. Additionally, this also has the potential to reveal/identify which *ARF*‐driven transcriptional networks are selectively active, if any, providing clues to the mechanism by which specific traits induced by transgenic expression of *rolB*.

In the present study, the functional link between *rolB* and Auxin via *ARF*s was investigated by studying the expression status of *ARF*s in *rolB*‐transgenic plants, in order to identify differentially expressed *ARF*s. Gene‐ontology based transcriptomic networks guided by co‐expression databases, comprising of genes which are putative downstream target genes of these differentially upregulated *ARF*s, were also proposed in order to comprehend how these upregulated *ARF*s might drive or activate the root development related functions in *rolB‐*transgenic plants.

## 2 EXPERIMENTAL PROCEDURES

### 2.1 Bacterial strains and growth conditions

*Escherichia coli* DH5α (Hanahan, 1983), *A. rhizogenes* wild‐type strain A4 (Cardarelli et al., 1985) and *Agrobacterium tumefaciens* LBA4404 (Ooms et al., 1982) were used in this study. *E. coli* was used for cloning applications and routinely grown in Luria Broth (LB, HiMedia) medium (Bertani, 1951) at 37°C with overnight incubation in a gyratory shaker at 180 rpm. The LB medium consisted of (gms l^‐l^): tryptone—10.0; NaCl—10.0; yeast extract (Difco)—5.0; pH 7. *A. rhizogenes* strain A4 was used for cloning of *rolB*_TL_ and grown in liquid Yeast Mannitol Broth (YMB, HiMedia) medium (Hooykaas et al., 1977) incubated at 28°C for 24–48 h at 180 rpm. The YMB medium was composed of (gms L^−l^) mannitol—10.0; yeast extract (Difco)—0.4; NaCl—0.1; MgSO_4_.7H_2_O—0.2; K_2_HPO_4_—0.5; pH 6.8. *A. tumefaciens* LBA4404 was used for plant genetic transformations, and grown in LB medium supplemented with 50 mg L^−1^ rifampicin in dark at 28°C for 24–48 h at 180 rpm.

### 2.2 Plant materials and growth conditions

Seeds of *N. tabacum* var SR1 were surface sterilized (Fisher & Guiltinan, 1995) and cultured on half strength solid MS medium (Murashige & Skoog, 1962) with 1.5% sucrose(w/v). After 3 weeks, the germinated seedlings were transferred to 250‐ml culture flasks containing 50 ml of half strength solid MS media (HiMedia, India) with 1.5% (w/v) sucrose. The nodal explants from 6–8 weeks old axenic plants were used for vegetative propagation in vitro. The shoot cultures were maintained in half strength solid MS media with 1.5% (w/v) sucrose at 26°‐28° C under a 16/8 hrs (light/dark) photoperiod with light supplied by cool white fluorescent lamps (Philips, India) at an intensity of 48 μmol m^−2^ s^−1^.

### 2.3 Cloning of *rolB* and construction of *rolB*‐recombinant plant transformation vector

The nucleotide sequence of *rolB* comprising of 780 bp protein coding ORF and 1239 bp upstream of ATG was retrieved from NCBI database (Accession No: K03313.1). For sequence verification, the ORF sequence was subjected to *in‐silico* translation and subsequent BLASTx analysis. This sequence corresponded to *rolB* within T_L_‐DNA fragment of *A. rhizogenes* A4 Ri plasmid. Primers (Table S1) with appropriate restriction enzyme sites (*Sma*I, *Sal*I) were designed using NetPrimer (http://www.premierbiosoft.com/citations/netprimer.html ) online tool and delivered by IDT (Integrated DNA Technologies). The primers encompassed an amplicon length of 2019 bp, comprising of 780 bp *rolB* ORF and 1239 bp upstream of ATG (of *rolB* ORF). The amplicon was PCR amplified from total DNA obtained from *A. rhizogenes* A4, purified using QIAquick PCR Purification kit (QIAGEN) and digested with restriction enzymes (*SmaI*, *SalI*, Roche) for subsequent ligation (T4 DNA Ligase, Thermo Scientific) into pBluscript II‐SK+. Competent *E. coli* DH5α cells were transformed with the ligation mixture using heat shock method (Bergmans et al., 1980). The competent cells of *E. coli* DH5α were prepared following the method of Green and Rogers (2013), with an addition of 10 mM (3‐(N‐Morpholino)‐propanesulfonic acid [MOPS]) to the transformation buffer‐I. Positive transformants were selected on LB‐ampicillin (100 mg L^−1^) medium, screened visually using Blue‐White screening and confirmed by colony PCR using *rolB* specific primers. Recombinant vectors were isolated from these positive transformants and confirmed for cloning by digestion with restriction enzymes. Sequence integrity of the cloned *rolB* was verified by DNA Sequencing (BigDye Terminator v3.1 Cycle Sequencing Kit). The cloned *rolB* was excised from *rolB*‐pBluescript II SK+ recombinant vector by restriction enzyme digestion (*Sma*I, *Sal*I; *Roche)* and introduced in a modified pCAMBIA1301 (MpCAMBIA1301) (Mukherjee et al., 2019) plant transformation vector by ligation. Subsequent transformation into competent *E. coli* DH5α cells using heat shock method (Bergmans et al., 1980) was performed. Positive recombinants were selected by growth on LB‐Kanamycin (50 mg L^−1^) plates and screened by colony PCR using *rolB* specific primers. The recombinant *rolB*‐MpCAMBIA1301 vector was then isolated from positive recombinants and confirmed for presence of *rolB* using restriction enzyme digestion. Subsequently, the recombinant *rolB*‐MpCAMBIA1301 and non‐recombinant MpCAMBIA1301 vectors were introduced into competent *A. tumefaciens* LBA4404 (Dityatkin et al., 1972) using freeze–thaw method (Holsters et al., 1978). The *A. tumefaciens* LBA4404 strains bearing recombinant *rolB*‐MpCAMBIA1301 and non‐recombinant MpCAMBIA1301 were used for plant genetic transformation experiments.

### 2.4 Genetic transformation of *N. tabacum* with vector‐bearing *A. tumefaciens* LBA4404 strains

Axenic *N. tabacum* plants were used for plant transformation with *A. tumefaciens* LBA4404 harboring the recombinant vector, *rolB*‐MpCAMBIA1301, and *A. tumefaciens* LBA4404 harboring the non‐recombinant vector, MpCAMBIA1301, by leaf disc method (Gallois & Marinho, 1995; Horsch et al., 1985). Briefly, *A. tumefaciens* LBA4404 bearing *rolB*‐MpCAMBIA1301 and MpCAMBIA1301 were grown for 48 h in LB medium supplemented with rifampicin (50 mg L^−1^) and kanamycin (50 mg L^−1^); 25 μl of this culture was used to inoculate 50 ml of LB medium supplemented with Kanamycin (50 mg L^−1^) and grown overnight. The bacterial culture was centrifuged at 2800 g for 5 mins and the cell pellet was resuspended in liquid MS media to obtain a bacterial suspension with O.D. of 0.6–0.8. The leaf discs (1 cm × 1 cm) were then co‐cultivated with *A. tumefaciens* LBA4404 bearing *rolB*‐MpCAMBIA1301 and MpCAMBIA1301 in suspension for 10 min. For control, leaf discs were similarly co‐cultivated in uninoculated MS medium. The explants were then incubated on MS + 2 mg L^−1^ BAP at 26°–28° C in dark for 48 h. The leaf discs were then washed in sterile distilled water followed by thorough washing in cefotaxime (1000 mg L^−1^), blotted dry and then cultured on MS medium supplemented with 2 mg L^−1^ BAP, 500 mg L^−1^ cefotaxime, with or without 25 mg L^−1^ hygromycin B (regeneration media, Fisher & Guiltinan.,1995) for 8 weeks. The putative transgenic calli were subcultured on regeneration media containing 500 mg L^−1^ cefotaxime and 25 mg L^−1^ hygromycin B every 4 weeks till the emergence of shoot buds. The microshoots (>1 cm in length) were excised from the regenerating calli and transferred to MS media without phytohormones but with 500 mg L^−1^ cefotaxime and 25 mg L^−1^ hygromycin B for further growth into rooted plantlets (T_0_).

### 2.5 Selection and maintenance of transgenic *N. tabacum* plants

The transgenic plants with well‐developed shoot and root were maintained in MS + 25 mg L^−1^ Hygromycin B + 500 mg L^−1^ Cefotaxime with four weekly subculture for 8 weeks. These T_0_ plants were then transferred to pots containing soilrite mix comprising of horticulture grade perlite, peat moss and compost (Garden Blossom, New Delhi) and maintained in greenhouse (28° C). After 1 month, the T_0_ plants were transferred to pots containing soil. After 4–5 months of transfer to soil, seeds were collected from these T_0_ plants. The seeds (as T_1_) from each T_0_ transgenic plant line were surface sterilized and cultured on petriplates containing 10 ml of MS medium with or without 25 mg L^−1^ Hygromycin B. As control, seeds from wildtype plants (untransformed) were similarly surface sterilized and cultured on petriplates containing 10 ml of MS medium with or without 25 mg L^−1^ Hygromycin B. Data on seed germination was determined after 4 weeks of culture for each plant line. The T_1_ transgenic hygromycin B‐resistant seedlings (4 weeks old) were transferred to 250 ml culture flasks containing 50 ml of MS medium with 25 mg L^−1^ Hygromycin B and maintained for 8 weeks prior to transfer to greenhouse (28°C). The seeds (as T_2_) from the transgenic T_1_ plant lines were plated on petriplates containing 10 ml of MS medium with or without 25 mg L^−1^ Hygromycin B for germination and used for further studies.

### 2.6 Confirmation of *rolB* transgene insertion and expression

- **GUS staining:** GUS staining (Jefferson, 1987) was performed using leaves of 4 week old vector control (T_0_) plants and *rolB*‐transgenic (T_0_) plants. GUS staining was also performed in seedlings of vector control (T_1_, T_2_) and *rolB*‐transgenic (T_1_, T_2_) generations. Briefly, leaves and seedlings were dipped in GUS staining solution (GSS) and vacuum infiltrated two times for 15 min accompanied with gentle tapping. The samples were then incubated overnight at 37°C. The residual GSS was drained and the samples were fixed in FAA solution for 30 min. This was followed by repeated washing with 70% ethanol for de‐chlorophyllization resulting in clear visibility of the resulting GUS stain.
- **PCR:** Genomic DNA was extracted from 4 weeks old vector control (T_0_, T_1_, T_2_) and *rolB*‐transgenic (T_0_, T_1_, T_2_) plant lines using DNeasy Plant Mini‐Prep kit (QIAGEN) according to manufacturer's instructions. Extracted DNA was analyzed by PCR for *rolB* (Christensen et al., 2008; Sevón et al., 1997) and hygromycin phosphotransferase (*hptII*—AF234297.1, AAF65341.1) genes using gene‐specific primers (Table S1). Genomic DNA extracted from untransformed plant was used as negative control. Amplicons for *rolB* CDS (780 bp) and *hptII* (453 bp) were resolved by gel electrophoresis in 1.2% agarose gel and compared against GeneRuler 1 kb Plus DNA ladder (Thermo Fisher Scientific) as reference. Visualization was done by EtBr staining under UV light and documented using Gel Doc Imaging system (Bio‐Rad).
- **RNA isolation, cDNA synthesis and Reverse‐Transcriptase (RT) PCR:** Expression of *rolB* transgene was confirmed by Reverse‐Transcriptase PCR (RT‐PCR). RNA was isolated from leaf, stem and root samples of 4 weeks old *rolB*‐transgenic (T_0_,T_1_,T_2_) plant lines and vector control (T_0_,T_1_,T_2_) plants. RNA isolation was done by NucleoSpin RNA isolation kit (Macherey‐Nagel) according to manufacturer's instructions. Genomic DNA was eliminated by RNAse free DNAse (RQ1, Promega Corporation) treatment. RNA concentration was determined spectrophotometrically. 2 μg of RNA was used to synthesis cDNA using SUPERSCRIPT cDNA synthesis kit (Invitrogen, ThermoFisher Scientific) following manufacturer's instructions. The cDNA was diluted 10 times and genomic DNA contamination of RNA samples was ruled out by performing PCR with diluted cDNA samples using Quinolinate PhosphoribosylTransferase 2 (*QPT2*) gene specific intron flanking primers (Table S1). *rolB* transgene expression was confirmed by RT‐PCR using diluted cDNA as template with *rolB* and *L25* gene (60S ribosomal protein L25; internal reference gene; Schmidt & Delaney, 2010) specific primers. The Reverse Transcriptase PCR (RT‐PCR) was performed at 25, 28, 30, and 35 cycles and was found to reach saturation at 35 cycles. The PCR products were run in 2% agarose gels and was visualized in a Gel Doc Imaging system (Bio‐Rad).

### 2.7 Identification and retrieval of context‐specific *ARF*s via datamining of gene ontology database in silico

*ARFs* involved in root development were identified by data mining of Gene ontology database file (ATH_GO_GOSLIM.txt, updated version—2020‐02‐01) obtained from TAIR website (https://www.arabidopsis.org) (Berardini et al., 2015). AWK/Python scripts were written to separately identify TFs (keyword “transcription factor”) and involved in any aspect of root development (keyword “root”)—this was our context of relevance. Records that were found common between these lists were filtered and listed separately. Functional categorization of these records into protein groups was done by retrieving their corresponding protein sequences, performing a multiple sequence alignment by MAFFT using default parameters (Katoh et al., 2002) followed by a simple clustering analysis (ClustalX, algorithm Neighbour Joining; Saitou & Nei, 1987, Bootstrap—10000). *ARF*s were subsequently retrieved from this cluster tree.

### 2.8 Gene expression profiling of *ARF*s in transgenic *N. tabacum* plants by real‐time PCR

Due to availability of partially annotated *N. tabacum* genome (Sol Genomics, https://solgenomics.net/; Fernandez‐Pozo et al., 2015), it was necessary to identify sequences that correctly represented each *ARF*. Hence, full‐length mRNA and CDS sequences (each with a start and stop codon) corresponding to different *ARF*s were retrieved from Sol Genomics Database (*N. tabacum*, *Solanum lycopersicum* [tomato]), TAIR (*Arabidopsis thaliana*), NCBI (*N. tabacum*). These sequences were subjected to multiple sequence alignment (via MAFFT) followed by subsequent clustering (ClustalX, NJ tree, bootstrap—100 replicates); sequences truly representing each *ARF* clustered together and were thus identified. The inclusion of *A. thaliana* (distantly related to tobacco) and *S. lycopersicum* (closely related to tobacco) was done to correctly identify *ARF* sequences in *N. tabacum* through comparisons between the orthologs and paralogs (Table S2). Primers for Realtime PCR were designed targeting 150–200 bp unique regions of each Nt*ARF* from the sequences that exhibited highest similarity scores to *A. thaliana* and *S. lycopersicum* *ARF*s orthologs (Tables S1 and S2). The eight *ARF*s selected were *NtARF5*, *NtARF6*, *NtARF7*, *NtARF8*, *NtARF10*, *NtARF16*, *NtARF17*, *NtARF19*. RNA was isolated from leaf, stem and root samples of 4 weeks old vector control (EV2) and five *rolB*‐transgenic T_1_ plant lines (five biological replicates for each plant line) using NucleoSpin RNA isolation kit (Macherey‐Nagel) according to manufacturer's instructions. Extracted RNA was subjected to RNAse free DNAse (RQ1, Promega Corporation) treatment and concentration was determined spectrophotmetrically. cDNA was synthesized from 2 μg of RNA using SUPERSCRIPT cDNA synthesis kit (Invitrogen, ThermoFisher Scientific) following manufacturer's instructions. Genomic DNA contamination of RNA samples was estimated by performing PCR with diluted cDNA samples using Quinolinate PhosphoribosylTransferase 2 (*QPT2*) gene specific intron flanking primers (Table S1). Real‐time PCR was done using SYBR Green Real‐Time PCR master mix (Thermo Fisher Scientific) on ABI 7500 FastSDS Real‐Time PCR system (with two technical replicates for each RNA sample). For data normalization, *L25* gene (60S ribosomal protein L25) was chosen as the internal reference gene (Schmidt & Delaney, 2010). Transcript levels were evaluated as relative values to vector control EV2 (the value of 1) and Relative fold change (RFC) was calculated using ΔΔCt method (Livak & Schmittgen, 2001).

### 2.9 Construction of gene‐ontology based transcriptomic network maps comprising of downstream targeted genes of Nt*ARF*s

A gene‐ontology transcriptomic network map driven by *ARF*s was constructed, comprising of genes which are the downstream targets of the former. The following strategy was implemented—it was assumed that for a gene to be driven by an *ARF*, it must be (1) co‐expressed spatially and/or temporally with the *ARF* and (2) the upstream elements/promoters of the gene(s) must have *ARF* binding motif sites (AuxRE—auxin response factor binding sites). Based on these assumptions, genes that are co‐expressed with *ARF*s across multiple experiments representing different temporal and spatial conditions were initially mined from CORNET (https://bioinformatics.psb.ugent.be/cornet/versions/cornet2.0/)—a gene co‐expression database (Bodt et al., 2010, 2012) with the following filter parameters—correlation coefficient ≥.7, *p* value ≤.05, and only top 20,000 genes per selected genes were reported. Next, the upstream element sequences (3000 bp) of these genes were retrieved from TAIR database and were scanned for AuxRE motif (*ARF* binding sites) using FIMO (of MEME suite; Bailey et al., 2009; Grant et al., 2011). The AuxRE motifs as input for FIMO were obtained from PlantTFDB (Plant Transcription factor database; Jin et al., 2017) as their position‐weight matrices (PWMs). Genes with atleast one AuxRE in their 3000 bp upstream sequence were then selected from the CORNET retrieved co‐expressed genes. The selected AuxRE‐possessing, *ARF*‐co‐expressing genes were then analyzed for their functional role using BiNGO plugin (Maere et al., 2005) of Cytoscape (Shannon, 2003). An ontology based network map using genes as input was obtained using “biological process” as selection criterion. Such maps were similarly prepared for different *ARF*s and then compared for enrichment of root‐development related “biological process” categories for understanding functional relevance in the context of *rolB*‐mediated hairy root development.

### 2.10 Statistical analysis

Statistical analysis was performed in R statistical programming language (R Core Team, 2020). The segregation of transgene cassette in T_1_ transgenic seeds was assessed by Chi‐Square test for Goodness of Fit to 3:1. Relative fold change (RFC) of Nt*ARF*s expression were tested for statistical significance using non‐parametric Mann–Whitney test (McKnight & Najab, 2010; Neve et al., 2013). The barplots and heatmaps were built in Microsoft Excel using the mean RFCs as data points.

## 3 RESULTS

### 3.1 Cloning of *rolB* into plant binary vector for genetic transformation

**FIGURE 1:**
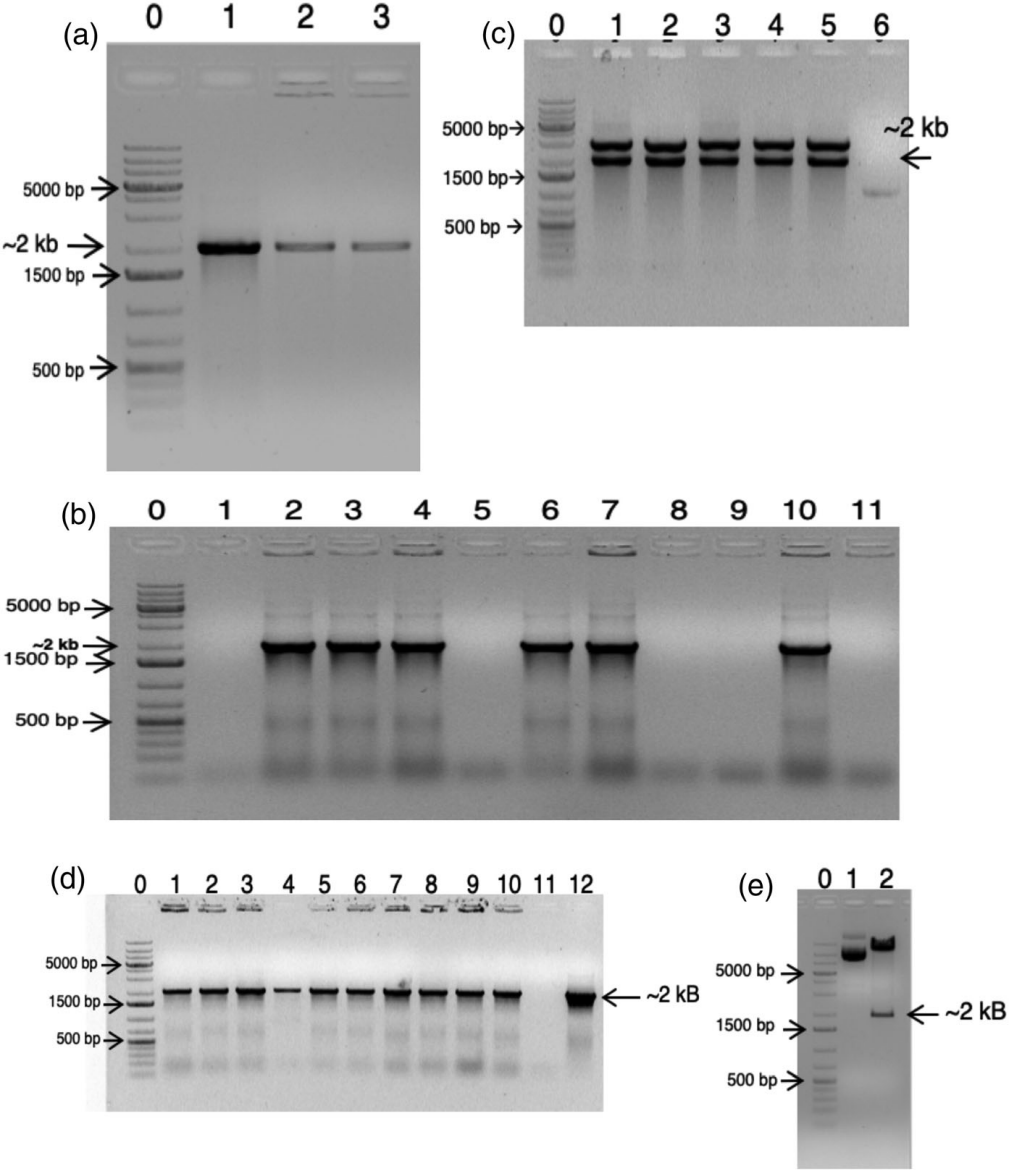
Results of agarose gel electrophoresis—(a) presence of ~2 kb DNA band confirms the amplification of *rolB* from *Agrobacterium rhizogenes* A4; lane 0—1 kb DNA ladder, lanes 1 to 3—*rolB* gene amplification product; (b) Colony PCR based confirmation of *rolB*‐pBluescript II SK + *E. coli* colonies. Presence of a single ~2 kb DNA band confirms positive clones; lane 0—1 kb DNA ladder, lane 1—Negative control, lane 2 to 11—Putative *rolB E. coli* clones. Primers used were specific for *rolB*; (c) restriction enzyme digestion of *rolB*‐pBluescript II SK + vector; lane 6—Positive control. Presence of a pop‐out at ~2 kb confirmed the presence of *rolB* as an insert in the recombinant vector; (d) presence of ~2 kb DNA band confirms the positive *E. coli* DH5α clones bearing *rolB*‐MpCAMBIA1301 (Colony PCR done with *rolB* specific primers). Lane 0—1 kb DNA ladder, lanes 1 to 10‐ putative *rolB‐*MpCAMBIA1301 harboring *E. coli* clones, exhibiting 2 kb DNA band, lane 11—Negative control, lane 12—Positive control; (e) restriction enzyme digestion of vector isolated from DH5α *E. coli* colonies. The presence of popout of size ~2 kb bp confirmed the presence of *rolB*. Lane 0–1 kb DNA ladder, lane 1: MpCAMBIA1301 digestion (negative control), lane 2: *rolB‐*MpCAMBIA1301 digestion

The *rolB* was amplified using gene specific PCR primers and led to the successful amplification visualized as a sharp distinct DNA band of expected size (~2 kbp) on 1% agarose gel (Figure 1a). This PCR fragment was incorporated successfully in cloning vector pBluescript II SK+, confirmed by colony PCR (Figure 1b) and restriction enzyme‐based digestion (Figure 1c). The sequence integrity of the cloned fragment was verified using Sanger sequencing. The *rolB* was then introduced into plant binary vector MpCAMBIA1301 from *rolB*‐pBluescript II SK + recombinant cloning vector. Successful gene incorporation into the former was confirmed by colony PCR of *E. coli* transformants using gene specific PCR primers (Figure 1d) and restriction enzyme digestion of recombinant *rolB*‐MpCAMBIA1301 (Figure 1e). These two vectors namely recombinant vector *rolB*‐MpCAMBIA1301 (Figure S1) and vector MpCAMBIA1301 were then transformed in *A. tumefaciens* LBA4404 using freeze–thaw method and transformants were selected by plating on LB plates supplemented with kanamycin.

### 3.2 Genetic transformation of *N.tabacum* with recombinant *rolB*‐MpCAMBIA1301

*A. tumefaciens* LBA4404 harboring plant binary vector *rolB*‐MpCAMBIA1301 and MpCAMBIA1301 (as vector control) were used to infect leaf explants of axenic *N. tabacum* plants using leaf‐disc method (Horsch et al., 1985). Excised leaf explants infected with strains *A. tumefaciens* LBA4404‐*rolB*‐MpCAMBIA1301 and *A. tumefaciens* LBA4404‐MpCAMBIA1301 and cultured on regeneration media exhibited swelling at leaf margins within 10–15 days and callus induction occurred within 20–30 days. Frequency of compact nodular callus induction was 40.9% and 36.20% for *rolB*‐MpCAMBIA1301 and MpCAMBIA1301 (vector control), respectively. Shoot bud induction occurred in regeneration media containing 25 mg L^−1^ Hygromycin B + 500 mg L^−1^ cefotaxime within 8 weeks. The microshoots (>1 cm) when excised and cultured on MS + 25 mg L^−1^ Hygromycin B + 500 mg L^−1^ cefotaxime, showed profuse root induction within 4 weeks (Figure S2). Fifteen putative *rolB* transgenic plant lines and four putative vector control plant lines from independent transformation events were subcultured on MS + 25 mg L^−1^ Hygromycin B + 500 mg L^−1^ Cefotaxime for 8 weeks. Profuse hairy roots developed from base of microshoots in 12/15 putative *rolB*‐transgenic plant lines. In vector control plant lines, roots developing from base of microshoots were non‐hairy and similar in phenotype as roots of untransformed plants, as expected (Sarkar & Jha, 2021). All transgenic plant lines and their clones were maintained in vitro on MS + 25 mg L Hygromycin B without phytohormones for over 3 years.

### 3.3 Confirmation of transformation in T_0_ plants

**FIGURE 2:**
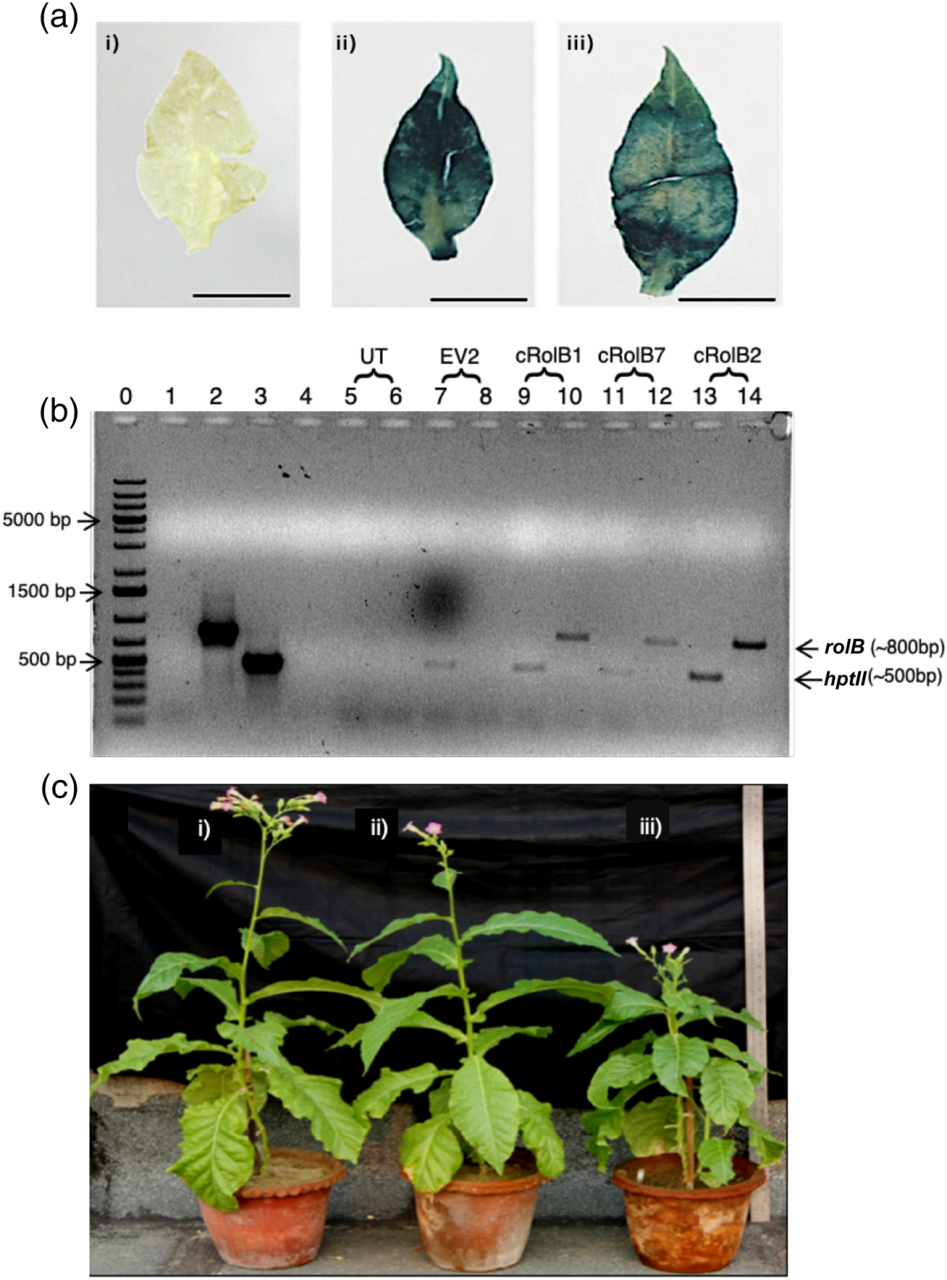
(a) Histochemical GUS staining of leaves from putative *rolB*‐transgenic and vector control‐transgenic plant lines (T_0_) for confirmation of transgene cassette integration (bar = 1.1 cm)—(i) leaf from non‐transformed *Nicotiana tabacum* ; (ii) leaf from MpCAMBIA1301 (vector control) transgenic, and (iii) leaf from *rolB‐*MpCAMBIA1301 transformed transgenic plant; (b) agarose gel electrophoresis representing PCR based confirmation of transgene integration in the putative transgenics. The DNA template was genomic DNA isolated from the leaves of plants under question. Lanes 1,3,5,7,9,11,13—PCR was done with *hpt*II primers; lanes 2,4,6,8,10,12,14—PCR was done with *rolB* primers. Lane 0—DNA ladder; lanes 1,2—*rolB*‐pBluescript II SK + (positive control for *rolB* ORF); lane 3,4‐MpCAMBIA1301 (positive control for *hpt*II ORF); lane 5,6‐ non‐transformed *N. tabacum* ; lane 7,8‐ MpCAMBIA1301 (vector control) transformed *N. tabacum* ; lane 9,10 ‐ *rolB*‐MpCAMBIA1301 transformed *N. tabacum* (cRolB1); lane 11,12*‐ rolB*‐MpCAMBIA1301 transformed *N. tabacum* (cRolB7); lane 13,14‐ *rolB*‐MpCAMBIA1301 transformed *N. tabacum* (cRolB2). DNA band size for *rolB* ORF: ~ 700 bp; for *hpt*II: ~500 bp. The presence of *hpt*II band for vector control transformed transgenic plants is expected while *rolB* transgenics are expected to be positive for both *rolB* ORF and *hpt*II; (c) *N. tabacum* T_0_ plant lines after 15 weeks of hardening (scale = 48 inches); (i) non‐transformed *N. tabacum* , (ii) *N. tabacum* transformed with MpCAMBIA1301 (vector control), (iii) *N. tabacum* transformed with *rolB‐*MpCAMBIA1301

The putative transgenic T_0_ plants (*rolB* and vector control transgenic plants growing in MS medium containing Hygromycin B [25 mg L^−1^]) were tested for the presence of transgene cassette by GUS staining, PCR and Reverse‐Transcriptase PCR (RT‐PCR). Young leaves from untransformed, vector control and *rolB*‐transgenic plant lines were used for GUS staining. Uniform GUS staining was observed in vector control and *rolB*‐transgenic plant lines but not in untransformed plants. Plants that were apparently chimeric (non‐uniformity of GUS staining) were discarded (Figure 2a). A total of 12 *rolB* transgenic plant lines and 2 vector control plant lines exhibiting uniform GUS staining were selected for further studies.

The integration and expression of *rolB* (780 bp ORF) in *rolB*‐transgenic plant lines were confirmed by PCR and RT‐PCR analysis performed with *rolB*‐ specific primers. A total of five *rolB* transgenic plant lines (T_0_) were selected for further studies after confirmation, namely, cRolB1, cRolB2, cRolB3, cRolB7, and cRolB8. The absence of *rolB* was confirmed in vector control plants EV2 and EV3 by PCR with *rolB* specific primers. PCR performed with primers specific for *hptII* confirmed the presence of transgene cassette in vector control and r*olB*‐transgenic plant lines. No amplification of *rolB* or *hptII* was observed in genomic DNA of non‐transformed plants (Figure 2b).

### 3.4 Ex‐vitro transfer and maintenance of T_0_, T_1_, and T_2_ transgenic *N. tabacum* plants

**FIGURE 3:**
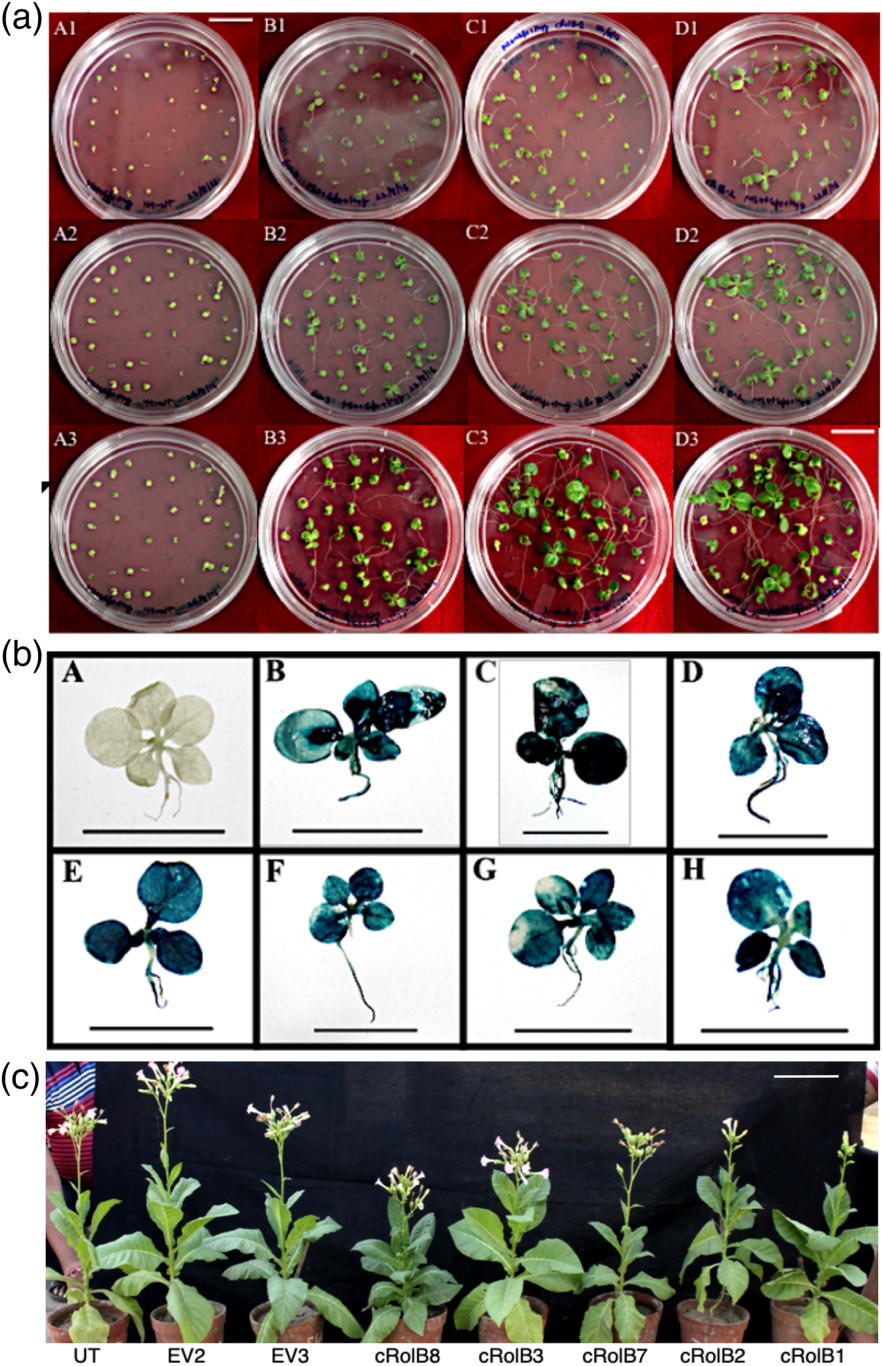
(a) *Nicotiana tabacum* seeds (T_1_) obtained from T_0_ plant lines plated on MS + Hygromycin (25 mg L^−1^) + cefotaxime (500 mg L^−1^). Plate series A1‐A3 (top to bottom)—Non‐transformed *N. tabacum* seeds, plate series B1‐B3 (top to bottom)—Seeds from MpCAMBIA1301‐transformed *N. tabacum* plant line (EV2), plate series C1‐C3—Seeds from *rolB*‐MpCAMBIA1301 transformed *N. tabacum* plant line (cRolB1), plate series D1‐D3—Seeds from *rolB*‐MpCAMBIA1301 transformed *N. tabacum* plant line (cRolB2). The numbers 1,2,3 denote 21,30 and 42 days after plating respectively (bar = 2 cms). The *rolB*‐transgenic T_1_ seedlings were observed to have longer roots than their vector control and non‐transformed counterparts; (b) histochemical GUS staining of 50 days old non‐transformed and transgenic T_1_ *N. tabacum* seedlings; (a)—Non‐transformed *N. tabacum* , (B) and (C) EV2 and EV3 (vector control) T_1_ seedlings; (D) *rolB*‐transgenic cRolB1; (E) *rolB*‐transgenic cRolB2; (F) *rolB*‐transgenic cRolB3; (G) *rolB*‐transgenic cRolB7 and (H) *rolB*‐transgenic cRolB8 (bar = 1 cm); (c) *rolB*‐transgenic *N. tabacum* T_1_ plant lines along with non‐transformed and vector control T_1_ plants taken after 23 weeks. From left to right—UT (untransformed/non‐transformed), EV2 (vector control 2), EV3 (vector control 3), cRolB8, cRolB3, cRolB7, cRolB2, cRolB1 (*rolB* transgenic plant lines), scale—6 inches

Axenically raised T_0_ *rolB*‐transgenic and T_0_ vector control plant lines were acclimatized and established in greenhouse. The *rolB* transgenic T_0_, T_1_, and T_2_ plant lines were observed to be shorter in height and bushy, as compared to T_0_ transgenic vector control plant lines and non‐transformed plants (Figure 2c). Seeds collected from each T_0_ *rolB*‐transgenic plant line and T_0_ vector control plant lines were plated on MS medium with hygromycin B (25 mg L^−1^) for selection of *rolB* transgenic T_1_ seedlings (Figure 3a).

It was observed that in MS medium (without Hygromycin B), germination frequency of seeds from T_0_ *rolB*‐transgenic plant lines, T_0_ vector control plants and seeds from non‐transformed plants was 100%. Seeds from non‐transformed plants failed to germinate in MS medium containing Hygromycin B (25 mg L^−1^). In T_0_ *rolB*‐transgenic plant lines, the frequency of seeds resistant to hygromycin B in MS medium varied between the five lines (Table 1). The vector control plant line EV2 and *rolB*‐transgenic plant line cRolB2 exhibited no significant deviation from Mendelian ratio of 3:1 (*p* < .05). Out of the remaining four *rolB*‐transgenic plant lines which exhibited significant deviation from 3:1 ratio, three *rolB*‐transgenic plant lines (cRolB3, cRolB7, cRolB8,) showed no significant deviation from 2:1 (*p* < .05) and one *rolB*‐transgenic plant line (cRolB1) showed a segregation ratio of 6:1 (Table 1). Germination frequency of seeds from non‐transformed plants in presence and absence of Hygromycin B (0% and 100% respectively) and germination frequency of *rolB*‐transgenic and vector control plants in absence of hygromycin (100%) suggest that mechanical factors did not affect germination frequency of seeds from transgenic T_0_ plants.

**TABLE 1:** Segregation pattern of Hygromycin B resistance in T_1_ progenies of *rolB*‐transgenic T_0_ plant lines

Seeds from plant line (T_0_)	Number of seeds plated on (MS + 25 mg L^−1^ Hygromycin B) resistant/sensitive to Hygromycin B (Hyg^R^/Hyg^S^)	Segregation ratio Hyg^R^:Hyg^S^		*χ* ^2*^
		Observed	Expected	
Nontransformed	0/175	0:175	3:1	525
Vector control (EV2)	93/41	2.26:1	3:1	2.23
*rolB* transgenic plant line cRolB1	163/26	6.2:1	6:1	0.04
*rolB* transgenic plant line cRolB2	121/45	2.68:1	3:1	0.39
*rolB* transgenic plant line cRolB3	65/46	1.41:1	2:1	3.28
*rolB* transgenic plant line cRolB7	66/34	1.94:1	2:1	0.02
*rolB* transgenic plant line cRolB8	60/36	1.66:1	2:1	0.75

*Note*: Resistance and sensitivity of T_1_ progenies to Hygromycin B evaluated in terms of segregation ratio and tested for goodness of fit to expected ratio using Chi‐Square test. The critical value is 3.84. χ^2*^ = 3.84 (*p* value threshold <.05).

Transgenic *rolB* (T_1_) plant lines and vector control (T_1_) plant lines which grew well on MS + Hygromycin (25 mg L^−1^) (Figure 3a) were confirmed for integration of the transgene cassette by GUS staining (Figure 3b). The uniformity of GUS staining was observed in all five *rolB*‐transgenic (T_1_) plant lines and two vector control transgenic (T_1_) plant lines suggesting that the integration was successful and had occurred throughout the plant system. Seeds were collected from *rolB* transgenic T_1_ plant lines and vector control plant after transfer to greenhouse (Figure 3c) and plated on MS + Hygromycin B (25 mg L^−1^). The seeds (T_2_) collected from T_1_ *rolB*‐transgenic plant lines and T_1_ vector control plant lines were plated on MS + 25 mg L^−1^. The seedlings of T_2_ *rolB*‐transgenic plant lines and vector control plant lines were subjected to GUS to detect the presence of transgene cassette. All five *rolB* plant lines and two vector control plant lines were found to show uniform GUS staining suggesting presence of the transgene cassette. (Figure S3).

### 3.5 Confirmation of expression of *rolB* in *rolB*‐transgenic T_1_ and T_2_ plant lines

**FIGURE 4:**
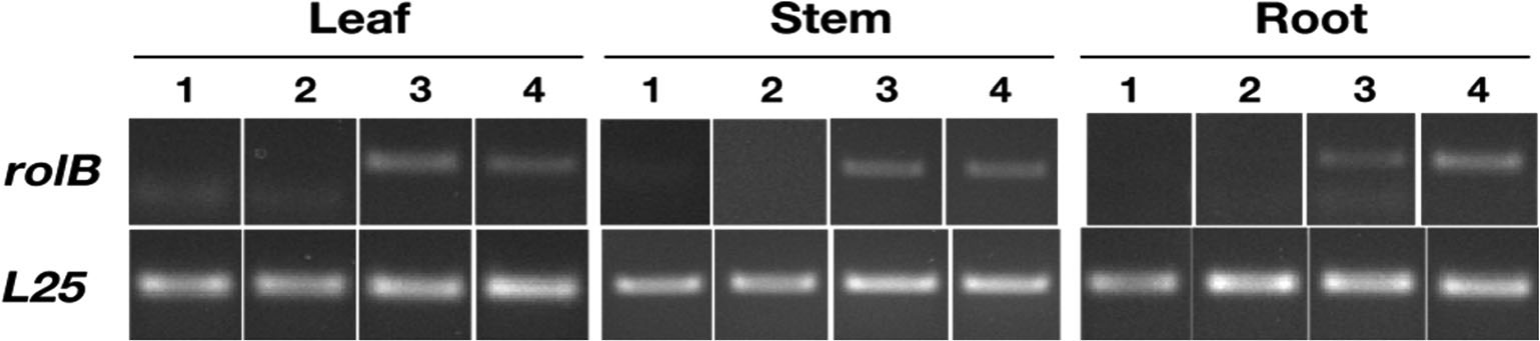
Expression of *rolB* in three different tissues of *N. tabacum* T_1_ plants evaluated using reverse transcriptase‐PCR; lane 1—Non‐transformed *N. tabacum* , lane 2—MpCAMBIA1301 (empty vector) transformed *N. tabacum* , lane 3—*rolB*‐MpCAMBIA1301 transformed transgenic plant line (cRolB1), lane 4—*rolB*‐MpCAMBIA1301 transformed transgenic plant line (cRolB2). Primers pairs specific for *rolB* and *L25* (60S ribosomal protein L25, endogenous control) were used

The expression of *rolB* in *rolB*‐transgenic T_1_ plant lines was confirmed by Reverse Transcriptase (RT‐PCR) with *rolB* specific primers. The cDNA was prepared from RNA extracted from leaves, stem and root of *rolB*‐transgenic T_1_ plant lines. The *rolB* expression was confirmed in leaf, stem and root of five T_1_ *rolB*‐transgenic plant lines (Figure 4).

### 3.6 Identification and retrieval of context‐relevant *ARF*s via datamining of gene ontology records

The *ARFs* exist as multiple members of a large multigene TF family (*ARF*/IAA) which perform specific but overlapping functions. However, to identify *ARFs* involved in hairy root development, the Gene Ontology database obtained from TAIR website specific to *A. thaliana*, was mined. Several successive steps of progressive filtering based on relevant keywords—“root” and “transcription factor” (Figure S4) led to the selection of 102 TFs (and TF like) which are known to be involved in root development. These were the TF genes which were known to be “root‐related.” Since TFs may belong to different functional protein families, the protein sequences of 102 TFs were retrieved and subjected to tree‐based clustering. This led to categorization of TFs into 12 broad groups based on the presence of known protein conserved functional domains (Figure S5, Table S3(D)). These 12 groups were bHLHs, DOF, IDD, Aux/IAA, WRKY, NAC, MYB, AP2, ARR, HD‐Zip I, MADS, and *ARF*. All the *ARF*s annotated to be associated with root‐related classes as per Gene Ontology evidence, were thereby identified with this clustering‐based approach and were considered relevant to our context. The group *ARF* from the clustering was observed to be constituted of six TFs, and their functional roles in root development were verified from literature evidence. Later, two more TFs were added to the study owing to further availability of experimental evidence(s). Hence, eight *ARF* TFs were finalized for further study. These were At*ARF5* (AT1G19850), At*ARF6* (AT1G30330), At*ARF7* (AT5G20730), At*ARF8* (AT5G37020), At*ARF10* (AT2G28350), At*ARF16* (AT4G30080), At*ARF17* (AT1G77850), and At*ARF19* (AT1G19220).

### 3.7 Gene expression profiling of *ARF*s identified from data mining by real‐time PCR in *rolB* transgenic and vector control plants

**FIGURE 5:**
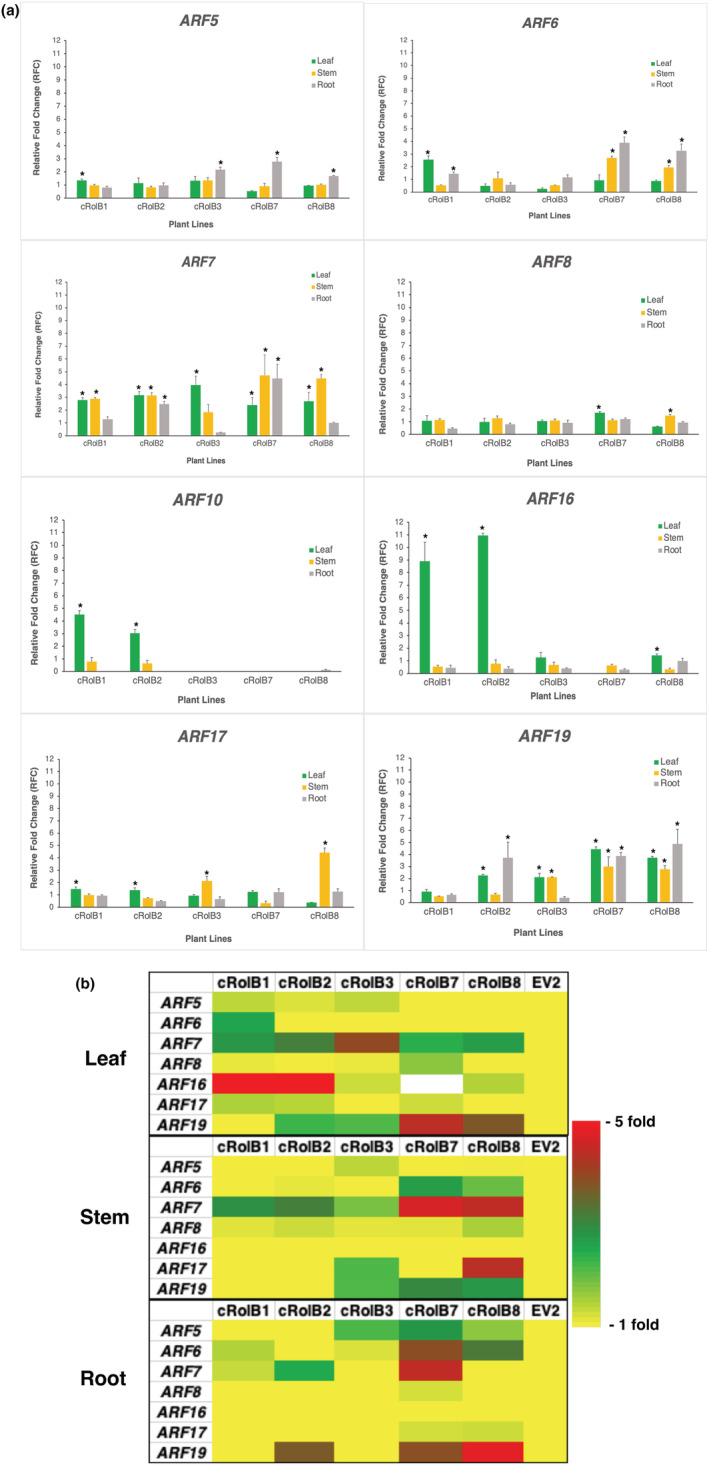
Gene expression profiling of *NtARF*s in different tissues of five *rolB* transgenic and vector control plant lines (T_1_) represented as barplot (5a) and heat map (5b)‐ (a) transcript levels are given as relative values to vector control EV2 (the value of 1), after being normalized to 60S ribosomal protein L25 (*L25*) gene. Data are shown as the means with standard error in five *rolB* transgenic plant lines (cRolB1, cRolB2, cRolB3, cRolB7, cRolB8) over two technical replicates of five biological replicates. The * denotes statistical significance of gene upregulation at *p* value <.05; (b) ‐ the scale for heat map represents the relative fold change of expression (from onefold [unchanged] to fivefold change [upregulated])

The *N. tabacum* homologs of the eight At*ARF*s as identified from data mining were assessed in terms of general trend of gene expression in three types of tissues (leaf, stem and root) in five *rolB* transgenic plant lines and one vector control plant line. The relative gene expression fold change against vector control plant line was calculated for each *ARF* and represented as barplot (Figure 5a) and heatmap (Figure 5b). It was observed that across all tissue types, Nt*ARF7* and Nt*ARF19* were found to be differentially upregulated in most of the *rolB*‐transgenic T_1_ plant lines as compared to vector control plant line (Figure 5a,b). Significant upregulation was observed in 5/5 (*ARF7*) and 4/5 (*ARF19*) of the transgenic T_1_ plant lines tested. In case of *ARF7*, significant upregulation was observed in 11/15 test cases—leaf (5/5), stem (4/5), and root (2/5). For *ARF19*, significant upregulation was observed in 10/15 test cases—leaf (4/5), stem (3/5) and root (3/5). Gene expression pattern of other *ARF*s, Nt*ARF5*, Nt*ARF6*, Nt*ARF8*, Nt*ARF10*, Nt*ARF16*, and Nt*ARF17*, were not found to exhibit consistent differential gene upregulation across the three tissue types in five *rolB*‐transgenic plant lines. For example, number of test cases showing significant upregulation ranged from 6/15 in *ARF6* to 2/15 in *ARF8* and *ARF10*.

### 3.8 Construction of gene ontology‐based transcriptomic network maps driven by *ARF*s

A transcriptomic‐network map comprising of genes driven by Nt*ARF7* and Nt*ARF19* was developed. Genes which could be potential downstream targets of Nt*ARF7* and Nt*ARF19* were identified by application of the following strategy—downstream target genes are likely to be (i) co‐expressed spatially and temporally with *ARF*s and (ii) they possess the AuxRE (Auxin Responsive Elements) binding motifs in their upstream elements. To find such target genes, genes co‐expressed with the *ARF*s were identified and retrieved from the CORNET co‐expression online database. It was assumed that as auxin and *ARF* orthologs are ubiquitous in all land plants, the signaling network would exist similarly in *N. tabacum* by principle of conservation of gene function. The CORNET online database mining provided an exhaustive list of 325 genes which are co‐expressed with At*ARF7*‐At*ARF19* TFs (Table S4). Subsequently, the 3000 bp upstream sequences of these 325 genes were scanned using FIMO for the presence of at least one AuxRE binding motifs and as a result 149 genes were obtained (Table S5). These genes were functionally clustered on the basis of their “biological process” (Gene Ontology) using BiNGO plugin of Cytoscape (Figure S6).

**FIGURE 6:**
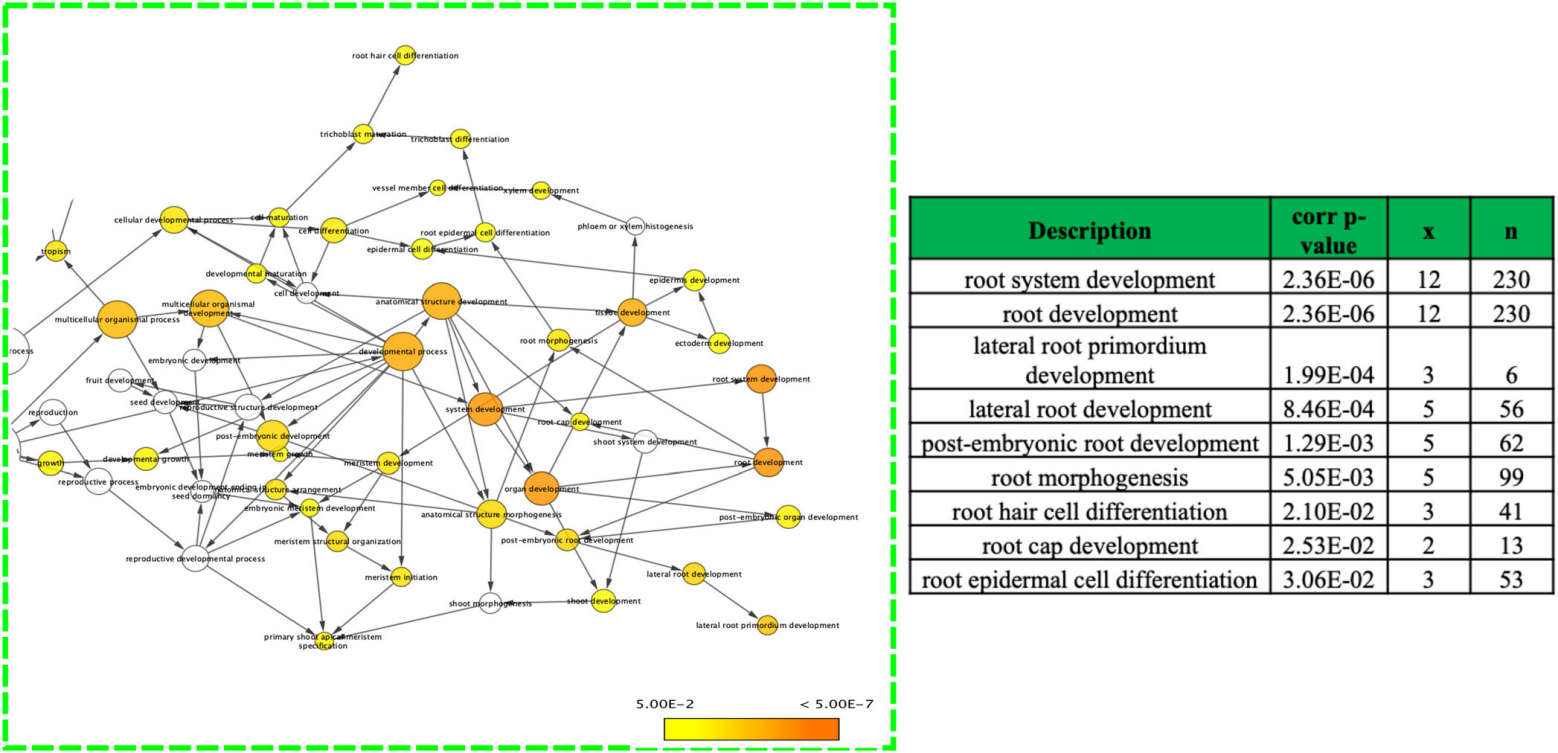
A close‐up of cluster involved in organ‐development and morphogenesis related functions represented by the network map of AtA*RF7*‐At*ARF19* co‐expressed and AuxRE‐motif possessing genes, with the green circle highlighting clusters involved in organ development and morphogenesis‐related functions. Multiple root‐development and morphogenesis related functions were identified with significant correlated *p* values (*p* < .05)

The 149 genes were observed to constitute a map with multiple nodes exhibiting high enrichment for certain biological processes (Figure S7). Broad classes of biological processes such as “organ development,” “morphogenesis‐related,” and “metabolic process” were observed, but interestingly within the “organ development” supercluster, several “root organ related” biological processes were found and appeared to be significant (based on correlation *p* values) as reported by BiNGO (Table S6). For instance, two of the nodes/cluster which appeared to be highly significant based on the correlated *p* value and the high number of gene members were found to be “root system development” (*x* = 12/*n* = 230, *p* value = 2.36E−06) and “root‐development” (*x* = 12/*n* = 230, *p* value = 2.36E−06). Several other root‐development related classes were also found such as “lateral root primordium development,” “root morphogenesis,” “trichoblast maturation,” “root hair cell differentiation,” and so forth in this *ARF7‐ARF19* based network map (Figure 6). On further investigation, no significant clusters/nodes with functional enrichment related to other type of tissue development such as flower, seed, pollen etc. were observed.

**FIGURE 7:**
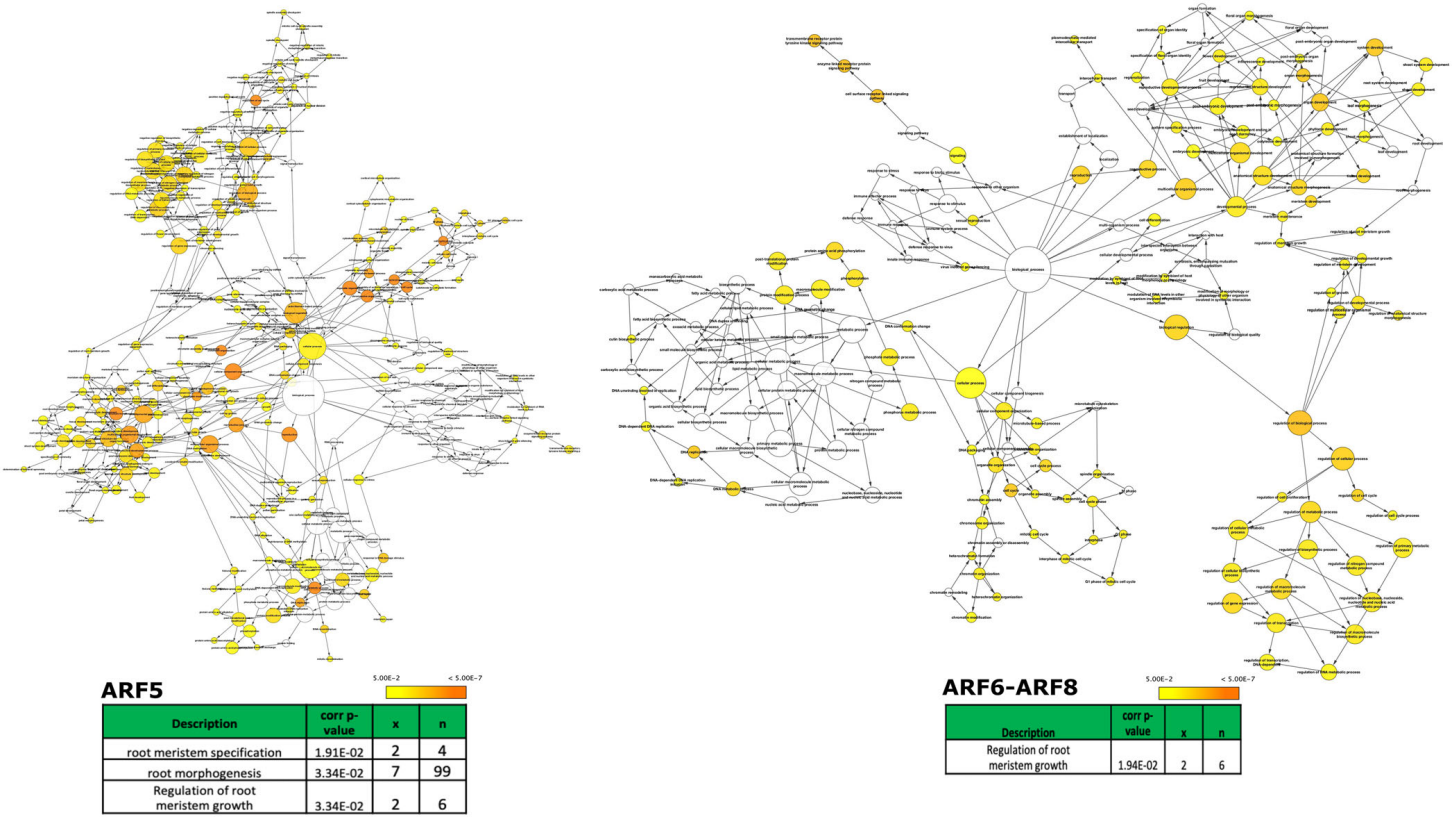
Comparison between different gene‐ontology based network maps built from query *ARF* co‐expressed and AuxRE motif possessing genes. Two separate maps were built similarly but with different query *ARF*s—At*ARF5* (left, top) and At*ARF6*‐At*ARF8* (right, top). The results were observed to be strikingly different from At*ARF7‐*At*ARF19* map—Fewer number of root‐development and morphogenesis related “biological process” clusters were identified in At*ARF5* and At*ARF6‐AtARF8* maps (bottom), suggesting that At*ARF7*‐At*ARF19* might perform dominant and underappreciated roles in root‐morphogenesis related processes as compared to At*ARF5* and At*ARF6*‐At*ARF8*. Scale represents significance of correlated *p* values

In order to test the hypothesis regarding At*ARF7*‐At*ARF19* exclusivity to activation of root‐development pathways in *rolB*‐transgenic background, similar network maps were built for the non‐upregulated At*ARF5* and At*ARF6*‐At*ARF8* following the same methodology and the newer maps were compared. The results were strikingly different—a larger number of co‐expressed genes with AuxRE motifs were found for At*ARF5*, At*ARF6*‐At*ARF8*; but their network maps exhibited much lower number of nodes/clusters dedicated to root‐development related functions with significant enrichment scores (Figure 7). For instance, BiNGO reported only three root‐development related nodes/clusters even for an input list of 616 At*ARF5* co‐expressed and AuxRE‐motif possessing (in upstream elements) genes, while only one root‐development related node was reported for an input list of 331 At*ARF6*‐At*ARF8* co‐expressed and AuxRE‐motif possessing genes (Table 2, Table S6). It was also observed that unlike At*ARF7*‐At*ARF19* network map, nodes/clusters dedicated to non‐root tissue types such as “leaf,” “flower,” “pollen,” and “seed” were present in both At*ARF5* and At*ARF6*‐At*ARF8* network maps with a predominance of nodes dedicated to general biological processes such as “cell‐division,” “DNA metabolism,” and “regulation of biological processes.”

**TABLE 2:** Summary of gene ontology based transcriptomic network map generation exercise comprising of putative targets of *ARF*s (co‐expressed with ARFs and possessing AuxREs)

	At*ARF5*	At*ARF6*‐At*ARF8*	At*ARF7*‐At*ARF19*
Co‐expressed genes (CORNET)	1463	759	325
Co‐expressed (CORNET) and AuxRE motifs possessing genes (FIMO)	616	331	149
Number of nodes/clusters reported by BiNGO	225	110	95
Number of “root”‐related nodes	3	1	9

*Note*: Results from the map building exercise. At*ARF5* and At*ARF6*‐At*ARF8* were found to have a higher number of co‐expressed aux‐RE possessing genes, but a far lower number of root‐development related nodes as compared to At*ARF7*‐At*ARF19*.

## 4 DISCUSSION

### 4.1 *rolB*‐related phenotypic traits are inherited by T_1_ progeny

A functional non‐chimeric *rolB*‐transgenic background was established as first part of this study. *rolB* from *A. rhizogenes* A4 was successfully cloned into a plant binary vector followed by genetic transformation of axenically raised *N. tabacum* plants with the former. During the development and maturation of *rolB*‐transgenic T_0_ plant lines, phenotypes which are characteristic of *rolB*'s effect on plant development and reported previously were observed such as dwarfism and enhanced root growth with bushy appearance (Altvorst et al., 1992; Kodahl et al., 2016; Schmülling et al., 1988). It is because of these phenotypes that identification of *rolB*‐transgenic plants became easier at mature stages. Chimerical plants were screened out using GUS staining. During growth and subsequent maturation of these T_1_ plant lines, the phenotypes observed in T_0_ generation i.e. dwarfism with bushy appearance and enhanced root growth were also observed in T_1_ generation as reported earlier (Schmülling et al., 1988).

Gene expression study using Reverse‐Transcriptase PCR (RT‐PCR) was conducted in transgenic T_1_ generation plant lines (from T_0_ seeds). *rolB* expression was detected in all transgenic T_1_ plant lines confirming that the *rolB* within the transgene cassette was not lost or rendered non‐functional transcriptomically due to transgene silencing via position effect or epigenetic modification during seed development. The T_2_ generation also manifested similar phenotypes. Hence, it was inferred that *rolB*‐related phenotypes are inheritable till T_2_ generation.

In these transgenic T_1_ progenies derived from five *rolB*‐transgenic plant lines (T_0_) and vector control plant lines (T_0_), Hygromycin B resistance was observed to segregate with the expected ratio of 3:1, but deviations were also found. The causal factors for deviation from Mendelian segregation ratio of 3:1 cannot be explained at present. It could be due to non‐segregating progenies (Tizaoui & Kchouk, 2012). Segregation ratios in subsequent generations will be studied to draw conclusions which may lead to clarification regarding the expected and observed segregation ratios.

### 4.2 Identification of context relevant TFs for the study—The *ARF*s

Numerous studies have proposed the mechanism of RolB action – from perturbation of intracellular auxin concentration (Estruch, Schell, & Spena, 1991) to molecular binding with Nt14‐3‐3‐like protein ωII (Moriuchi et al., 2004). Nevertheless, it is well known that auxin perception is heightened/enhanced (Delbarre et al., 1994) and auxin‐related responses are affected (Cardarelli et al., 1987, b; Michael & Spena, 1995; Schmülling et al., 1993; Spena et al., 1987) apart from induction of hairy roots in *rolB*‐transgenic plants. However, it was understood that hairy root development, the context in focus, must be driven by multiple genes which are transcriptionally activated or repressed. Hence, existence of any functional link between auxin and *rolB* via *ARF*s, which are auxin‐related TFs, was assessed. This perspective had the potential to uncover and understand (a) specific auxin‐mediated transcriptomic responses/pathways leading to *rolB*‐mediated hairy root development, (b) how other *rolB*‐associated phenotypes are triggered, in *rolB*‐transgenic background. Since auxin‐mediated transcriptomic responses/pathways are enormously complicated, the scope in this study was restricted to identification of the TFs (*ARF*s) possibly influenced in *rolB*‐transgenic background. The following known facts exemplify the previous statement—there are 22 homologs of *ARF*s discovered in *A. thaliana* (Li et al., 2016) and they are known to express according to external and internal stimuli (Ha et al., 2013; Li et al., 2015; Sun et al., 2015; Wang et al., 2007, 2011). Furthermore, these *ARF*s interact with themselves and their cognate Aux/IAA binding partners in a temporal–spatial context specific manner which is an already known complicated affair (Kim et al., 1997; Piya et al., 2014; Ulmasov et al., 1999a; Weijers et al., 2005). Needless to say, control of auxin related‐signaling network is exerted via multiple levels of transcriptional and post‐translational processes (Leyser & Berleth, 1999). Still, this perspective was pursued because of two reasons—(a) no study yet exists which probed the impact of *rolB* on the auxin‐signaling transcriptomic network providing clues to functional link between *rolB* and auxin, no matter how limited it might be and (b) identification of specific and selective transcriptomic pathways, if discovered, may serve as starting point for future studies.

Numerous studies have proposed the mechanism of RolB action – from perturbation of intracellular auxin concentration (Estruch, Schell, & Spena, 1991) to molecular binding with Nt14‐3‐3‐like protein ωII (Moriuchi et al., 2004). Nevertheless, it is well known that auxin perception is heightened/enhanced (Delbarre et al., 1994) and auxin‐related responses are affected (Cardarelli et al., 1987, b; Michael & Spena, 1995; Schmülling et al., 1993; Spena et al., 1987) apart from induction of hairy roots in *rolB*‐transgenic plants. However, it was understood that hairy root development, the context in focus, must be driven by multiple genes which are transcriptionally activated or repressed. Hence, existence of any functional link between auxin and *rolB* via *ARF*s, which are auxin‐related TFs, was assessed. This perspective had the potential to uncover and understand (a) specific auxin‐mediated transcriptomic responses/pathways leading to *rolB*‐mediated hairy root development, (b) how other *rolB*‐associated phenotypes are triggered, in *rolB*‐transgenic background. Since auxin‐mediated transcriptomic responses/pathways are enormously complicated, the scope in this study was restricted to identification of the TFs (*ARF*s) possibly influenced in *rolB*‐transgenic background. The following known facts exemplify the previous statement—there are 22 homologs of *ARF*s discovered in *A. thaliana* (Li et al., 2016) and they are known to express according to external and internal stimuli (Ha et al., 2013; Li et al., 2015; Sun et al., 2015; Wang et al., 2007; Wu et al., 2011). Furthermore, these *ARF*s interact with themselves and their cognate Aux/IAA binding partners in a temporal–spatial context specific manner which is an already known complicated affair (Kim et al., 1997; Piya et al., 2014; Ulmasov et al., 1999a; Weijers et al., 2005). Needless to say, control of auxin related‐signaling network is exerted via multiple levels of transcriptional and post‐translational processes (Leyser & Berleth, 1999). Still, this perspective was pursued because of two reasons—(a) no study yet exists which probed the impact of *rolB* on the auxin‐signaling transcriptomic network providing clues to functional link between *rolB* and auxin, no matter how limited it might be and (b) identification of specific and selective transcriptomic pathways, if discovered, may serve as starting point for future studies.

With this perspective, the *ARF*s, which have enhanced or altered expression status in *rolB*‐transgenic background were identified. TFs such as *ARF*s are primarily involved in expressing/repressing genes which subsequently drive gene‐associated changes and it was assumed that a change in gene expression stoichiometry of the former might possibly lead to differential gene expression of their target genes (Okushima et al., 2007). This led to the question—How to identify the relevant *ARF*s since multiple *ARF*s are known to exist as a multigene family (Guilfoyle et al., 1998)? An approach was adopted—as the root‐inducing capability of *rolB* was known, TFs (not just *ARF*s) which had a root‐development related role and/or expressed in roots were mined—this was the criterion of relevance to this study. It was expected that this broader approach would lead to the identification of all the relevant *ARF*s. The Gene Ontology database was mined in silico for “relevant” TFs, and their functional categorization led to the discovery of multiple TF families (Figure S5). This served as an exciting prospect, as no such study known has yet explored the publicly available databases in such manner, though later it was observed that individual genes belonging to these families have been studied previously (Alvarez‐Buylla et al., 2019; Montiel et al., 2004); 102 genes belonging to 12 protein families were thus identified, and six *ARF*s were identified through this clustering approach. Inspection of this tree also revealed several other TFs involved in different aspects of root development suggesting the value of this exhaustive approach. For example, multiple proteins were found belonging to following domain families—bHLHs such as *SCR* (AT3G54220) which regulates radial organization of root (Laurenzio et al., 1996), IDD‐domain such as *NUTCRACKER* (AT5G44160) required for root patterning (Long et al., 2015), NAC domain such as *FEZ* (AT1G26870) involved in root‐cap development (Willemsen et al., 2008), MYB domain such as *WEREWOLF* (AT5G14750) involved in root cell fate determination (Song et al., 2011) and AP2 such as *PLETHORA 1* (AT3G20840) essential for quiescent center activity (Aida et al., 2004) were all observed within this tree. Hence, this approach appeared to be useful in identification and retrieval, exhaustively, of all the *ARF*s which were relevant to the context. The six *ARF*s thus identified were At*ARF19* (AT1G19220) (Okushima et al., 2007; Wilmoth et al., 2005), At*ARF7* (AT5G20730) (Okushima et al., 2007; Wilmoth et al., 2005), At*ARF5* (AT1G19850) (Berleth & Jürgens, 1993; Przemeck et al., 1996), At*ARF17* (AT1G77850) (Gutierrez et al., 2009), At*ARF10* (AT2G28350) (Wang et al., 2005), and At*ARF16* (AT4G30080) (Wang et al., 2005). Later, two more *ARF*s, At*ARF6* (AT1G30330) (Gutierrez et al., 2009) and At*ARF8* (AT5G37020) (Gutierrez et al., 2009), were added from literature survey. Subsequently, the gene expression levels of *N. tabacum* orthologs of these *ARF*s was assessed in *rolB*‐transgenic *N. tabacum* background.

### 4.3 Nt*ARF19* and Nt*ARF7* are differentially upregulated in *rolB*‐transgenic background

Two interesting questions arose—Do all eight *ARF*s exhibit differential gene expression under the influence of *rolB* or only a few of them? Moreover, which *ARF*s initiators (*ARF5*, *ARF6*, *ARF7*, *ARF8*, *ARF19*; Roosjen et al., 2018; Ulmasov et al., 1999a, 1999b) or repressors (*ARF10*, *ARF16*, *ARF17*; Roosjen et al., 2018; Tiwari et al., 2003) are differentially expressed in *rolB*‐transgenic background? These questions had the potential to answer specificity of *rolB*‐mediated responses. For example, *ARF5* is known to be important for development of root‐shoot axis during embryo growth (Berleth & Jürgens, 1993; Przemeck et al., 1996) while *ARF6* and *ARF8* have been reported to be involved in adventitious root development (Gutierrez et al., 2009). Real‐time PCR experiments were performed to assess the differential gene expression of these *ARF*s and scored over test cases, inclusive of three tissue types. It was observed that out of all eight Nt*ARF*s tested, Nt*ARF7* and Nt*ARF19* showed a general trend of upregulation across all tissues in most of the greenhouse‐grown T_1_ plant lines. For the other six Nt*ARF*s, no comprehensible gene expression pattern/trend were observed. Variability observed in gene expression among tissue types and transgenic plant lines may be due to spatially heterogenous growth conditions (unlike homogenous tissue‐culture conditions) when growing in a greenhouse leading to inter‐line variations (Poorter et al., 2012, 2016) or due to *rolB*'s impact or both. However, the two genes Nt*ARF7* and Nt*ARF19* were found to exhibit distinctive pattern of gene expression upregulation. The upregulated *ARF*s *ARF7* and *ARF19* are known to be key players in lateral root development (Okushima et al., 2007; Wilmoth et al., 2005). They are reported to directly regulate transcription of genes such as *LATERAL ORGAN BOUNDARIES‐DOMAIN16/ASYMMETRIC LEAVES2‐LIKE 18* (*LBL16/ASL18*) which lead to lateral root formation. Hence, it was inferred that in *N.tabacum rolB*‐transgenic T_1_ background, Nt*ARF7* and Nt*ARF19* gene expression is upregulated and they might have a key role to play in *rolB*‐driven auxin‐mediated responses by activating the corresponding downstream target genes of the former.

### 4.4 Nt*ARF19*/Nt*ARF7* gene‐ontology based transcriptomic maps have numerous “root‐related” biological processes categories

The observation of Nt*ARF7* and Nt*ARF19* gene expression upregulation in *rolB*‐transgenic T_1_ plant lines prompted two major questions: (a) How does this selective upregulation of two Nt*ARF*s affect the plant system? (b) Can the selective upregulation of these two Nt*ARF*s result in hairy root‐development which is particular to *rolB*‐transgenic background? The first question is very broad and difficult to address while the second, although narrow in scope and relevant to this study, appeared equally intractable. This is because it was understood that Nt*ARF7* and Nt*ARF19* being TFs might activate (transcriptionally) multiple genes (maybe thousands) which may interact in a complicated fashion.

To address this issue, it was realized that exploring transcriptomic networks is the best solution, at least initially. But since such transcriptomic networks specific to *rolB*‐transgenic background or Nt*ARF*‐specific context were not available, it was decided to construct networks comprising of “target genes” of such Nt*ARF*s. The following approach was adopted—(a) first, genes which co‐express with *ARF*s spatially and/or temporally were identified, (b) second, the genes should have the AuxRE motifs in their promoter/upstream elements, and finally, (c) an ontology map based on “biological function” using these “target genes” was constructed. This strategy is quite simple—if a gene is activated/upregulated by *ARF*s, it will be co‐expressed “by‐definition” and unless the former possesses an AuxRE motif in its promoter/upstream elements, it cannot be upregulated by *ARF*s (Boer et al., 2014; Hagen & Guilfoyle, 2002; Ulmasov, 1997). 149 genes were found satisfying both criteria (co‐expression with *ARF7*/*ARF19* + AuxRE motif possession) and a biological‐process network‐map using BiNGO (a cytoscape plugin) was constructed. It is to be noted here that since *ARF7* and *ARF19* are reported to work together as a module (Okushima et al., 2007), both were considered together when mining for co‐expressed genes. Additionally, due to conservation of auxin function in land plants, an equivalence of function between *ARF*‐driven gene expression networks of *A. thaliana* and *N. tabacum* was assumed. Analysis of this network map built on 149 target genes revealed that *ARF7* and *ARF19* have multiple nodes representing biological processes dedicated to different aspects of development including root‐development. These multiple root‐development processes nodes were identified and were found to be significant (Figure 6). Closer inspection of these nodes suggested genes which had indispensable roles to play in root development. For example, the “root‐hair cell differentiation” node found was comprised of three genes, two of which At*MYA2* and At*CSLD2* are known to be involved in root hair growth (Bernal et al., 2008) and root hair elongation (Peremyslov et al., 2008), respectively. From these results, it was initially inferred that At*ARF7‐*At*ARF19* may have major deterministic roles in activating root‐development processes. Interestingly, it is also known that At*ARF7*‐At*ARF19* are involved in lateral root formation (Okushima et al., 2007). However, the other chosen *ARF*s are also known to be involved in certain aspects of root development; for example, At*ARF5* is known to be critical for morphological root‐shoot axis establishment (Berleth & Jürgens, 1993; Mayer et al., 1991), so the observation was extended to other *ARF*s to assess whether they exhibited major root related developmental roles as well. Similar maps for At*ARF6*‐At*ARF8*, At*ARF5* were developed, and it was surprisingly observed that although the newer maps (based on *ARF6‐ARF8* and *ARF5*) had a far large number of participating target genes, they had conspicuously lower number of root‐development related biological processes (Figure 7). Rather, the newer maps (based on *ARF6‐ARF8* and *ARF5*) had predominance of non‐root tissue types such as “leaf” and “flower” (Table S6). This suggests that *rolB*'s role in development of hairy roots might possibly be due to selective upregulation of *ARF7‐ARF19*.

Hence, in conclusion, it is proposed that in *rolB*‐transgenic background, Nt*ARF7*‐Nt*ARF19* are selectively but differentially upregulated by a yet‐undiscovered mechanism, which might drive hairy root development. It is also postulated that the likely deterministic, yet‐underappreciated role of *ARF7‐ARF19* in root development as inferred from the network maps might be the cause of highly specific hairy root‐phenotype associated with *rolB*—this might be the functional transcriptomic link between *rolB* and auxin. Lastly, it is emphasized that the network maps presented in this study may serve as starting points to understand in greater detail, the nature of transcriptomic events occurring in *rolB*‐transgenic background leading to development of traits characteristic of *rolB*‐transgenic plants.

## CONFLICT OF INTEREST

The Authors did not report any conflict of interest.

## Supporting information


**Figure S1** Recombinant *rolB* transgene cassette constructed within MpCAMBIA1301 vector: A 2019 bp fragment harboring *rolB*_TL_ was incorporated between *Sma*I and *Sal*I restriction enzyme sites within the MpCAMBIA1301 vector.

**Figure S2** Regeneration and maintenance of *rolB* transgenic plants (T_0_) in vitro ‐ a**)** Axenic leaf explants ( *Nicotiana tabacum* ) after co‐cultivation with *Agrobacterium tumefaciens* ‐*rolB*‐MpCAMBIA1301 on shoot regeneration medium (MS + BAP_2_ + Cef_500_ + Hyg_25_); **b)** Shoot bud induction on shoot regeneration medium (MS + BAP_2_ + Cef_500_ + Hyg_25_); **c)** Microshoots on MS without phytohormones but with Cef_500_ + Hyg_25_; **d)** and **e)** –*rolB*‐transgenic plant line (cRolB2) showing profuse root growth. The subscripts indicate antibiotic concentration in mg l^−1^. Abbr. ‐ Cef – Cefotaxime, Hyg – Hygromycin, MS medium – Murashige‐Skooge, BAP – Benzylaminopurine. White bar is scale – 2 cms.

**Figure S3** **Histochemical GUS staining of leaves of *rolB*‐transgenic T**_**2**_ **and vector control T**_**2**_ **seedlings confirming the presence of transgene cassette**. From left to right – Leaves of plant line UT (Untransformed/wildtype), EV2 (vector control 2), *rolB‐*transgenic plant lines cRolB1, cRolB2, cRolB3, cRolB7, cRolB8 (*rolB* transgenic plants, bar = 1 cm).

**Figure S4** **Results of the datamining performed on the gene ontology record file obtained from TAIR website represented as a Venn diagram** ‐ Root‐related transcription factors (n = 102) were found by the intersection of two sets.

**Figure S5** **Clustering of transcription factors into broad classes of protein families via tree building (Neighbor Joining method) using protein sequences (n = 102) corresponding to genes identified from datamining of gene ontology file** ‐ The Auxin Response Transcription factor group (*ARF*) was identified as the third group in the tree (from top).

**Figure S6** **Schematic representation of the workflow adopted to propose an *ARF* driven transcriptomic signaling network** ‐ The initial input in this workflow is a list of genes co‐expressed with a query transcription factor (here, At*ARF7*‐At*ARF19*) identified using CORNET online database. The upstream elements of the co‐expressed genes are scanned for the presence of AuxRE motifs and such genes are used for building the network map using BiNGO plugin of Cytoscape. Genes with AuxREs and exhibiting co‐expression with query ARFs are assumed as putative downstream target genes of query ARFs and suggestively drive/participate in the transcriptomic signaling network. The network map is based on gene ontology, hence the functional roles of gene clusters are easily identifiable from the map.

**Figure S7** **Gene‐ontology based network map comprised of At*ARF7*‐ At*ARF19* co‐expressed and AuxRE motif possessing genes**. Such genes were assumed to be downstream target (transcriptomic) genes of At*ARF7‐*At*ARF19* transcription factors. Each bubble/circle represent a ‘biological process’, the size represents the number of genes comprising the bubble and the shading represent the significance of this clustering based on correlation p‐values.

**Table S1** List of Primers and Bacterial strains used

**Table S2** Accession Numbers of mRNA sequences utilized for designing Real‐time primers for *Nicotiana tabacum* *ARF*s.

**Table S3** Results of Data mining of Gene ontology file obtained from TAIR (www.arabidopsis.org) – A) Root‐related genes; B) transcription factor genes; C) root‐ related transcription factor genes and D) description of members of gene tree constructed (n = 102).

**Table S4** Co‐expressed genes of A) *ARF7‐ARF19*; B) *ARF5* and C) *ARF6‐ ARF8* as obtained from CORNET online database.

**Table S5** Results of AuxRE motif scanning by FIMO (of MEME Suite) on upstream sequences (3,000 bp) of co‐expressed genes of A) *ARF7‐ARF19*; B) *ARF5* and C) *ARF6‐ARF8* (highlighted cells).

**Table S6** Functional categories represented by AuxRE motif possessing co‐expressed genes of A) *ARF7‐ARF19*; B) *ARF5* and C) *ARF6‐ARF8* analyzed by BiNGO.
