# Supplementary figures and images for: The rolB‐transgenic Nicotiana tabacum plants exhibit upregulated ARF7 and ARF19 gene expression
Source: Plant Direct. 2022 Jun 18;6(6):e414. doi: 10.1002/pld3.414 (PMC9219009; doi:10.1002/pld3.414)

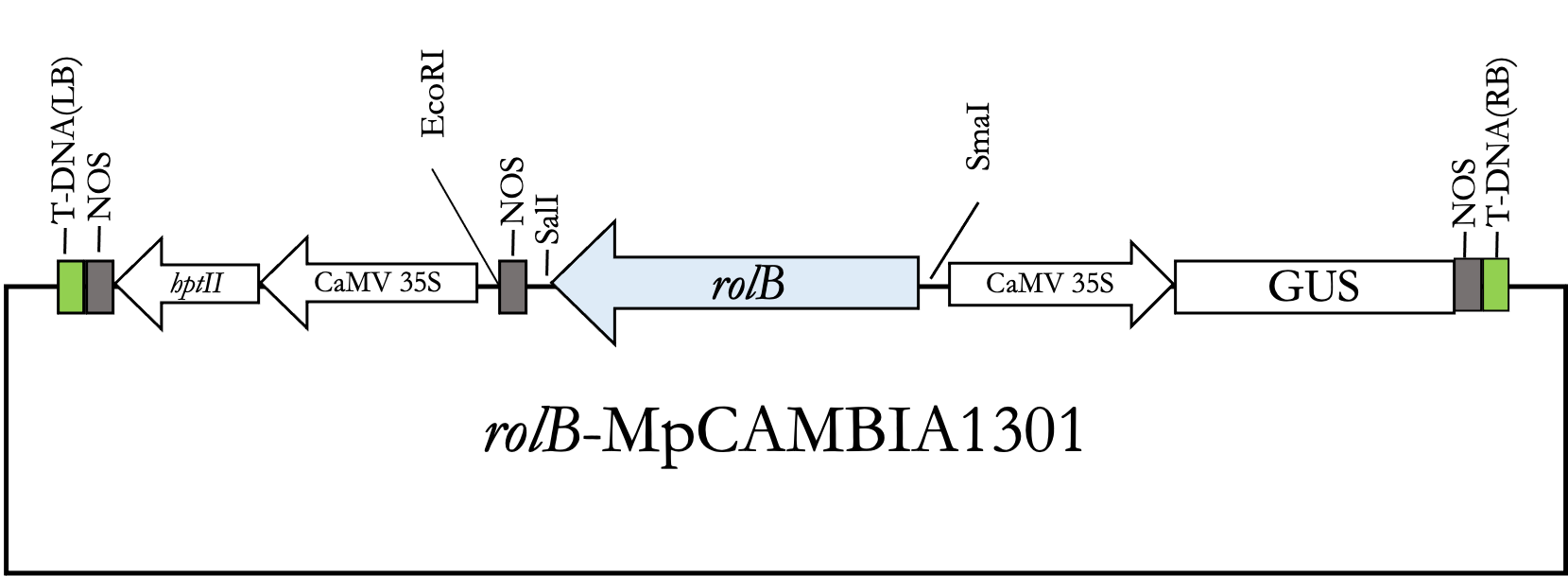

Supplement: Supplementary file 1 — Figure S1 Recombinant rolB transgene cassette constructed within MpCAMBIA1301 vector: A 2019 bp fragment harboring rolB TL was incorporated between SmaI and SalI restriction enzyme sites within the MpCAMBIA1301 vector. [file PLD3-6-e414-s005.png]

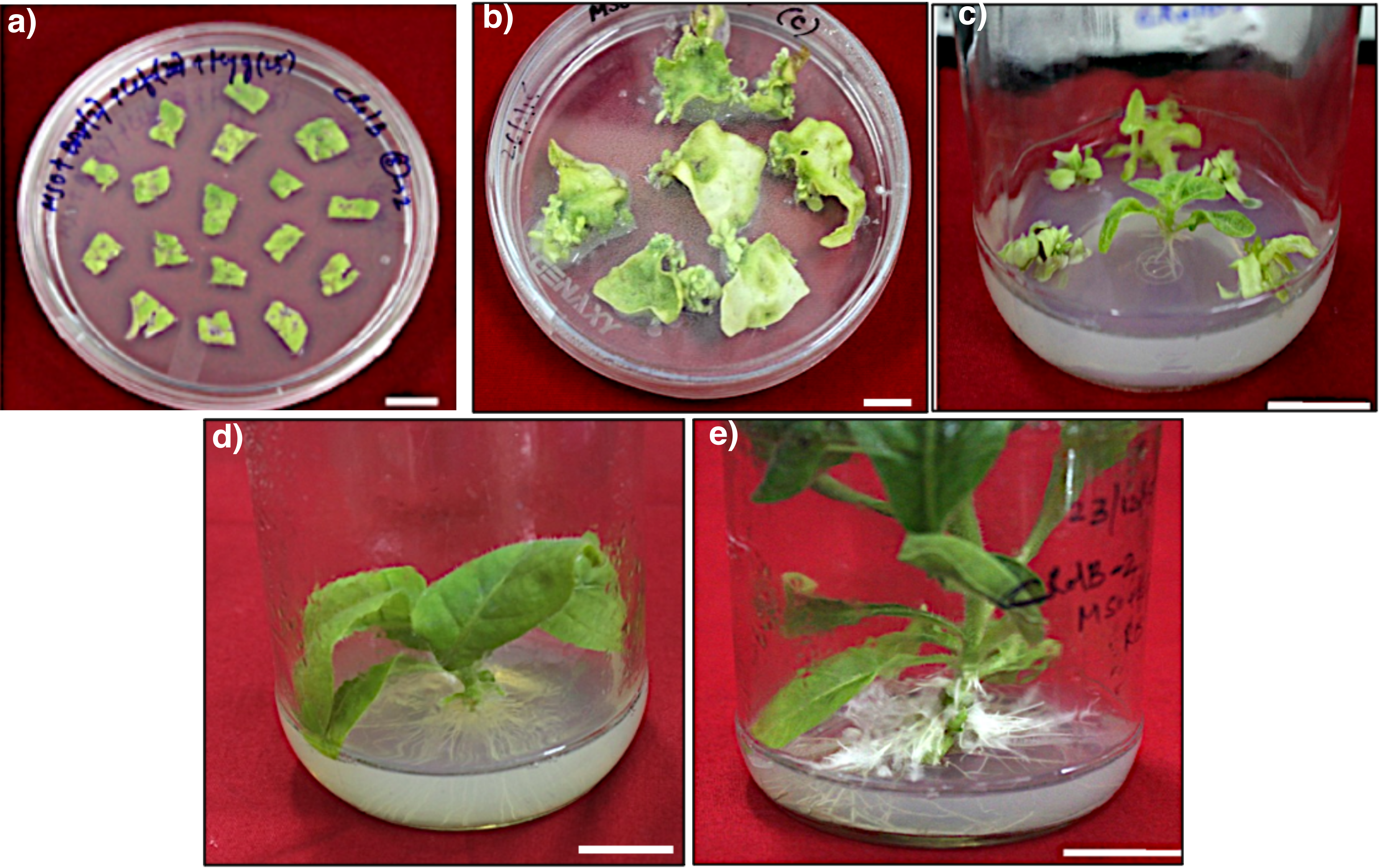

Supplement: Supplementary file 2 — Figure S2 Regeneration and maintenance of rolB transgenic plants (T0) in vitro ‐ a) Axenic leaf explants ( Nicotiana tabacum ) after co‐cultivation with Agrobacterium tumefaciens ‐rolB‐MpCAMBIA1301 on shoot regeneration medium (MS + BAP2 + Cef500 + Hyg25); b) Shoot bud induction on shoot regeneration medium (MS + BAP2 + Cef500 + Hyg25); c) Microshoots on MS without phytohormones but with Cef500 + Hyg25; d) and e) –rolB‐transgenic plant line (cRolB2) showing profuse root growth. The subscripts indicate antibiotic concentration in mg l−1. Abbr. ‐ Cef – Cefotaxime, Hyg – Hygromycin, MS medium – Murashige‐Skooge, BAP – Benzylaminopurine. White bar is scale – 2 cms. [file PLD3-6-e414-s002.png]

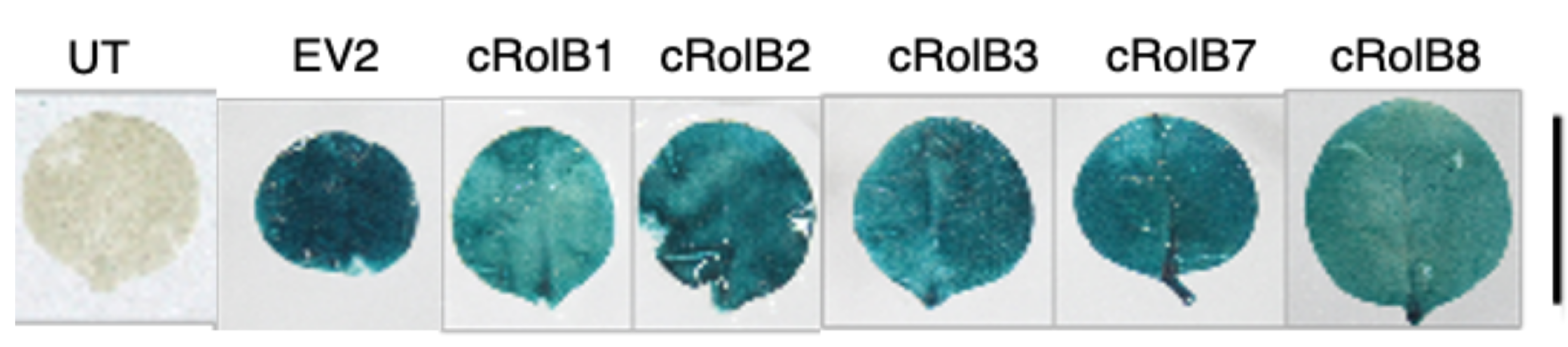

Supplement: Supplementary file 3 — Figure S3 Histochemical GUS staining of leaves of rolB‐transgenic T 2 and vector control T 2 seedlings confirming the presence of transgene cassette. From left to right – Leaves of plant line UT (Untransformed/wildtype), EV2 (vector control 2), rolB‐transgenic plant lines cRolB1, cRolB2, cRolB3, cRolB7, cRolB8 (rolB transgenic plants, bar = 1 cm). [file PLD3-6-e414-s001.png]

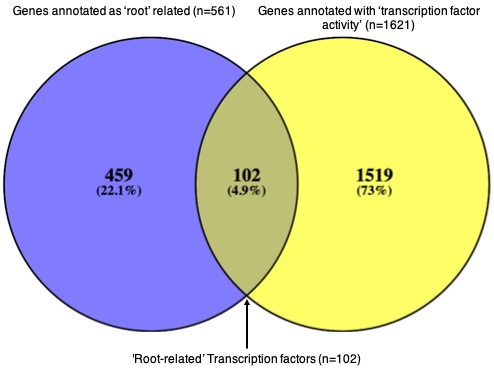

Supplement: Supplementary file 4 — Figure S4 Results of the datamining performed on the gene ontology record file obtained from TAIR website represented as a Venn diagram ‐ Root‐related transcription factors (n = 102) were found by the intersection of two sets. [file PLD3-6-e414-s004.zip › pld3_414-sup-0004-SupplementaryFigureS4.png]

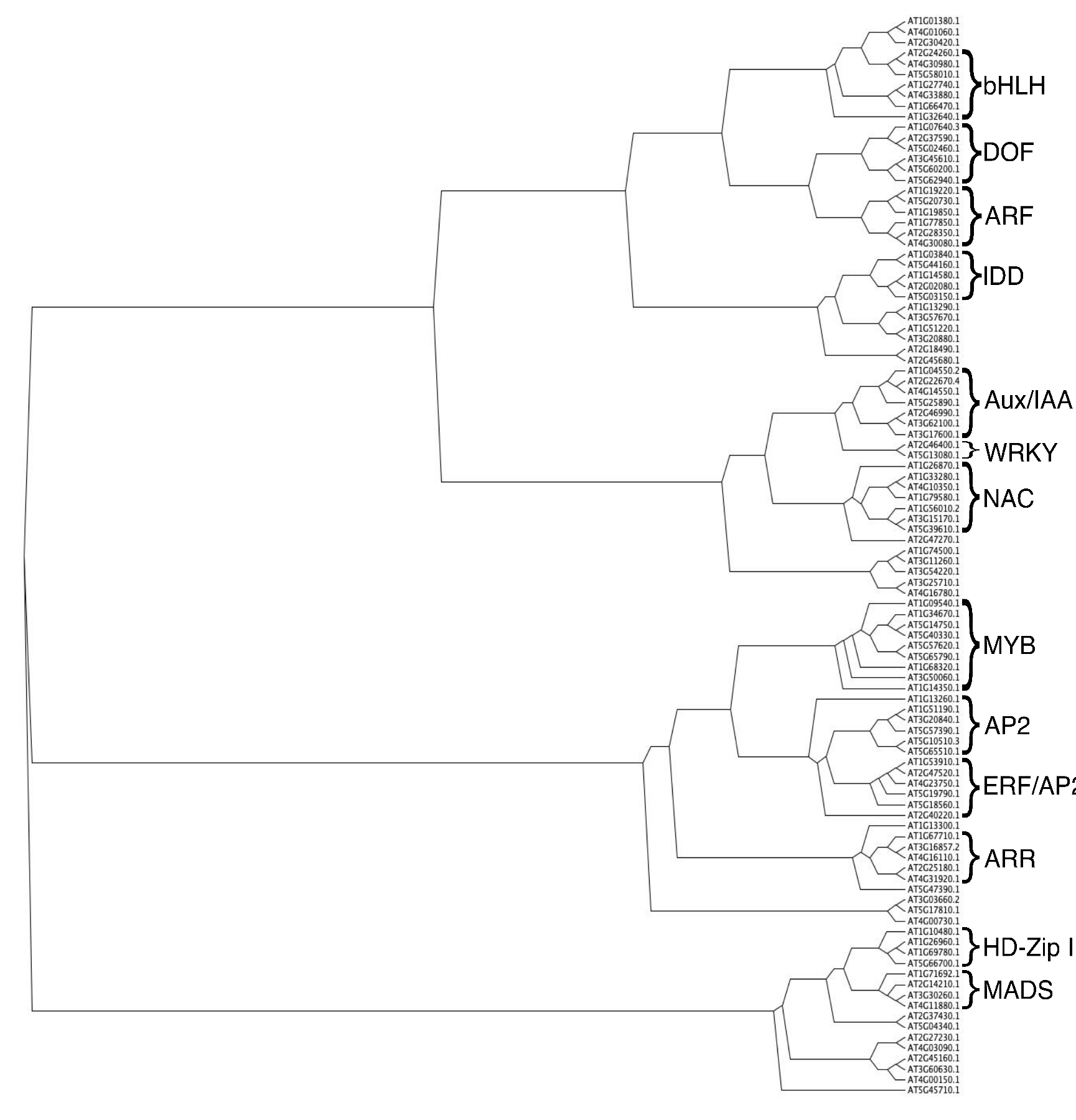

Supplement: Supplementary file 5 — Figure S5 Clustering of transcription factors into broad classes of protein families via tree building (Neighbor Joining method) using protein sequences (n = 102) corresponding to genes identified from datamining of gene ontology file ‐ The Auxin Response Transcription factor group (ARF) was identified as the third group in the tree (from top). [file PLD3-6-e414-s010.png]

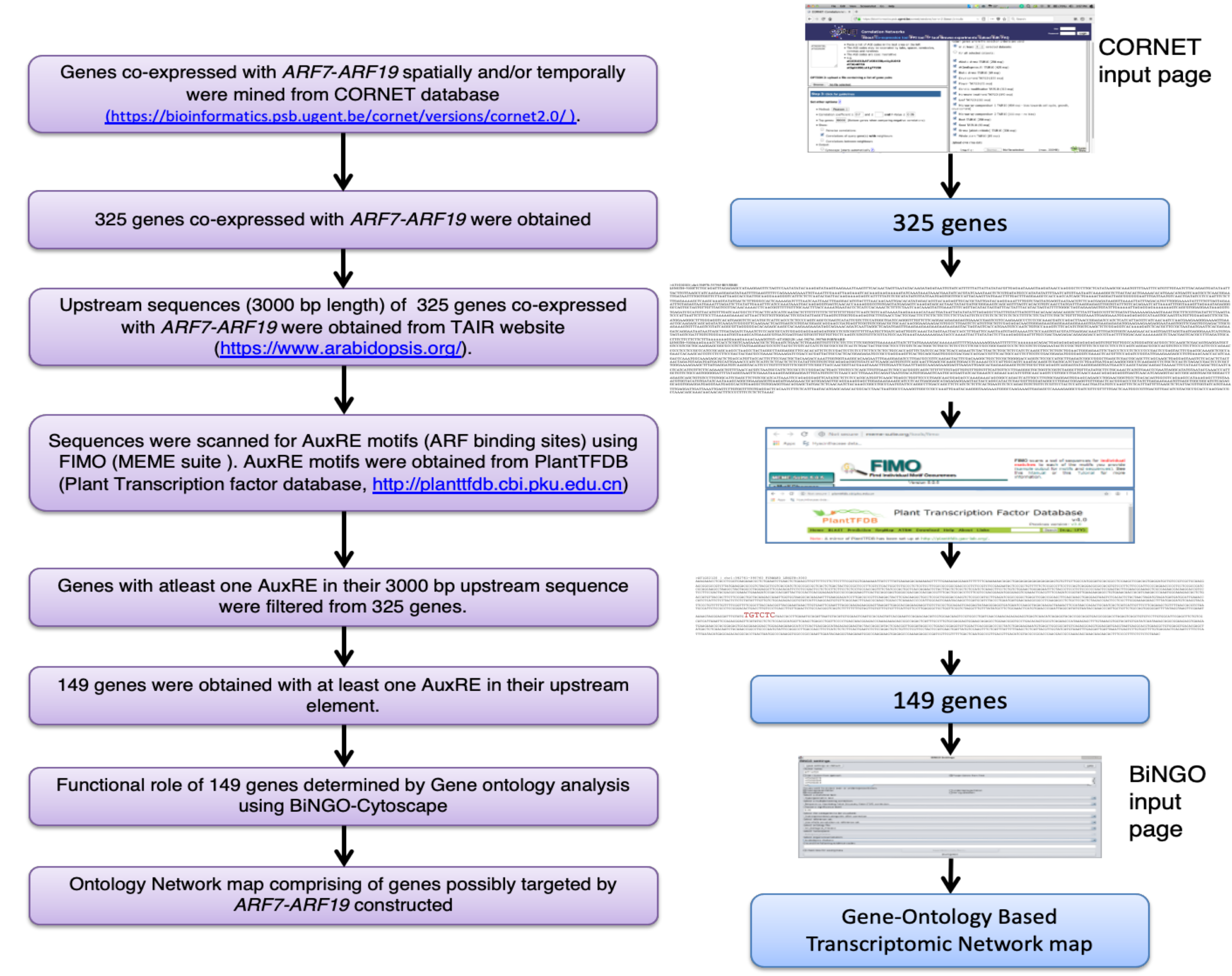

Supplement: Supplementary file 6 — Figure S6 Schematic representation of the workflow adopted to propose an ARF driven transcriptomic signaling network ‐ The initial input in this workflow is a list of genes co‐expressed with a query transcription factor (here, AtARF7‐AtARF19) identified using CORNET online database. The upstream elements of the co‐expressed genes are scanned for the presence of AuxRE motifs and such genes are used for building the network map using BiNGO plugin of Cytoscape. Genes with AuxREs and exhibiting co‐expression with query ARFs are assumed as putative downstream target genes of query ARFs and suggestively drive/participate in the transcriptomic signaling network. The network map is based on gene ontology, hence the functional roles of gene clusters are easily identifiable from the map. [file PLD3-6-e414-s007.png]

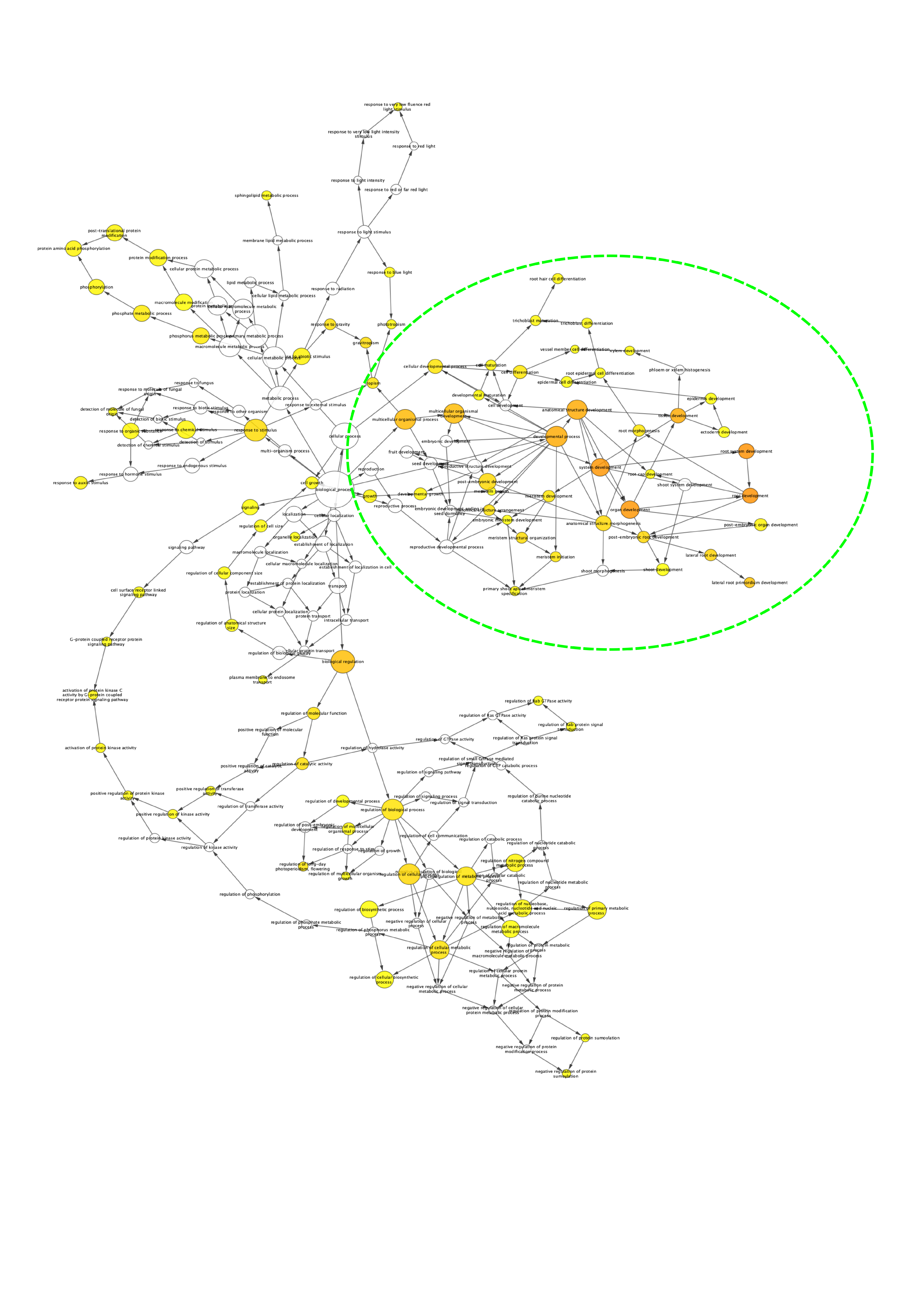

Supplement: Supplementary file 7 — Figure S7 Gene‐ontology based network map comprised of AtARF7‐ AtARF19 co‐expressed and AuxRE motif possessing genes. Such genes were assumed to be downstream target (transcriptomic) genes of AtARF7‐AtARF19 transcription factors. Each bubble/circle represent a ‘biological process’, the size represents the number of genes comprising the bubble and the shading represent the significance of this clustering based on correlation p‐values. [file PLD3-6-e414-s006.png]
