# Supplementary material for: The rolB‐transgenic Nicotiana tabacum plants exhibit upregulated ARF7 and ARF19 gene expression
Source: Plant Direct. 2022 Jun 18;6(6):e414. doi: 10.1002/pld3.414 (PMC9219009; doi:10.1002/pld3.414)
Supplement: Supplementary file 8 — Table S1 List of Primers and Bacterial strains used [file PLD3-6-e414-s011.pdf]

Supplementary Table S1 – List of Primers and Bacterial Strains used

Primer list

rolB Forward Primer (Cloning) – 5'- ATCCCCCGGGCTGCAGCTGCTTGTCACATCCTCCACT-3'
rolB Forward Primer (PCR) – 5'- ATGCTCTAGAATGGATCCCAAATTGCTATTC – 3'
rolB Reverse Primer (Cloning, PCR) – 5'-ACGCGTCGACTTAGGCTTCTTTCTTCAGGTT -3'

hptII Forward Primer (PCR) – 5'- GATGTTGGCGACCTCGTATT-3'
hptII Reverse Primer (PCR) – 5'- GAGTTTAGCGAGAGCCTGACCTAT-3'

rolB Forward Primer (RT-PCR) – 5'- GCCGTGACTATAGCAAACCCCTC - 3'
rolB Reverse Primer (RT-PCR) – 5'- ACGCCCTCCTCGCCTTCCT – 3'

L25 Forward Primer (RT-PCR, Real Time) – 5'- TGCACCTGGAAGGAACAAACTT – 3'
L25 Reverse Primer (RT-PCR, Real Time) – 5'- AAGGGTGTTGTTGTCCTCAATCTT -3'

Nt*ARF5* Forward Primer (Real-Time) – 5'- GGGTGGAAGCTGGTATATGTGGA -3'
Nt*ARF5* Reverse Primer (Real-Time) – 5'- CTTGCTGCACTTCTGTAGGCGATA-3'

Nt*ARF6* Forward Primer (Real-Time) – 5'-TCCTTATGCTACCTCTACCTTCACAAA-3'
Nt*ARF6* Reverse Primer (Real-Time) – 5'-CTCCAACAAGCCTTCTAGCCCA-3'

Nt*ARF17* Forward Primer (Real-Time) - 5'- AAGTGGCCCTTGGCGTGG – 3'
Nt*ARF17* Reverse Primer (Real-Time) - 5'- CGAACTTTTGTCAAGGAGCTGGAA -3'

Nt*ARF8* Forward Primer (Real-Time) – 5'- CCGAGGATGTGCAGAAACTGGG - 3'
Nt*ARF8* Reverse Primer (Real-Time) – 5'-GGCAGTCCAGACATGAAGTCTCGA – 3'

Nt*ARF10* Forward Primer (Real-Time) – 5'- GATGATGATAACATTTTGGGGGGTAG - 3'
Nt*ARF10* Reverse Primer (Real-Time) – 5'- CGTAGAAAATCCACCATAGTTTCCG – 3'

Nt*ARF16* Forward Primer (Real-Time) – 5'-AAAATGACACTGTTTTAGGAAGCAG- 3'
Nt*ARF16* Reverse Primer (Real-Time) – 5'- CCACCAGAAGAATTCAGTTCCAT – 3'

Nt*ARF7* Forward Primer (Real-Time)-5'- GCAGCCACAGCAACACTCGC -3'
Nt*ARF7* Reverse Primer (Real Time)-5'- TTGCTGCTGGGGTCCACGTT

Nt*ARF19* Forward Primer (Real Time)-5'- ACAACCACAACCACAACCACAGC -3'
Nt*ARF19* Reverse Primer (Real Time) -5'- TTGCTGCTGGGGTCCACGTT -3'

QPT2 Forward Primer – 5'- ACAAGAGTGGAGTCATTAGAGGTG -3'
QPT2 Reverse Primer – 5'- GCGAATATCATCTCAGCAAGTGC -3'

Bacterial Strains

1. **pBluescript II SK+** : Cloning vector with ampicillin resistance gene for selection of positive transformants and a truncated ß-galactosidase (lacZ) gene for Blue-White based screening of true recombinants (by principle of alpha complementation).
2. ***rolB*-pBluescript II SK+** : *rolB* cloned within pBluescript II SK+.
3. **MpCAMBIA1301** : A pCAMBIA1301 derivative/modified (Mukherjee et al 2019) with extra MCS sites within the transgene cassette along with hygromycin resistance gene (for

selection in plant system) and GUS gene (for GUS staining). The vector backbone also possesses a kanamycin resistance gene (for selection in bacteria).

4. ***rolB*-MpCAMBIA1301** : *rolB* cloned within MpCAMBIA1301.
