# Supplementary material for: The rolB‐transgenic Nicotiana tabacum plants exhibit upregulated ARF7 and ARF19 gene expression
Source: Plant Direct. 2022 Jun 18;6(6):e414. doi: 10.1002/pld3.414 (PMC9219009; doi:10.1002/pld3.414)
Supplement: Supplementary file 9 — Table S2 Accession Numbers of mRNA sequences utilized for designing Real‐time primers for Nicotiana tabacum ARFs. [file PLD3-6-e414-s008.pdf]

Supplementary Table S2 – Accession Numbers of mRNA sequences utilized for designing Realtime primers for *Nicotiana tabacum* ARFs.

NtARF7

S.No.	Accession Number	Source	Website Link
1	canonical mRNA for AT5G020730	TAIR	www.arabidopsis.org
2	mRNA_122225_cds	Sol Genomics	www.solgenomics.net
3	mRNA_138033_cds	Sol Genomics	www.solgenomics.net
4	mRNA_139956_cds	Sol Genomics	www.solgenomics.net
5	mRNA_139955_cds	Sol Genomics	www.solgenomics.net

NtARF19

S.No.	Accession Number	Source	Website Link
1	canonical mRNA for AT1G19220	TAIR	www.arabidopsis.org
2	mRNA_49921_cds	Sol Genomics	www.solgenomics.net
3	mRNA_58268_cds	Sol Genomics	www.solgenomics.net
4	mRNA_58267_cds	Sol Genomics	www.solgenomics.net
5	mRNA_57990_cds	Sol Genomics	www.solgenomics.net

NtARF10

S.No.	Accession Number	Source	Website Link
1	HM143941.1	Sol Genomics	www.solgenomics.net
2	mRNA_125123	Sol Genomics	www.solgenomics.net
3	mRNA_125122	Sol Genomics	www.solgenomics.net
4	mRNA_125121	Sol Genomics	www.solgenomics.net
5	mRNA_125120	Sol Genomics	www.solgenomics.net
6	mRNA_125124	Sol Genomics	www.solgenomics.net
7	mRNA_119154	Sol Genomics	www.solgenomics.net
8	mRNA_119153	Sol Genomics	www.solgenomics.net
9	mRNA_119152	Sol Genomics	www.solgenomics.net

10	mRNA_119899	Sol Genomics	www.solgenomics.net
11	mRNA_119898	Sol Genomics	www.solgenomics.net
12	mRNA_119896	Sol Genomics	www.solgenomics.net
13	mRNA_119895	Sol Genomics	www.solgenomics.net
14	mRNA_119894	Sol Genomics	www.solgenomics.net

NtARF16

S.No.	Accession Number	Source	Website Link
1	HM195247.1	Sol Genomics	www.solgenomics.net
2	mRNA_59044	Sol Genomics	www.solgenomics.net
3	mRNA_59043	Sol Genomics	www.solgenomics.net
4	mRNA_59042	Sol Genomics	www.solgenomics.net
5	mRNA_59041	Sol Genomics	www.solgenomics.net
6	mRNA_67198	Sol Genomics	www.solgenomics.net
7	mRNA_67199	Sol Genomics	www.solgenomics.net
8	mRNA_67200	Sol Genomics	www.solgenomics.net
9	mRNA_67201	Sol Genomics	www.solgenomics.net
10	mRNA_67850	Sol Genomics	www.solgenomics.net
11	mRNA_67851	Sol Genomics	www.solgenomics.net
12	mRNA_67852	Sol Genomics	www.solgenomics.net
13	mRNA_67853	Sol Genomics	www.solgenomics.net
14	mRNA_67854	Sol Genomics	www.solgenomics.net
15	mRNA_67855	Sol Genomics	www.solgenomics.net

NtARF6

S.No.	Accession Number	Source	Website Link
1	mRNA_128076_cds	Sol Genomics	www.solgenomics.net
2	mRNA_136896_cds	Sol Genomics	www.solgenomics.net
3	mRNA_136895_cds	Sol Genomics	www.solgenomics.net
4	mRNA_134032_cds	Sol Genomics	www.solgenomics.net
5	mRNA_134030_cds	Sol Genomics	www.solgenomics.net
6	mRNA_134029_cds	Sol Genomics	www.solgenomics.net

7	canonical mRNA for AT1G30330	TAIR	www.arabidopsis.org

NtARF5

S.No.	Accession Number	Source	Website Link
1	mRNA_81660_cds	Sol Genomics	www.solgenomics.net
2	mRNA_81661_cds	Sol Genomics	www.solgenomics.net
3	mRNA_68283_cds	Sol Genomics	www.solgenomics.net
4	mRNA_68284_cds	Sol Genomics	www.solgenomics.net
5	mRNA_68285_cds	Sol Genomics	www.solgenomics.net
6	mRNA_68286_cds	Sol Genomics	www.solgenomics.net
7	canonical mRNA for AT1G19850	TAIR	www.arabidopsis.org

NtARF8

S.No.	Accession Number	Source	Website Link
1	mRNA_86029_cds	Sol Genomics	www.solgenomics.net
2	mRNA_86030_cds	Sol Genomics	www.solgenomics.net
3	mRNA_86031_cds	Sol Genomics	www.solgenomics.net
4	mRNA_85540_cds	Sol Genomics	www.solgenomics.net
5	mRNA_109567_cds	Sol Genomics	www.solgenomics.net
6	mRNA_110565_cds	Sol Genomics	www.solgenomics.net
7	mRNA_110566_cds	Sol Genomics	www.solgenomics.net
8	mRNA_110567_cds	Sol Genomics	www.solgenomics.net
9	mRNA_73064_cds	Sol Genomics	www.solgenomics.net
10	mRNA_73065_cds	Sol Genomics	www.solgenomics.net
11	mRNA_73066_cds	Sol Genomics	www.solgenomics.net
12	canonical mRNA for AT5G37020	TAIR	www.arabidopsis.org

NtARF17

S.No.	Accession Number	Source	Website Link
1	mRNA_134477_cds	Sol Genomics	www.solgenomics.net
2	mRNA_143247_cds	Sol Genomics	www.solgenomics.net

3	mRNA_140593_cds	Sol Genomics	www.solgenomics.net
4	mRNA_123921_cds	Sol Genomics	www.solgenomics.net
5	mRNA_121927_cds	Sol Genomics	www.solgenomics.net
6	mRNA_134645_cds	Sol Genomics	www.solgenomics.net
7	mRNA_134644_cds	Sol Genomics	www.solgenomics.net
8	mRNA_134643_cds	Sol Genomics	www.solgenomics.net
9	mRNA_123920_cds	Sol Genomics	www.solgenomics.net
10	mRNA_123919_cds	Sol Genomics	www.solgenomics.net
11	mRNA_121926_cds	Sol Genomics	www.solgenomics.net
12	canonical mRNA for AT1G77850	Sol Genomics	www.solgenomics.net
