# Supplementary material for: The rolB‐transgenic Nicotiana tabacum plants exhibit upregulated ARF7 and ARF19 gene expression
Source: Plant Direct. 2022 Jun 18;6(6):e414. doi: 10.1002/pld3.414 (PMC9219009; doi:10.1002/pld3.414)
Supplement: Supplementary file 10 — Table S3 Results of Data mining of Gene ontology file obtained from TAIR (www.arabidopsis.org) – A) Root‐related genes; B) transcription factor genes; C) root‐ related transcription factor genes and D) description of members of gene tree constructed (n = 102). [file PLD3-6-e414-s013.pdf]

Supplementary Table S3 – Results of Data mining of Gene ontology file obtained from TAIR (www.arabidopsis.org) - A) Root-related genes

ADD2	AT1G10940	AT1G21410	AT1G48630	AT1G67490	AT1G79950	AT2G22670	AT2G37590	AT3G04630
ADD3	AT1G11130	AT1G23080	AT1G48920	AT1G67710	AT1G80600	AT2G22680	AT2G37620	AT3G05010
AMOS2	AT1G11760	AT1G23320	AT1G49240	AT1G68320	AT2G01420	AT2G22840	AT2G38120	AT3G05040
AT1G01335	AT1G12110	AT1G24460	AT1G49430	AT1G68765	AT2G01830	AT2G23096	AT2G39800	AT3G05090
AT1G01380	AT1G12240	AT1G24620	AT1G50460	AT1G69380	AT2G02080	AT2G23140	AT2G39830	AT3G05580
AT1G01550	AT1G12430	AT1G25220	AT1G51190	AT1G69780	AT2G02470	AT2G23430	AT2G39940	AT3G05630
AT1G01910	AT1G12560	AT1G26870	AT1G51220	AT1G70560	AT2G03720	AT2G23440	AT2G40220	AT3G05930
AT1G01950	AT1G12820	AT1G26960	AT1G51340	AT1G70940	AT2G03840	AT2G23460	AT2G41970	AT3G06300
AT1G02330	AT1G12950	AT1G27740	AT1G51590	AT1G71090	AT2G04025	AT2G24260	AT2G42430	AT3G06440
AT1G02335	AT1G13260	AT1G28560	AT1G52240	AT1G71410	AT2G04160	AT2G25010	AT2G42490	AT3G06810
AT1G02410	AT1G13290	AT1G30000	AT1G53330	AT1G71692	AT2G05520	AT2G25170	AT2G42500	AT3G07360
AT1G02900	AT1G13300	AT1G31880	AT1G53700	AT1G71696	AT2G10606	AT2G25180	AT2G42590	AT3G07390
AT1G03840	AT1G13620	AT1G31930	AT1G53910	AT1G72180	AT2G11890	AT2G26420	AT2G43790	AT3G07780
AT1G04160	AT1G13870	AT1G32080	AT1G55020	AT1G72490	AT2G14210	AT2G26650	AT2G44900	AT3G07880
AT1G04550	AT1G13980	AT1G32230	AT1G55180	AT1G73590	AT2G16385	AT2G26870	AT2G45160	AT3G08550
AT1G05630	AT1G14350	AT1G32640	AT1G56010	AT1G74380	AT2G17500	AT2G27230	AT2G45420	AT3G08800
AT1G05850	AT1G14580	AT1G33280	AT1G56560	AT1G74500	AT2G17720	AT2G28350	AT2G45680	AT3G09070
AT1G06230	AT1G15570	AT1G34110	AT1G58340	AT1G74560	AT2G17780	AT2G30330	AT2G45890	AT3G11260
AT1G07640	AT1G15700	AT1G34670	AT1G59610	AT1G74800	AT2G18470	AT2G30340	AT2G46020	AT3G11600
AT1G07880	AT1G16300	AT1G35580	AT1G59820	AT1G75110	AT2G18480	AT2G30420	AT2G46400	AT3G12070
AT1G08030	AT1G16510	AT1G35720	AT1G59835	AT1G75120	AT2G18490	AT2G31650	AT2G46990	AT3G12380
AT1G08090	AT1G16890	AT1G36160	AT1G60370	AT1G77000	AT2G18800	AT2G33790	AT2G47000	AT3G13300
AT1G08100	AT1G17110	AT1G44900	AT1G60940	AT1G77110	AT2G19560	AT2G34650	AT2G47270	AT3G13870
AT1G08260	AT1G18420	AT1G47200	AT1G62830	AT1G77690	AT2G19760	AT2G34680	AT2G47520	AT3G14370
AT1G08300	AT1G18640	AT1G47240	AT1G63450	AT1G77740	AT2G20000	AT2G35510	AT3G01090	AT3G15150
AT1G09540	AT1G18800	AT1G47485	AT1G64440	AT1G77850	AT2G20140	AT2G35610	AT3G01730	AT3G15170
AT1G09560	AT1G19220	AT1G48380	AT1G64520	AT1G78240	AT2G20610	AT2G35612	AT3G02242	AT3G15380
AT1G10130	AT1G19360	AT1G48410	AT1G65570	AT1G78870	AT2G21050	AT2G36400	AT3G02260	AT3G16640
AT1G10370	AT1G19850	AT1G48490	AT1G66470	AT1G79530	AT2G21170	AT2G37250	AT3G03660	AT3G16785
AT1G10480	AT1G21210	AT1G48550	AT1G66700	AT1G79580	AT2G22475	AT2G37430	AT3G03773	AT3G16857

AT3G17600	AT3G29030	AT3G60650	AT4G14550	AT4G30160	AT5G03280	AT5G16910	AT5G44160	AT5G55920
AT3G17850	AT3G30260	AT3G61440	AT4G14850	AT4G30340	AT5G03540	AT5G17400	AT5G44280	AT5G56040
AT3G18000	AT3G43960	AT3G61650	AT4G15290	AT4G30980	AT5G03730	AT5G17810	AT5G44610	AT5G56320
AT3G18130	AT3G44600	AT3G62020	AT4G15900	AT4G31170	AT5G04340	AT5G18520	AT5G44700	AT5G56540
AT3G18485	AT3G44750	AT3G62100	AT4G16110	AT4G31230	AT5G05500	AT5G18560	AT5G45420	AT5G56580
AT3G18780	AT3G45610	AT3G62270	AT4G16444	AT4G31400	AT5G05620	AT5G19700	AT5G45550	AT5G56600
AT3G18830	AT3G48610	AT3G62720	AT4G16515	AT4G31500	AT5G05730	AT5G19790	AT5G45710	AT5G57280
AT3G19770	AT3G50060	AT3G62980	AT4G16780	AT4G31600	AT5G05980	AT5G20490	AT5G46700	AT5G57390
AT3G20630	AT3G50310	AT3G63420	AT4G18640	AT4G31920	AT5G06140	AT5G20520	AT5G47040	AT5G57620
AT3G20840	AT3G51050	AT4G00150	AT4G18710	AT4G33495	AT5G06270	AT5G20730	AT5G47390	AT5G57740
AT3G20860	AT3G51460	AT4G00730	AT4G20140	AT4G33880	AT5G09350	AT5G22650	AT5G47990	AT5G58010
AT3G20880	AT3G51550	AT4G01060	AT4G21060	AT4G34390	AT5G09680	AT5G24310	AT5G48000	AT5G58270
AT3G21160	AT3G51770	AT4G01630	AT4G23750	AT4G34460	AT5G09810	AT5G24630	AT5G48010	AT5G58710
AT3G21220	AT3G53180	AT4G02450	AT4G24400	AT4G34580	AT5G10250	AT5G24740	AT5G48160	AT5G59030
AT3G22200	AT3G53480	AT4G02500	AT4G24560	AT4G34600	AT5G10510	AT5G25460	AT5G48940	AT5G60200
AT3G22400	AT3G54010	AT4G03090	AT4G24670	AT4G35910	AT5G10720	AT5G25890	AT5G49270	AT5G60660
AT3G22650	AT3G54220	AT4G04920	AT4G25250	AT4G37060	AT5G11030	AT5G26700	AT5G49660	AT5G60810
AT3G22942	AT3G54610	AT4G05530	AT4G26270	AT4G37070	AT5G11630	AT5G26930	AT5G49665	AT5G61070
AT3G23940	AT3G54720	AT4G07410	AT4G26540	AT4G37295	AT5G12210	AT5G27840	AT5G49680	AT5G61350
AT3G24225	AT3G54870	AT4G08480	AT4G26690	AT4G37740	AT5G12235	AT5G28640	AT5G51040	AT5G61980
AT3G24240	AT3G56370	AT4G08500	AT4G26760	AT4G38630	AT5G12330	AT5G35407	AT5G51060	AT5G62940
AT3G24300	AT3G56930	AT4G08920	AT4G28460	AT4G39403	AT5G13010	AT5G39610	AT5G51310	AT5G63220
AT3G25520	AT3G57630	AT4G09510	AT4G28490	AT4G39990	AT5G13080	AT5G40330	AT5G51451	AT5G63770
AT3G25710	AT3G57670	AT4G10350	AT4G28980	AT5G01170	AT5G13290	AT5G40770	AT5G52050	AT5G64070
AT3G25820	AT3G58060	AT4G11880	AT4G29040	AT5G01240	AT5G13750	AT5G41950	AT5G52310	AT5G64770
AT3G25830	AT3G58190	AT4G12550	AT4G29140	AT5G01490	AT5G14170	AT5G42080	AT5G55230	AT5G65020
AT3G26540	AT3G59420	AT4G13510	AT4G29350	AT5G02460	AT5G14750	AT5G42810	AT5G55580	AT5G65090
AT3G27560	AT3G60350	AT4G13750	AT4G29860	AT5G02600	AT5G16780	AT5G43070	AT5G55730	AT5G65510
AT3G28860	AT3G60630	AT4G14430	AT4G30080	AT5G03150	AT5G16850	AT5G43900	AT5G55910	AT5G65683

AT5G65710
AT5G65790
AT5G66700
AT5G66815
AT5G66816
CEN1
CEN2
CEN3
CUD
DER2
DER5
DER6
DER7
DER8
DHM1
ENL7
ERH1
LIT
LPI1
LPI2
LPI3
LPI4
MGO2
MOP2
MOP3
RIB1
RM57
RML2
SHV1
SRB

Supplementary Table S3 – Results of Data mining of Gene ontology file obtained from TAIR (www.arabidopsis.org) - B) Transcription factor genes

AT1G01010	AT1G05710	AT1G10586	AT1G17520	AT1G21450	AT1G26790	AT1G30670	AT1G34790	AT1G49560
AT1G01030	AT1G05805	AT1G10610	AT1G17590	AT1G21910	AT1G26870	AT1G30970	AT1G35240	AT1G49720
AT1G01060	AT1G06040	AT1G11490	AT1G17920	AT1G21970	AT1G26960	AT1G31050	AT1G35460	AT1G49770
AT1G01250	AT1G06070	AT1G12260	AT1G17950	AT1G22070	AT1G27050	AT1G31140	AT1G35490	AT1G49830
AT1G01260	AT1G06150	AT1G12540	AT1G18330	AT1G22130	AT1G27360	AT1G31150	AT1G35515	AT1G49900
AT1G01380	AT1G06160	AT1G12610	AT1G18400	AT1G22190	AT1G27370	AT1G31310	AT1G35520	AT1G49950
AT1G01520	AT1G06170	AT1G12630	AT1G18570	AT1G22490	AT1G27660	AT1G31630	AT1G35540	AT1G50420
AT1G01530	AT1G06180	AT1G12860	AT1G18710	AT1G22590	AT1G27730	AT1G31640	AT1G35560	AT1G50600
AT1G01720	AT1G06850	AT1G12890	AT1G18750	AT1G22640	AT1G27740	AT1G32150	AT1G36060	AT1G50640
AT1G02030	AT1G07520	AT1G12980	AT1G18790	AT1G22810	AT1G28050	AT1G32240	AT1G42990	AT1G50680
AT1G02040	AT1G07530	AT1G13260	AT1G18860	AT1G22985	AT1G28160	AT1G32330	AT1G43160	AT1G50780
AT1G02220	AT1G07640	AT1G13290	AT1G18960	AT1G23380	AT1G28300	AT1G32360	AT1G43330	AT1G51070
AT1G02230	AT1G07980	AT1G13300	AT1G19000	AT1G23420	AT1G28310	AT1G32510	AT1G43700	AT1G51120
AT1G02250	AT1G08000	AT1G13450	AT1G19040	AT1G24260	AT1G28360	AT1G32640	AT1G43860	AT1G51140
AT1G02340	AT1G08010	AT1G13600	AT1G19210	AT1G24590	AT1G28370	AT1G32770	AT1G43950	AT1G51190
AT1G03040	AT1G08290	AT1G13960	AT1G19220	AT1G24625	AT1G28420	AT1G32870	AT1G44830	AT1G51220
AT1G03290	AT1G08320	AT1G14350	AT1G19350	AT1G25250	AT1G28450	AT1G33060	AT1G45249	AT1G51700
AT1G03490	AT1G08465	AT1G14490	AT1G19490	AT1G25310	AT1G28460	AT1G33070	AT1G46264	AT1G51950
AT1G03790	AT1G08540	AT1G14580	AT1G19700	AT1G25330	AT1G28470	AT1G33240	AT1G46408	AT1G52150
AT1G03800	AT1G08810	AT1G14600	AT1G19850	AT1G25340	AT1G29010	AT1G33280	AT1G46480	AT1G52830
AT1G03840	AT1G08970	AT1G14685	AT1G20640	AT1G25440	AT1G29160	AT1G33760	AT1G46768	AT1G52880
AT1G03970	AT1G09030	AT1G14920	AT1G20693	AT1G25470	AT1G29280	AT1G34170	AT1G47655	AT1G52890
AT1G04100	AT1G09250	AT1G15050	AT1G20696	AT1G25550	AT1G29860	AT1G34180	AT1G47760	AT1G53160
AT1G04240	AT1G09530	AT1G15360	AT1G20700	AT1G25560	AT1G29950	AT1G34190	AT1G47870	AT1G53170
AT1G04250	AT1G09540	AT1G15580	AT1G20710	AT1G25580	AT1G29962	AT1G34310	AT1G48000	AT1G53230
AT1G04370	AT1G09770	AT1G16060	AT1G20900	AT1G26260	AT1G30210	AT1G34370	AT1G48150	AT1G53320
AT1G04550	AT1G10120	AT1G16070	AT1G20910	AT1G26310	AT1G30330	AT1G34390	AT1G49010	AT1G53910
AT1G04880	AT1G10170	AT1G16490	AT1G20980	AT1G26590	AT1G30490	AT1G34410	AT1G49120	AT1G54060
AT1G05230	AT1G10480	AT1G17040	AT1G21200	AT1G26610	AT1G30500	AT1G34650	AT1G49130	AT1G54160
AT1G05290	AT1G10585	AT1G17310	AT1G21340	AT1G26780	AT1G30650	AT1G34670	AT1G49190	AT1G54330

AT1G54760	AT1G61940	AT1G66470	AT1G69310	AT1G73730	AT1G77200	AT2G01930	AT2G17180	AT2G22540
AT1G54830	AT1G62300	AT1G66550	AT1G69490	AT1G73805	AT1G77450	AT2G01940	AT2G17410	AT2G22630
AT1G55110	AT1G62360	AT1G66560	AT1G69540	AT1G73830	AT1G77570	AT2G02060	AT2G17560	AT2G22670
AT1G55520	AT1G62700	AT1G66600	AT1G69560	AT1G73870	AT1G77640	AT2G02070	AT2G17770	AT2G22750
AT1G55580	AT1G62970	AT1G67030	AT1G69570	AT1G74080	AT1G77850	AT2G02080	AT2G17950	AT2G22760
AT1G55600	AT1G62975	AT1G67260	AT1G69580	AT1G74430	AT1G77920	AT2G02450	AT2G18060	AT2G22770
AT1G55650	AT1G62990	AT1G67710	AT1G69600	AT1G74480	AT1G77950	AT2G02820	AT2G18160	AT2G22800
AT1G56010	AT1G63030	AT1G67970	AT1G69690	AT1G74500	AT1G77980	AT2G03060	AT2G18300	AT2G22850
AT1G56160	AT1G63100	AT1G68120	AT1G69780	AT1G74650	AT1G78080	AT2G03340	AT2G18380	AT2G23290
AT1G56170	AT1G63490	AT1G68130	AT1G69810	AT1G74840	AT1G78310	AT2G03500	AT2G18490	AT2G23320
AT1G56280	AT1G63650	AT1G68150	AT1G70000	AT1G74930	AT1G78540	AT2G03710	AT2G18550	AT2G23340
AT1G56650	AT1G63910	AT1G68190	AT1G70510	AT1G75080	AT1G78600	AT2G04038	AT2G18750	AT2G23740
AT1G57560	AT1G64000	AT1G68200	AT1G70920	AT1G75240	AT1G78700	AT2G04880	AT2G19810	AT2G23760
AT1G58100	AT1G64105	AT1G68240	AT1G71030	AT1G75250	AT1G79180	AT2G04890	AT2G20080	AT2G24260
AT1G59530	AT1G64380	AT1G68320	AT1G71130	AT1G75390	AT1G79430	AT2G06020	AT2G20100	AT2G24300
AT1G59640	AT1G64530	AT1G68360	AT1G71200	AT1G75410	AT1G79580	AT2G12900	AT2G20110	AT2G24340
AT1G59750	AT1G64620	AT1G68480	AT1G71450	AT1G75430	AT1G79700	AT2G12940	AT2G20180	AT2G24430
AT1G59810	AT1G64625	AT1G68520	AT1G71520	AT1G75490	AT1G79840	AT2G13150	AT2G20350	AT2G24500
AT1G60040	AT1G64800	AT1G68550	AT1G71692	AT1G75520	AT1G80390	AT2G13570	AT2G20400	AT2G24570
AT1G60240	AT1G64860	AT1G68640	AT1G71930	AT1G75540	AT1G80580	AT2G13960	AT2G20570	AT2G24790
AT1G60250	AT1G65300	AT1G68670	AT1G72010	AT1G75710	AT1G80590	AT2G14210	AT2G20880	AT2G24840
AT1G60280	AT1G65330	AT1G68800	AT1G72050	AT1G76110	AT1G80730	AT2G14760	AT2G21230	AT2G25000
AT1G60300	AT1G65360	AT1G68810	AT1G72210	AT1G76350	AT1G80840	AT2G15660	AT2G21235	AT2G25180
AT1G60340	AT1G65910	AT1G68840	AT1G72350	AT1G76420	AT2G01060	AT2G15740	AT2G21240	AT2G25230
AT1G60350	AT1G66140	AT1G68880	AT1G72360	AT1G76500	AT2G01200	AT2G16400	AT2G21320	AT2G25820
AT1G60380	AT1G66230	AT1G68920	AT1G72570	AT1G76510	AT2G01430	AT2G16720	AT2G21650	AT2G25900
AT1G60880	AT1G66350	AT1G69010	AT1G72740	AT1G76580	AT2G01500	AT2G16770	AT2G21900	AT2G25930
AT1G60920	AT1G66370	AT1G69120	AT1G72830	AT1G76880	AT2G01570	AT2G16910	AT2G22200	AT2G26150
AT1G61110	AT1G66380	AT1G69170	AT1G73360	AT1G76890	AT2G01710	AT2G17040	AT2G22300	AT2G26320
AT1G61660	AT1G66390	AT1G69180	AT1G73410	AT1G77080	AT2G01760	AT2G17150	AT2G22430	AT2G26580

AT2G26880	AT2G30470	AT2G34820	AT2G38880	AT2G42830	AT2G46770	AT3G03260	AT3G10040	AT3G13890
AT2G26940	AT2G30590	AT2G34830	AT2G39250	AT2G42940	AT2G46810	AT3G03450	AT3G10470	AT3G14020
AT2G26950	AT2G31070	AT2G35270	AT2G39880	AT2G43000	AT2G46830	AT3G03660	AT3G10480	AT3G14180
AT2G26960	AT2G31180	AT2G35430	AT2G40140	AT2G43010	AT2G46870	AT3G04030	AT3G10490	AT3G14230
AT2G27050	AT2G31210	AT2G35530	AT2G40200	AT2G43060	AT2G46970	AT3G04060	AT3G10500	AT3G15030
AT2G27070	AT2G31215	AT2G35550	AT2G40210	AT2G43140	AT2G46990	AT3G04070	AT3G10580	AT3G15170
AT2G27100	AT2G31220	AT2G35640	AT2G40220	AT2G43500	AT2G47070	AT3G04100	AT3G10590	AT3G15210
AT2G27220	AT2G31230	AT2G35670	AT2G40260	AT2G44730	AT2G47190	AT3G04410	AT3G10760	AT3G15270
AT2G27230	AT2G31280	AT2G35700	AT2G40340	AT2G44745	AT2G47260	AT3G04420	AT3G10800	AT3G15500
AT2G27300	AT2G31370	AT2G35940	AT2G40350	AT2G44840	AT2G47270	AT3G04430	AT3G11020	AT3G15510
AT2G27470	AT2G31380	AT2G36010	AT2G40620	AT2G44910	AT2G47460	AT3G04450	AT3G11100	AT3G15540
AT2G27990	AT2G31730	AT2G36080	AT2G40740	AT2G44940	AT2G47520	AT3G04570	AT3G11260	AT3G16160
AT2G28160	AT2G32080	AT2G36270	AT2G40750	AT2G45050	AT2G47810	AT3G04670	AT3G11280	AT3G16280
AT2G28200	AT2G32370	AT2G36450	AT2G40950	AT2G45120	AT2G47890	AT3G04730	AT3G11440	AT3G16350
AT2G28340	AT2G32460	AT2G36610	AT2G40970	AT2G45160	AT3G01030	AT3G04850	AT3G11580	AT3G16500
AT2G28350	AT2G33310	AT2G36890	AT2G41070	AT2G45190	AT3G01080	AT3G05690	AT3G12130	AT3G16770
AT2G28510	AT2G33480	AT2G36990	AT2G41130	AT2G45430	AT3G01140	AT3G05800	AT3G12250	AT3G16857
AT2G28550	AT2G33500	AT2G37000	AT2G41180	AT2G45650	AT3G01220	AT3G05860	AT3G12480	AT3G16870
AT2G28610	AT2G33510	AT2G37060	AT2G41240	AT2G45660	AT3G01330	AT3G06120	AT3G12510	AT3G17100
AT2G28700	AT2G33550	AT2G37260	AT2G41470	AT2G45680	AT3G01470	AT3G06490	AT3G12720	AT3G17600
AT2G28710	AT2G33710	AT2G37430	AT2G41690	AT2G45880	AT3G01530	AT3G06590	AT3G12730	AT3G17609
AT2G28810	AT2G33720	AT2G37590	AT2G41710	AT2G46130	AT3G01600	AT3G06740	AT3G12820	AT3G17730
AT2G29060	AT2G33810	AT2G37630	AT2G41900	AT2G46270	AT3G01970	AT3G07340	AT3G12910	AT3G18010
AT2G29065	AT2G33860	AT2G37650	AT2G41940	AT2G46310	AT3G02150	AT3G07650	AT3G13000	AT3G18100
AT2G29660	AT2G33880	AT2G37740	AT2G42200	AT2G46400	AT3G02310	AT3G08500	AT3G13040	AT3G18400
AT2G30250	AT2G34140	AT2G38090	AT2G42280	AT2G46410	AT3G02380	AT3G09230	AT3G13350	AT3G18550
AT2G30280	AT2G34440	AT2G38250	AT2G42300	AT2G46510	AT3G02790	AT3G09290	AT3G13445	AT3G18650
AT2G30420	AT2G34450	AT2G38300	AT2G42380	AT2G46530	AT3G02940	AT3G09370	AT3G13540	AT3G19070
AT2G30424	AT2G34710	AT2G38340	AT2G42410	AT2G46590	AT3G02990	AT3G09600	AT3G13810	AT3G19290
AT2G30432	AT2G34720	AT2G38470	AT2G42660	AT2G46680	AT3G03200	AT3G10000	AT3G13840	AT3G19360

AT3G19500	AT3G23250	AT3G30530	AT3G50260	AT3G55730	AT3G60580	AT4G00940	AT4G09460	AT4G16150
AT3G19510	AT3G23690	AT3G44290	AT3G50330	AT3G55980	AT3G60630	AT4G00950	AT4G09820	AT4G16430
AT3G19580	AT3G24050	AT3G44350	AT3G50410	AT3G56400	AT3G60870	AT4G01060	AT4G09960	AT4G16610
AT3G19860	AT3G24120	AT3G44460	AT3G50650	AT3G56520	AT3G61120	AT4G01120	AT4G10240	AT4G16750
AT3G20310	AT3G24140	AT3G45150	AT3G50700	AT3G56530	AT3G61150	AT4G01250	AT4G10350	AT4G16780
AT3G20640	AT3G24310	AT3G45170	AT3G50750	AT3G56560	AT3G61250	AT4G01280	AT4G11070	AT4G16845
AT3G20740	AT3G24490	AT3G45260	AT3G50870	AT3G56660	AT3G61630	AT4G01460	AT4G11080	AT4G17050
AT3G20770	AT3G24500	AT3G45610	AT3G51060	AT3G56710	AT3G61830	AT4G01500	AT4G11140	AT4G17230
AT3G20810	AT3G24520	AT3G46070	AT3G51080	AT3G56770	AT3G61850	AT4G01520	AT4G11250	AT4G17460
AT3G20840	AT3G24650	AT3G46080	AT3G51560	AT3G56850	AT3G61890	AT4G01540	AT4G11660	AT4G17490
AT3G20880	AT3G24860	AT3G46090	AT3G51880	AT3G56970	AT3G61910	AT4G01550	AT4G11880	AT4G17500
AT3G20910	AT3G25710	AT3G46130	AT3G51910	AT3G56980	AT3G61950	AT4G01680	AT4G12020	AT4G17570
AT3G21150	AT3G25730	AT3G46600	AT3G51960	AT3G57230	AT3G61970	AT4G01720	AT4G12050	AT4G17600
AT3G21270	AT3G25790	AT3G46640	AT3G52440	AT3G57390	AT3G62090	AT4G02235	AT4G12240	AT4G17695
AT3G21330	AT3G25890	AT3G47500	AT3G52770	AT3G57600	AT3G62100	AT4G02560	AT4G12350	AT4G17710
AT3G21880	AT3G25910	AT3G47600	AT3G53200	AT3G57670	AT3G62340	AT4G02590	AT4G12750	AT4G17750
AT3G21890	AT3G25990	AT3G47620	AT3G53340	AT3G57800	AT3G62420	AT4G02640	AT4G13040	AT4G17785
AT3G22100	AT3G26744	AT3G47640	AT3G53600	AT3G57920	AT3G62610	AT4G02670	AT4G13480	AT4G17800
AT3G22170	AT3G26790	AT3G47710	AT3G53820	AT3G58070	AT3G62670	AT4G03090	AT4G13620	AT4G17810
AT3G22760	AT3G27010	AT3G48160	AT3G53920	AT3G58120	AT3G63350	AT4G03250	AT4G13640	AT4G17880
AT3G22780	AT3G27785	AT3G48360	AT3G54220	AT3G58630	AT3G66656	AT4G04450	AT4G13980	AT4G17980
AT3G22830	AT3G27920	AT3G48590	AT3G54320	AT3G58710	AT4G00050	AT4G04580	AT4G14410	AT4G18020
AT3G23030	AT3G28470	AT3G48920	AT3G54340	AT3G58780	AT4G00120	AT4G04890	AT4G14465	AT4G18170
AT3G23050	AT3G28730	AT3G49530	AT3G54390	AT3G59060	AT4G00150	AT4G05100	AT4G14540	AT4G18390
AT3G23130	AT3G28910	AT3G49690	AT3G54620	AT3G59580	AT4G00180	AT4G05170	AT4G14550	AT4G18450
AT3G23140	AT3G29020	AT3G49760	AT3G54810	AT3G60030	AT4G00270	AT4G06746	AT4G14560	AT4G18770
AT3G23210	AT3G29035	AT3G49850	AT3G54990	AT3G60390	AT4G00480	AT4G08150	AT4G14770	AT4G18870
AT3G23220	AT3G29340	AT3G49930	AT3G55210	AT3G60460	AT4G00540	AT4G08250	AT4G15090	AT4G18880
AT3G23230	AT3G30210	AT3G49950	AT3G55370	AT3G60490	AT4G00730	AT4G09180	AT4G15250	AT4G18890
AT3G23240	AT3G30260	AT3G50060	AT3G55560	AT3G60530	AT4G00870	AT4G09450	AT4G16110	AT4G18960
								AT5G67580

AT4G19520	AT4G25410	AT4G29190	AT4G34680	AT4G37180	AT5G02840	AT5G06839	AT5G11050	AT5G16470
AT4G19630	AT4G25470	AT4G29230	AT4G34990	AT4G37260	AT5G03150	AT5G06950	AT5G11060	AT5G16560
AT4G20380	AT4G25480	AT4G29930	AT4G35040	AT4G37650	AT5G03415	AT5G06960	AT5G11190	AT5G16600
AT4G20400	AT4G25490	AT4G29940	AT4G35270	AT4G37730	AT5G03510	AT5G07100	AT5G11260	AT5G16770
AT4G20970	AT4G25530	AT4G30080	AT4G35280	AT4G37750	AT5G03680	AT5G07160	AT5G11510	AT5G16820
AT4G21030	AT4G25560	AT4G30180	AT4G35390	AT4G37780	AT5G03720	AT5G07210	AT5G11590	AT5G17260
AT4G21040	AT4G25800	AT4G30410	AT4G35550	AT4G37790	AT5G03790	AT5G07310	AT5G12840	AT5G17300
AT4G21050	AT4G26030	AT4G30935	AT4G35570	AT4G37850	AT5G04150	AT5G07500	AT5G12850	AT5G17320
AT4G21080	AT4G26150	AT4G30980	AT4G35580	AT4G37940	AT5G04340	AT5G07580	AT5G12870	AT5G17430
AT4G21330	AT4G26440	AT4G31060	AT4G35590	AT4G38000	AT5G04410	AT5G07680	AT5G13080	AT5G17490
AT4G21340	AT4G26640	AT4G31270	AT4G35610	AT4G38070	AT5G04640	AT5G07690	AT5G13180	AT5G17800
AT4G21440	AT4G26840	AT4G31420	AT4G35700	AT4G38340	AT5G04760	AT5G07700	AT5G13330	AT5G17810
AT4G21550	AT4G26930	AT4G31550	AT4G35900	AT4G38620	AT5G04840	AT5G08070	AT5G13730	AT5G18240
AT4G21750	AT4G27240	AT4G31800	AT4G36060	AT4G38900	AT5G04940	AT5G08130	AT5G13790	AT5G18270
AT4G22070	AT4G27310	AT4G31920	AT4G36160	AT4G38910	AT5G05090	AT5G08141	AT5G13910	AT5G18300
AT4G22680	AT4G27330	AT4G32010	AT4G36240	AT4G38960	AT5G05120	AT5G08190	AT5G14000	AT5G18450
AT4G22810	AT4G27410	AT4G32040	AT4G36260	AT4G39070	AT5G05410	AT5G08330	AT5G14010	AT5G18560
AT4G22950	AT4G27950	AT4G32280	AT4G36540	AT4G39100	AT5G05550	AT5G08520	AT5G14340	AT5G18830
AT4G23550	AT4G28110	AT4G32730	AT4G36570	AT4G39250	AT5G05660	AT5G08550	AT5G14490	AT5G19790
AT4G23750	AT4G28140	AT4G32800	AT4G36590	AT4G39410	AT5G05770	AT5G08790	AT5G14750	AT5G20240
AT4G23800	AT4G28500	AT4G32880	AT4G36620	AT4G39780	AT5G05790	AT5G09330	AT5G14960	AT5G20730
AT4G23810	AT4G28530	AT4G32890	AT4G36710	AT4G40060	AT5G06070	AT5G09460	AT5G15130	AT5G21120
AT4G23980	AT4G28610	AT4G32980	AT4G36730	AT5G01200	AT5G06100	AT5G09750	AT5G15150	AT5G21960
AT4G24020	AT4G28640	AT4G33450	AT4G36740	AT5G01380	AT5G06250	AT5G10030	AT5G15160	AT5G22220
AT4G24060	AT4G28790	AT4G33880	AT4G36780	AT5G01860	AT5G06500	AT5G10120	AT5G15310	AT5G22260
AT4G24240	AT4G28800	AT4G34000	AT4G36870	AT5G01900	AT5G06510	AT5G10140	AT5G15480	AT5G22290
AT4G24440	AT4G28840	AT4G34410	AT4G36900	AT5G02030	AT5G06650	AT5G10280	AT5G15800	AT5G22380
AT4G24470	AT4G29000	AT4G34530	AT4G36920	AT5G02320	AT5G06710	AT5G10510	AT5G15830	AT5G22570
AT4G24540	AT4G29080	AT4G34590	AT4G36930	AT5G02460	AT5G06770	AT5G10570	AT5G15840	AT5G22890
AT4G25400	AT4G29100	AT4G34610	AT4G36990	AT5G02470	AT5G06800	AT5G10970	AT5G15850	AT5G22990

AT5G23000	AT5G27130	AT5G39860	AT5G44180	AT5G48890	AT5G52510	AT5G57420	AT5G61430	AT5G65070
AT5G23090	AT5G27580	AT5G40120	AT5G44190	AT5G49240	AT5G52600	AT5G57520	AT5G61470	AT5G65080
AT5G23150	AT5G27810	AT5G40220	AT5G44210	AT5G49300	AT5G52660	AT5G57580	AT5G61590	AT5G65100
AT5G23260	AT5G27880	AT5G40330	AT5G44260	AT5G49330	AT5G52830	AT5G57620	AT5G61600	AT5G65130
AT5G23280	AT5G27910	AT5G40350	AT5G45050	AT5G49420	AT5G53040	AT5G57660	AT5G61620	AT5G65210
AT5G23420	AT5G27944	AT5G40360	AT5G45260	AT5G49450	AT5G53200	AT5G58010	AT5G61850	AT5G65230
AT5G23650	AT5G27960	AT5G40430	AT5G45300	AT5G49490	AT5G53210	AT5G58080	AT5G61890	AT5G65310
AT5G24110	AT5G28300	AT5G41030	AT5G45580	AT5G49520	AT5G53290	AT5G58620	AT5G62000	AT5G65320
AT5G24120	AT5G28650	AT5G41090	AT5G45710	AT5G49620	AT5G53950	AT5G58850	AT5G62020	AT5G65330
AT5G24590	AT5G28770	AT5G41200	AT5G45980	AT5G49700	AT5G53980	AT5G58890	AT5G62110	AT5G65510
AT5G24800	AT5G29000	AT5G41315	AT5G46010	AT5G50010	AT5G54070	AT5G58900	AT5G62165	AT5G65590
AT5G24930	AT5G35550	AT5G41410	AT5G46350	AT5G50080	AT5G54230	AT5G59340	AT5G62320	AT5G65640
AT5G25160	AT5G35770	AT5G41570	AT5G46590	AT5G50470	AT5G54340	AT5G59450	AT5G62380	AT5G65670
AT5G25190	AT5G37020	AT5G41920	AT5G46690	AT5G50480	AT5G54360	AT5G59570	AT5G62430	AT5G65790
AT5G25220	AT5G37260	AT5G42520	AT5G46760	AT5G50490	AT5G54470	AT5G59780	AT5G62470	AT5G66300
AT5G25390	AT5G37415	AT5G42630	AT5G46830	AT5G50570	AT5G54630	AT5G59820	AT5G62570	AT5G66320
AT5G25790	AT5G37420	AT5G42640	AT5G46880	AT5G50820	AT5G54680	AT5G60120	AT5G62610	AT5G66350
AT5G25810	AT5G37800	AT5G42910	AT5G46910	AT5G50915	AT5G55020	AT5G60200	AT5G62940	AT5G66700
AT5G25830	AT5G38140	AT5G43170	AT5G47140	AT5G51190	AT5G55390	AT5G60440	AT5G63470	AT5G66730
AT5G25890	AT5G38620	AT5G43175	AT5G47220	AT5G51230	AT5G55690	AT5G60450	AT5G63790	AT5G66770
AT5G26170	AT5G38740	AT5G43250	AT5G47230	AT5G51780	AT5G56110	AT5G60470	AT5G64060	AT5G66940
AT5G26580	AT5G38800	AT5G43270	AT5G47370	AT5G51790	AT5G56200	AT5G60690	AT5G64220	AT5G66990
AT5G26630	AT5G38860	AT5G43290	AT5G47390	AT5G51860	AT5G56270	AT5G60830	AT5G64340	AT5G67000
AT5G26650	AT5G39610	AT5G43410	AT5G47640	AT5G51870	AT5G56620	AT5G60850	AT5G64360	AT5G67010
AT5G26660	AT5G39660	AT5G43540	AT5G47660	AT5G51910	AT5G56840	AT5G60890	AT5G64530	AT5G67060
AT5G26920	AT5G39690	AT5G43650	AT5G47670	AT5G51990	AT5G56860	AT5G60910	AT5G64750	AT5G67110
AT5G26950	AT5G39700	AT5G43700	AT5G48150	AT5G52010	AT5G56930	AT5G60970	AT5G64810	AT5G67180
AT5G27050	AT5G39750	AT5G43840	AT5G48250	AT5G52020	AT5G56960	AT5G61270	AT5G64980	AT5G67190
AT5G27070	AT5G39810	AT5G44080	AT5G48560	AT5G52170	AT5G57150	AT5G61380	AT5G65050	AT5G67300
AT5G27090	AT5G39820	AT5G44160	AT5G48670	AT5G52260	AT5G57390	AT5G61420	AT5G65060	AT5G67450

Supplementary Table S3 – Results of Data mining of Gene ontology file obtained from TAIR (www.arabidopsis.org) - C) Root-related transcription factor genes

AT1G03840	AT1G79580	AT3G54220	AT5G40330
AT1G04550	AT2G02080	AT3G57670	AT5G44160
AT1G07640	AT2G14210	AT3G60630	AT5G45710
AT1G09540	AT2G18490	AT3G62100	AT5G47390
AT1G10480	AT2G22670	AT4G00150	AT5G57390
AT1G13260	AT2G24260	AT4G00730	AT5G57620
AT1G13290	AT2G25180	AT4G01060	AT5G58010
AT1G13300	AT2G27230	AT4G03090	AT5G60200
AT1G14350	AT2G28350	AT4G10350	AT5G62940
AT1G14580	AT2G30420	AT4G11880	AT5G65510
AT1G19220	AT2G37430	AT4G14550	AT5G65790
AT1G19850	AT2G37590	AT4G16110	AT5G66700
AT1G26870	AT2G40220	AT4G16780	AT1G01380
AT1G26960	AT2G45160	AT4G23750	AT3G50060
AT1G27740	AT2G45680	AT4G30080	AT5G39610
AT1G32640	AT2G46400	AT4G30980	
AT1G33280	AT2G46990	AT4G31920	
AT1G34670	AT2G47270	AT4G33880	
AT1G51190	AT2G47520	AT5G02460	
AT1G51220	AT3G03660	AT5G03150	
AT1G53910	AT3G11260	AT5G04340	
AT1G56010	AT3G15170	AT5G10510	
AT1G66470	AT3G16857	AT5G13080	
AT1G67710	AT3G17600	AT5G14750	
AT1G68320	AT3G20840	AT5G17810	
AT1G69780	AT3G20880	AT5G18560	
AT1G71692	AT3G25710	AT5G19790	
AT1G74500	AT3G30260	AT5G20730	
AT1G77850	AT3G45610	AT5G25890	

Supplementary Table S3– Results of Data mining of Gene ontology file obtained from TAIR (www.arabidopsis.org) - D) Description of members of gene tree constructed (n=102)

AT1G01380.1		AT1G01380|ETC1|ENHANCER OF TRY AND CPC 1|F6F3.18|F6F3_18
AT4G01060.1	**MYB**	AT4G01060|ETC3|CPL3|ENHANCER OF TRY AND CPC 3|CAPRICE-like MYB3|F2N1.40|F2N1_40
AT2G30420.1		AT2G30420|ETC2|ENHANCER OF TRY AND CPC 2|T9D9.23|T9D9_23|ETC2
AT2G24260.1	**bHLH**	AT2G24260|LRL1|LJRHL1-like 1|F27D4.17|F27D4_17
AT4G30980.1		AT4G30980|LRL2|LJRHL1-like 2|F6I18.110|F6I18_110
AT5G58010.1		AT5G58010|LRL3|LJRHL1-like 3|F2C19.2|F2C19_2
AT1G27740.1		AT1G27740|RSL4|root hair defective 6-like 4|T22C5.19|T22C5_19
AT4G33880.1	**bHLH**	AT4G33880|RSL2|ROOT HAIR DEFECTIVE 6-LIKE 2
AT1G66470.1		AT1G66470|RHD6|AtRHD6|ROOT HAIR DEFECTIVE6|F28G11.9|F28G11_9
AT1G32640.1	**bHLH/MYC**	AT1G32640|ATMYC2|RD22BP1|JAI1|JIN1|MYC2|ZBF1|JASMONATE INSENSITIVE 1|F6N18.4|F6N18_4|RD22BP1

AT1G07640.3		AT1G07640|OBP2|URP3|UAS-TAGGED ROOT PATTERNING3|F24B9.30|F24B9_30|ZINC FINGER PROTEIN OBP2
AT2G37590.1	**DOF**	AT2G37590|ATDOF2.4|DOF2.4|DNA binding with one finger 2.4|F13M22.9|F13M22_9
AT5G02460.1		AT5G02460|T22P11.50|T22P11_50
AT3G45610.1		AT3G45610|DOF6|DOF transcription factor 6|F9K21.190
AT5G60200.1		AT5G60200|TMO6|TARGET OF MONOPTEROS 6|F15L12.10|F15L12_10
AT5G62940.1		AT5G62940|HCA2|DOF5.6|HIGH CAMBIAL ACTIVITY2|DNA BINDING WITH ONE FINGER 5.6|MQB2.26|MQB2_26
AT1G19220.1		AT1G19220|ARF19|IAA22|ARF11|auxin response factor 19|indole-3-acetic acid inducible 22|AUXIN RESPONSE FACTOR11|T29M8.9|T29M8_9
AT5G20730.1		AT5G20730|NPH4|MSG1|IAA21|ARF7|TIR5|BIP|IAA23|IAA25|NON-PHOTOTROPHIC HYPOCOTYL|MASSUGU 1|indole-3-acetic acid inducible 21|AUXIN RESPONSE FACTOR 7|TRANSPORT INHIBITOR RESPONSE 5|BIPOSTO|indole-3-acetic acid inducible 23|indole-3-acetic acid inducible 25|T1M15.130|T1M15_130|AUXIN-RESPONSIVE TRANSCRIPTIONAL ACTIVATOR 7|AUXIN-REGULATED TRANSCRIPTIONAL ACTIVATOR 7

AT1G19850.1	**ARFs**	AT1G19850|MP|ARF5|IAA24|MONOPTEROS|AUXIN RESPONSE FACTOR 5|indole-3-acetic acid inducible 24|F6F9.10|F6F9_10|TRANSCRIPTION FACTOR IAA24
AT1G77850.1		AT1G77850|ARF17|auxin response factor 17|F28K19.6|F28K19_6
AT2G28350.1		AT2G28350|ARF10|auxin response factor 10|T1B3.13|T1B3_13
AT4G30080.1		AT4G30080|ARF16|auxin response factor 16|F6G3.110|F6G3_110

AT1G03840.1		AT1G03840|MGP|IDD3|Magpie|INDETERMINATE DOMAIN 3|F21M11.25|F21M11_25
AT5G44160.1		AT5G44160|NUC|IDD8|AtIDD8|nutcracker|INDETERMINATE DOMAIN 8|MLN1.8|MLN1_8
AT1G14580.1	**IDD-domain**	AT1G14580|T5E21.8|T5E21_8
AT2G02080.1		AT2G02080|AtIDD4|IDD4|indeterminate(ID)-domain 4|F5O4.15|F5O4_15
AT5G03150.1		AT5G03150|JKD|JACKDAW|F15A17.180|F15A17_180
AT1G13290.1	**C2H2**	AT1G13290|DOT5|WIP6|DEFECTIVELY ORGANIZED TRIBUTARIES 5|WIP domain protein 6|T6J4.5|T6J4_5
AT3G57670.1	**C2H2/C2HC**	AT3G57670|NTT|WIP2|NO TRANSMITTING TRACT|WIP domain protein 2|F15B8.140
AT1G51220.1	**WIP domain**	AT1G51220.1|WIP5|WIP DOMAIN PROTEIN 5, WIP5
AT3G20880.1		AT3G20880|WIP4|WIP domain protein 4|MOE17.19
AT2G18490.1		GAZ
AT2G45680.1	**TCP domain**	AT2G45680|TCP9|TCP domain protein 9|F17K2.21

AT1G04550.2		AT1G04550|IAA12|BDL|indole-3-acetic acid inducible 12|BODENLOS|T1G11.20|T1G11_20
AT2G22670.4		AT2G22670|IAA8|indoleacetic acid-induced protein 8|T9I22.11|T9I22_11
AT4G14550.1	**Aux/IAA family**	AT4G14550|IAA14|SLR|indole-3-acetic acid inducible 14|SOLITARY ROOT|DL3315C|FCAALL.254
AT5G25890.1		AT5G25890|IAA28|IAR2|indole-3-acetic acid inducible 28|IAA-ALANINE RESISTANT 2|T1N24.24|T1N24_24
AT2G46990.1		AT2G46990|IAA20|indole-3-acetic acid inducible 20|F14M4.18
AT3G62100.1		AT3G62100|IAA30|indole-3-acetic acid inducible 30|T17J13.60

AT3G17600.1		AT3G17600|IAA31|indole-3-acetic acid inducible 31|MKP6.16
AT2G46400.1	**WRKY**	AT2G46400|WRKY46|ATWRKY46|WRKY DNA-binding protein 46|F11C10.9
AT5G13080.1		AT5G13080|WRKY75|ATWRKY75|WRKY DNA-binding protein 75|ARABIDOPSIS THALIANA WRKY DNA-BINDING PROTEIN 75|T19L5.40|T19L5_40

AT1G26870.1		AT1G26870|FEZ|ANAC009|FEZ|Arabidopsis NAC domain containing protein 9|T2P11.6|T2P11_6|FEZ
AT1G33280.1		AT1G33280|ANAC015|BRN1|NAC015|NAC domain containing protein 15|BEARSKIN 1|T16O9.16|T16O9_16
AT4G10350.1		AT4G10350|ANAC070|BRN2|NAC070|NAC domain containing protein 70|BEARSKIN 2|F24G24.150|F24G24_150
AT1G79580.1	**NAC domain**	AT1G79580|SMB|ANAC033|URP7|SOMBRERO|Arabidopsis NAC domain containing protein 33|UAS-TAGGED ROOT PATTERNING7|F20B17.1|F20B17_1|SMB
AT1G56010.2		AT1G56010|NAC1|anac021|ANAC022|NAC domain containing protein 1|Arabidopsis NAC domain containing protein 21|Arabidopsis NAC domain containing protein 22|F14J16.32
AT3G15170.1		AT3G15170|CUC1|ANAC054|ATNAC1|CUP-SHAPED COTYLEDON1|Arabidopsis NAC domain containing protein 54|F4B12.8
AT5G39610.1		AT5G39610|ATNAC2|ORE1|ANAC092|ATNAC6|NAC2|NAC6|NAC domain containing protein 2|ORESARA 1|Arabidopsis NAC domain containing protein 92|NAC domain containing protein 6|MIJ24.11|MIJ24_11
AT2G47270.1		AT2G47270|UPB1|UPBEAT1|T8I13.11

AT1G74500.1		AT1G74500|ATBS1|TMO7|BS1|PRE3|bHLH135|activation-tagged BRI1(brassinosteroid-insensitive 1)-suppressor 1|TARGET OF MONOPTEROS 7|PACLOBUTRAZOL RESISTANCE 3|basic helix-loop-helix protein 135|F1M20.18|F1M20_18
AT3G11260.1	**WUS**	AT3G11260|WOX5|WOX5B|WUSCHEL related homeobox 5|WUSCHEL related homeobox 5B|F11B9.18
AT3G54220.1		AT3G54220|SCR|SGR1|SCARECROW|SHOOT GRAVITROPISM 1|F24B22.180

AT3G25710.1	**bHLH**	AT3G25710|BHLH32|ATAIG1|TMO5|basic helix-loop-helix 32|TARGET OF MONOPTEROS 5|K13N2.1|BHLH32
AT4G16780.1		AT4G16780|ATHB-2|HAT4|ATHB2|HB-2|homeobox protein 2|ARABIDOPSIS THALIANA HOMEOBOX PROTEIN 2|DL4415W|FCAALL.101
AT1G09540.1		AT1G09540|MYB61|ATMYB61|myb domain protein 61|ARABIDOPSIS THALIANA MYB DOMAIN PROTEIN 61|F14J9.20|F14J9_20
AT1G34670.1		AT1G34670|AtMYB93|MYB93|myb domain protein 93|F21H2.9|F21H2_9
AT5G14750.1		AT5G14750|WER|ATMYB66|WER1|MYB66|WEREWOLF|myb domain protein 66|WEREWOLF 1|T9L3.50|T9L3_50
AT5G40330.1	**MYB**	AT5G40330|MYB23|ATMYB23|ATMYBRTF|myb domain protein 23|MPO12.40|MPO12_40
AT5G57620.1		AT5G57620|MYB36|AtMYB36|myb domain protein 36|MUA2.20|MUA2_20
AT5G65790.1		AT5G65790|ATMYB68|MYB68|MYB DOMAIN PROTEIN 68|MPA24.14|MPA24_14
AT1G68320.1		AT1G68320|MYB62|AtMYB62|BW62B|BW62C|myb domain protein 62|T22E19.5|T22E19_5
AT3G50060.1		AT3G50060|MYB77|myb domain protein 77|F3A4.140
AT1G14350.1		AT1G14350|FLP|AtMYB124|MYB124|FOUR LIPS|myb domain protein 124|F14L17.12|F14L17_12
AT1G13260.1		AT1G13260|RAV1|EDF4|AtRAV1|related to ABI3/VP1 1|ETHYLENE RESPONSE DNA BINDING FACTOR 4|T6J4.2|T6J4_2
AT1G51190.1	**AP2-domain**	AT1G51190|PLT2|PLETHORA 2|F11M15.6|F11M15_6
AT3G20840.1		AT3G20840|PLT1|PLETHORA 1|MOE17.15
AT5G57390.1		AT5G57390|AIL5|CHO1|EMK|PLT5|AINTEGUMENTA-like 5|CHOTTO 1|EMBRYOMAKER|PLETHORA 5|MSF19.5|MSF19_5
AT5G10510.3		AT5G10510|AIL6|PLT3|AINTEGUMENTA-like 6|PLETHORA 3|F12B17.140|F12B17_140
AT5G65510.1		AT5G65510|AIL7|PLT7|AINTEGUMENTA-like 7|PLETHORA 7|K21L13.1|K21L13_1
AT1G53910.1		AT1G53910|RAP2.12|related to AP2 12|T18A20.14|T18A20_14
AT2G47520.1		AT2G47520|HRE2|ERF71|AtERF71|HYPOXIA RESPONSIVE ERF (ETHYLENE RESPONSE FACTOR) 2|ethylene response factor 71|Arabidopsis thaliana ethylene response factor 71|T30B22.18

AT4G23750.1	**ERF/AP2**	AT4G23750|CRF2|TMO3|cytokinin response factor 2|TARGET OF MONOPTEROS 3|F9D16.220|F9D16_220
AT5G19790.1		AT5G19790|RAP2.11|related to AP2 11|T29J13.210|T29J13_210
AT5G18560.1		AT5G18560|PUCHI|T28N17.40|T28N17_40
AT2G40220.1		AT2G40220|ABI4|SUN6|SIS5|ISI3|GIN6|SAN5|ATABI4|ABA INSENSITIVE 4|SUCROSE UNCOUPLED 6|SUGAR-INSENSITIVE 5|IMPAIRED SUCROSE INDUCTION 3|GLUCOSE INSENSITIVE 6|SALOBRENO 5|T7M7.16|ABSCISIC ACID-INSENSITIVE PROTEIN 4
AT1G13300.1	**GARP**	AT1G13300|HRS1|AtNIGT1|HYPERSENSITIVITY TO LOW PI-ELICITED PRIMARY ROOT SHORTENING 1|T6J4.6|T6J4_6
AT1G67710.1		AT1G67710|ARR11|response regulator 11|F12A21.15|F12A21_15
AT3G16857.2		AT3G16857|ARR1|RR1|response regulator 1|MUH15.5
AT4G16110.1	**ARR family**	AT4G16110|ARR2|RR2|response regulator 2|DL4095W|FCAALL.287
AT2G25180.1		AT2G25180|ARR12|RR12|AtARR12|response regulator 12|F13D4.140|F13D4_140
AT4G31920.1		AT4G31920|ARR10|RR10|response regulator 10|F11C18.3
AT5G47390.1		AT5G47390|MYBH|KUA1|MYB hypocotyl elongation-related|KUODA1 (Chinese for enlarge or expand)|MQL5.25|MQL5_25
AT3G03660.2		WUSCHEL-related homeobox gene family member
AT5G17810.1		WUSCHEL-related homeobox gene family member
AT4G00730.1	**homeodomain**	AT4G00730|ANL2|AHDP|ANTHOCYANINLESS 2|ARABIDOPSIS THALIANA HOMEODOMAIN PROTEIN|F6N23.10|F6N23_10|HOMEODOMAIN PROTEIN AHDP
AT1G10480.1		AT1G10480|ZFP5|zinc finger protein 5|T10O24.9|T10O24_9
AT1G26960.1	**HD-Zip I**	AT1G26960|AtHB23|HB23|homeobox protein 23|T2P11.15|T2P11_15
AT1G69780.1		AT1G69780|ATHB13|T6C23.2|T6C23_2|HOMEODOMAIN LEUCINE-ZIPPER PROTEIN ATHB13
AT5G66700.1		AT5G66700|HB53|HB-8|ATHB53|homeobox 53|HOMEOBOX-8|ARABIDOPSIS THALIANA HOMEOBOX 53|MSN2.9|MSN2_9
AT1G71692.1		AT1G71692|AGL12|XAL1|AGAMOUS-like 12|XAANTAL1|F14O23.5|F14O23_5

AT2G14210.1	**MADS-box**	AT2G14210|ANR1|AGL44|ARABIDOPSIS NITRATE REGULATED 1|AGAMOUS-like 44|F15N24.5|F15N24_5
AT3G30260.1		AT3G30260|AGL79|AGAMOUS-like 79|T6J22.1
AT4G11880.1		AT4G11880|AGL14|XAL2|AGAMOUS-like 14|XAANTAL2|T26M18.90|T26M18_90
AT2G37430.1		AT2G37430|ZAT11|zinc finger of Arabidopsis thaliana 11|F3G5.22|F3G5_22
AT5G04340.1		AT5G04340|C2H2|CZF2|ZAT6|AtZAT6|COLD INDUCED ZINC FINGER PROTEIN 2|zinc finger of Arabidopsis thaliana 6|T19N18.70|T19N18_70|C2H2 ZINC FINGER TRANSCRIPTION FACTOR
AT2G27230.1		AT2G27230|LHW|LONESOME HIGHWAY|F12K2.19|F12K2_19
AT4G03090.1		AT4G03090|NDX|AtNDX|Nodulin Homeobox|T4I9.3|T4I9_3
AT2G45160.1		AT2G45160|HAM1|ATHAM1|LOM1|SCL27|HAIRY MERISTEM 1|ARABIDOPSIS THALIANA HAIRY MERISTEM 1|LOST MERISTEMS 1|SCARECROW-LIKE 27|T14P1.3
AT3G60630.1	**LOM**	AT3G60630|HAM2|ATHAM2|LOM2|HAIRY MERISTEM 2|ARABIDOPSIS THALIANA HAIRY MERISTEM 2|LOST MERISTEMS 2|T4C21.40
AT4G00150.1		AT4G00150|SCL6-IV|HAM3|ATHAM3|LOM3|HAIRY MERISTEM 3|ARABIDOPSIS THALIANA HAIRY MERISTEM 3|LOST MERISTEMS 3|F6N15.20|F6N15_20
AT5G45710.1	**HSF**	AT5G45710|AT-HSFA4C|RHA1|HSFA4C|HEAT SHOCK TRANSCRIPTION FACTOR A4C|ROOT HANDEDNESS 1|MRA19.11|MRA19_11
