# Supplementary material for: The rolB‐transgenic Nicotiana tabacum plants exhibit upregulated ARF7 and ARF19 gene expression
Source: Plant Direct. 2022 Jun 18;6(6):e414. doi: 10.1002/pld3.414 (PMC9219009; doi:10.1002/pld3.414)
Supplement: Supplementary file 11 — Table S4 Co‐expressed genes of A) ARF7‐ARF19; B) ARF5 and C) ARF6‐ ARF8 as obtained from CORNET online database. [file PLD3-6-e414-s009.pdf]

Supplementary Table S4 – A) Co-expressed genes of *ARF7-ARF19* as obtained from CORNET online database

AT1G02020	AT1G23440	AT1G71980	AT2G24040	AT2G45910	AT3G23980	AT3G55320	AT4G21450	AT4G35040	AT5G12330	AT5G45430
AT1G02120	AT1G24560	AT1G72840	AT2G25070	AT2G45960	AT3G24180	AT3G55450	AT4G21580	AT4G35230	AT5G15730	AT5G47690
AT1G02890	AT1G27320	AT1G73430	AT2G25590	AT2G46410	AT3G24550	AT3G56310	AT4G22270	AT4G35380	AT5G16280	AT5G48560
AT1G02960	AT1G29340	AT1G73590	AT2G26510	AT2G47410	AT3G25610	AT3G59700	AT4G24060	AT4G35510	AT5G16570	AT5G49660
AT1G03090	AT1G29950	AT1G73960	AT2G28810	AT2G47860	AT3G26100	AT3G60030	AT4G24400	AT4G35580	AT5G16910	AT5G50230
AT1G03740	AT1G30400	AT1G74360	AT2G30800	AT2G47980	AT3G26890	AT3G60240	AT4G24990	AT4G36060	AT5G19420	AT5G52510
AT1G05340	AT1G30760	AT1G74660	AT2G30840	AT3G01860	AT3G27570	AT3G61850	AT4G25434	AT4G37295	AT5G19450	AT5G53320
AT1G06840	AT1G32130	AT1G74840	AT2G31960	AT3G02740	AT3G46000	AT3G62100	AT4G25970	AT4G37590	AT5G19530	AT5G53440
AT1G07310	AT1G36160	AT1G76920	AT2G32250	AT3G03570	AT3G47550	AT3G62150	AT4G26750	AT4G39140	AT5G20730	AT5G53570
AT1G08430	AT1G49310	AT1G77130	AT2G32730	AT3G04030	AT3G48760	AT3G62650	AT4G27450	AT4G39680	AT5G22760	AT5G54130
AT1G08720	AT1G49320	AT1G78120	AT2G33310	AT3G04570	AT3G48860	AT3G62770	AT4G28300	AT4G39720	AT5G23110	AT5G55560
AT1G09570	AT1G50140	AT1G78380	AT2G34140	AT3G04880	AT3G48890	AT3G63070	AT4G28470	AT5G01400	AT5G23450	AT5G55600
AT1G10940	AT1G52150	AT1G79280	AT2G34830	AT3G06670	AT3G49350	AT3G63440	AT4G28640	AT5G03320	AT5G24740	AT5G55860
AT1G12470	AT1G54490	AT1G80070	AT2G35270	AT3G07690	AT3G49490	AT4G00940	AT4G29040	AT5G03380	AT5G27840	AT5G56170
AT1G15780	AT1G55610	AT2G01350	AT2G35630	AT3G07780	AT3G49760	AT4G01210	AT4G29100	AT5G03520	AT5G35200	AT5G58040
AT1G16310	AT1G58250	AT2G01650	AT2G36850	AT3G09850	AT3G49810	AT4G01370	AT4G29350	AT5G03540	AT5G38210	AT5G58270
AT1G18160	AT1G59890	AT2G02560	AT2G37280	AT3G10740	AT3G50370	AT4G01550	AT4G29380	AT5G04240	AT5G38280	AT5G58380
AT1G18470	AT1G60200	AT2G02800	AT2G38120	AT3G11230	AT3G50380	AT4G01610	AT4G29820	AT5G04750	AT5G38560	AT5G58410
AT1G18880	AT1G61660	AT2G03150	AT2G38800	AT3G11910	AT3G51120	AT4G04920	AT4G30960	AT5G05140	AT5G38710	AT5G59880
AT1G19220	AT1G62770	AT2G06210	AT2G39260	AT3G12360	AT3G51370	AT4G08180	AT4G31170	AT5G05160	AT5G39950	AT5G60200
AT1G19870	AT1G65440	AT2G13370	AT2G39370	AT3G12590	AT3G51580	AT4G13030	AT4G31450	AT5G05960	AT5G40250	AT5G60450
AT1G20110	AT1G66480	AT2G14120	AT2G39870	AT3G12680	AT3G51830	AT4G13510	AT4G31880	AT5G06120	AT5G42220	AT5G60690
AT1G20160	AT1G66880	AT2G20550	AT2G42160	AT3G14430	AT3G52240	AT4G14580	AT4G31910	AT5G07920	AT5G42520	AT5G61140
AT1G20700	AT1G67140	AT2G21230	AT2G42490	AT3G14990	AT3G52710	AT4G15090	AT4G32010	AT5G08560	AT5G43180	AT5G61910
AT1G21200	AT1G68810	AT2G21620	AT2G42610	AT3G15880	AT3G53980	AT4G16650	AT4G32600	AT5G10450	AT5G43560	AT5G63710
AT1G21610	AT1G69580	AT2G22300	AT2G43210	AT3G19200	AT3G53990	AT4G17080	AT4G32880	AT5G10520	AT5G43900	AT5G63930
AT1G21980	AT1G70520	AT2G23030	AT2G43680	AT3G19370	AT3G54230	AT4G18120	AT4G33240	AT5G11670	AT5G44180	AT5G64220
AT1G22280	AT1G70530	AT2G23460	AT2G44100	AT3G19740	AT3G54280	AT4G18880	AT4G33430	AT5G12010	AT5G44350	AT5G64340
AT1G22930	AT1G70920	AT2G23980	AT2G45420	AT3G21630	AT3G55070	AT4G19370	AT4G33910	AT5G12210	AT5G44790	AT5G64341

AT5G65430 AT5G65210 AT5G66120 AT5G66250 ATMG01000 AT5G64930

Supplementary Table S4 – B) Co-expressed genes of *ARF5* as obtained from CORNET online database

AT1G01110	AT1G05440	AT1G09470	AT1G13710	AT1G18520	AT1G23040	AT1G27370	AT1G42470	AT1G52500	AT1G58210	AT1G64300
AT1G01370	AT1G05470	AT1G09600	AT1G13790	AT1G18550	AT1G23190	AT1G28110	AT1G44110	AT1G52570	AT1G58430	AT1G64450
AT1G01510	AT1G05490	AT1G09610	AT1G13970	AT1G18950	AT1G23240	AT1G28270	AT1G44760	AT1G52680	AT1G59540	AT1G64628
AT1G01900	AT1G05710	AT1G09760	AT1G14180	AT1G19100	AT1G23250	AT1G28375	AT1G44780	AT1G53050	AT1G59560	AT1G64630
AT1G02110	AT1G05910	AT1G09910	AT1G14440	AT1G19780	AT1G23520	AT1G28430	AT1G44900	AT1G53070	AT1G59640	AT1G64660
AT1G02180	AT1G05950	AT1G10120	AT1G14900	AT1G19830	AT1G23570	AT1G28470	AT1G45160	AT1G53140	AT1G59660	AT1G64800
AT1G02190	AT1G06250	AT1G10180	AT1G15480	AT1G19850	AT1G23580	AT1G29140	AT1G46264	AT1G53165	AT1G59890	AT1G64960
AT1G02690	AT1G06260	AT1G10230	AT1G15570	AT1G19880	AT1G23590	AT1G30300	AT1G47340	AT1G53860	AT1G60220	AT1G65700
AT1G02730	AT1G06420	AT1G10640	AT1G15660	AT1G19890	AT1G23610	AT1G30330	AT1G47570	AT1G54180	AT1G60860	AT1G65900
AT1G02790	AT1G06450	AT1G10770	AT1G15940	AT1G19940	AT1G23670	AT1G30350	AT1G47870	AT1G54280	AT1G61010	AT1G66250
AT1G02800	AT1G06520	AT1G10780	AT1G16070	AT1G19980	AT1G23790	AT1G30490	AT1G48050	AT1G54340	AT1G61110	AT1G66530
AT1G02840	AT1G06590	AT1G10850	AT1G16220	AT1G20080	AT1G23800	AT1G30600	AT1G48270	AT1G54440	AT1G61450	AT1G66850
AT1G02970	AT1G06760	AT1G10980	AT1G16290	AT1G20120	AT1G24620	AT1G31150	AT1G48280	AT1G54450	AT1G61700	AT1G67040
AT1G03050	AT1G06960	AT1G11130	AT1G16360	AT1G20410	AT1G25470	AT1G31310	AT1G48410	AT1G54460	AT1G61860	AT1G67320
AT1G03270	AT1G06990	AT1G11190	AT1G16520	AT1G20910	AT1G25472	AT1G31360	AT1G48580	AT1G54560	AT1G62450	AT1G67630
AT1G03330	AT1G07270	AT1G11420	AT1G16680	AT1G20930	AT1G25510	AT1G31720	AT1G48610	AT1G54610	AT1G62500	AT1G67690
AT1G03620	AT1G07410	AT1G11570	AT1G16705	AT1G21090	AT1G25540	AT1G31760	AT1G49740	AT1G54650	AT1G62870	AT1G67720
AT1G03780	AT1G07540	AT1G11600	AT1G16780	AT1G21560	AT1G26190	AT1G32190	AT1G49870	AT1G54840	AT1G62950	AT1G68120
AT1G03830	AT1G07790	AT1G11730	AT1G16900	AT1G21690	AT1G26260	AT1G32250	AT1G49910	AT1G54850	AT1G62990	AT1G68430
AT1G04020	AT1G07795	AT1G11940	AT1G17110	AT1G21700	AT1G26300	AT1G32730	AT1G50110	AT1G54920	AT1G63020	AT1G68640
AT1G04050	AT1G07850	AT1G11950	AT1G17140	AT1G21740	AT1G26330	AT1G32930	AT1G50240	AT1G54960	AT1G63060	AT1G68750
AT1G04090	AT1G07970	AT1G12070	AT1G17350	AT1G21810	AT1G26370	AT1G33360	AT1G50310	AT1G55130	AT1G63100	AT1G69400
AT1G04590	AT1G08040	AT1G12200	AT1G17920	AT1G21880	AT1G26480	AT1G33410	AT1G50490	AT1G55200	AT1G63160	AT1G69420
AT1G04600	AT1G08130	AT1G12370	AT1G18040	AT1G22130	AT1G26540	AT1G33420	AT1G50660	AT1G55350	AT1G63180	AT1G69700
AT1G04670	AT1G08260	AT1G12430	AT1G18180	AT1G22150	AT1G26590	AT1G34065	AT1G50710	AT1G55560	AT1G63470	AT1G69770
AT1G04760	AT1G08560	AT1G12600	AT1G18190	AT1G22460	AT1G26610	AT1G34340	AT1G51410	AT1G55570	AT1G63480	AT1G69910
AT1G04880	AT1G08620	AT1G12830	AT1G18250	AT1G22730	AT1G26760	AT1G34355	AT1G51460	AT1G55690	AT1G63640	AT1G70210
AT1G05040	AT1G08730	AT1G13030	AT1G18280	AT1G22760	AT1G26820	AT1G34770	AT1G51670	AT1G56210	AT1G63650	AT1G70470
AT1G05120	AT1G08780	AT1G13120	AT1G18370	AT1G22930	AT1G27120	AT1G35490	AT1G52150	AT1G57700	AT1G63710	AT1G70510
AT1G05360	AT1G09450	AT1G13220	AT1G18450	AT1G23000	AT1G27360	AT1G35780	AT1G52310	AT1G57820	AT1G64060	AT1G70710

AT1G71310	AT1G76250	AT1G79820	AT2G06200	AT2G17950	AT2G23800	AT2G29040	AT2G34650	AT2G39090	AT2G45040	AT3G03430
AT1G71680	AT1G76310	AT1G79950	AT2G06510	AT2G18100	AT2G24230	AT2G29050	AT2G34670	AT2G39300	AT2G45460	AT3G04620
AT1G71760	AT1G76420	AT1G80070	AT2G07170	AT2G18230	AT2G24350	AT2G29210	AT2G34710	AT2G39510	AT2G45490	AT3G04630
AT1G71770	AT1G76510	AT1G80280	AT2G07560	AT2G18330	AT2G24440	AT2G29550	AT2G34790	AT2G39820	AT2G45700	AT3G04680
AT1G71790	AT1G76540	AT1G80350	AT2G07690	AT2G18470	AT2G24490	AT2G29570	AT2G34920	AT2G39830	AT2G45870	AT3G04810
AT1G71830	AT1G76660	AT1G80370	AT2G07708	AT2G18520	AT2G24630	AT2G29790	AT2G35160	AT2G39870	AT2G46000	AT3G04960
AT1G72250	AT1G76710	AT1G80660	AT2G13350	AT2G19110	AT2G25060	AT2G29890	AT2G35310	AT2G40030	AT2G46180	AT3G04980
AT1G72290	AT1G76740	AT2G01120	AT2G13540	AT2G19170	AT2G25270	AT2G29940	AT2G35340	AT2G40550	AT2G47050	AT3G05110
AT1G72550	AT1G76820	AT2G01210	AT2G13570	AT2G19330	AT2G25420	AT2G30080	AT2G35530	AT2G40640	AT2G47230	AT3G05190
AT1G72670	AT1G76850	AT2G01330	AT2G13680	AT2G19620	AT2G25880	AT2G30290	AT2G35605	AT2G40790	AT2G47620	AT3G05330
AT1G72730	AT1G77260	AT2G01630	AT2G13690	AT2G19770	AT2G25970	AT2G30630	AT2G35630	AT2G40930	AT2G47720	AT3G05470
AT1G72960	AT1G77270	AT2G01750	AT2G14095	AT2G20000	AT2G26180	AT2G31270	AT2G35690	AT2G41210	AT2G47900	AT3G05600
AT1G73490	AT1G77390	AT2G02170	AT2G14490	AT2G20100	AT2G26700	AT2G31320	AT2G36010	AT2G41350	AT3G01020	AT3G05670
AT1G73590	AT1G77470	AT2G02540	AT2G15535	AT2G20240	AT2G26760	AT2G31650	AT2G36020	AT2G41520	AT3G01240	AT3G05720
AT1G73700	AT1G77600	AT2G02970	AT2G15860	AT2G20300	AT2G27040	AT2G31725	AT2G36190	AT2G41550	AT3G01250	AT3G05740
AT1G73850	AT1G77720	AT2G03060	AT2G16090	AT2G20440	AT2G27170	AT2G31780	AT2G36200	AT2G41650	AT3G01270	AT3G05750
AT1G74150	AT1G77980	AT2G03070	AT2G16270	AT2G20480	AT2G27180	AT2G31970	AT2G36350	AT2G41890	AT3G01330	AT3G05830
AT1G74390	AT1G78160	AT2G03200	AT2G16360	AT2G20710	AT2G27228	AT2G32410	AT2G37080	AT2G42010	AT3G01410	AT3G05960
AT1G74420	AT1G78430	AT2G03210	AT2G16390	AT2G21050	AT2G27230	AT2G32590	AT2G37210	AT2G42120	AT3G01700	AT3G06030
AT1G75090	AT1G78650	AT2G03240	AT2G16440	AT2G21770	AT2G27740	AT2G32670	AT2G37290	AT2G42200	AT3G02000	AT3G06130
AT1G75110	AT1G78770	AT2G03280	AT2G16450	AT2G21800	AT2G27970	AT2G32765	AT2G37300	AT2G42800	AT3G02210	AT3G06220
AT1G75150	AT1G78940	AT2G03810	AT2G16640	AT2G22180	AT2G27990	AT2G32890	AT2G37420	AT2G42990	AT3G02310	AT3G06740
AT1G75290	AT1G78960	AT2G03830	AT2G16780	AT2G22390	AT2G28070	AT2G33410	AT2G37510	AT2G43040	AT3G02640	AT3G07060
AT1G75310	AT1G79250	AT2G03850	AT2G17370	AT2G22420	AT2G28130	AT2G33560	AT2G37610	AT2G43230	AT3G02680	AT3G07160
AT1G75520	AT1G79350	AT2G04020	AT2G17410	AT2G22560	AT2G28320	AT2G33610	AT2G38080	AT2G43800	AT3G02820	AT3G07170
AT1G75620	AT1G79360	AT2G04675	AT2G17530	AT2G22610	AT2G28355	AT2G33620	AT2G38110	AT2G43990	AT3G02890	AT3G07200
AT1G75640	AT1G79420	AT2G05210	AT2G17560	AT2G23360	AT2G28380	AT2G33640	AT2G38160	AT2G44190	AT3G02920	AT3G07320
AT1G75920	AT1G79460	AT2G05760	AT2G17620	AT2G23380	AT2G28590	AT2G33690	AT2G38370	AT2G44260	AT3G02950	AT3G07410
AT1G75930	AT1G79640	AT2G05790	AT2G17800	AT2G23530	AT2G28620	AT2G33860	AT2G38580	AT2G44690	AT3G03080	AT3G07540
AT1G75940	AT1G79690	AT2G05910	AT2G17940	AT2G23700	AT2G28790	AT2G34190	AT2G38810	AT2G44830	AT3G03130	AT3G07820

AT3G07880	AT3G12680	AT3G17630	AT3G22780	AT3G26932	AT3G45810	AT3G51070	AT3G54350	AT3G58750	AT3G63300	AT4G10090
AT3G08560	AT3G12870	AT3G17680	AT3G22790	AT3G27330	AT3G45870	AT3G51250	AT3G54390	AT3G58770	AT4G00020	AT4G10420
AT3G08680	AT3G13000	AT3G17820	AT3G22990	AT3G27360	AT3G46020	AT3G51280	AT3G54560	AT3G58780	AT4G00350	AT4G10440
AT3G08990	AT3G13060	AT3G18210	AT3G23060	AT3G27440	AT3G46200	AT3G51290	AT3G54630	AT3G59420	AT4G00820	AT4G10850
AT3G09070	AT3G13065	AT3G18360	AT3G23270	AT3G27470	AT3G46440	AT3G51300	AT3G54670	AT3G59530	AT4G00990	AT4G10930
AT3G09560	AT3G13175	AT3G18520	AT3G23670	AT3G28500	AT3G46520	AT3G51310	AT3G54820	AT3G59550	AT4G01470	AT4G11030
AT3G09670	AT3G13190	AT3G18524	AT3G23890	AT3G28730	AT3G46750	AT3G51490	AT3G55000	AT3G60100	AT4G01730	AT4G11080
AT3G09730	AT3G13390	AT3G18660	AT3G23900	AT3G28750	AT3G46770	AT3G51630	AT3G55050	AT3G60740	AT4G02060	AT4G11130
AT3G09900	AT3G13400	AT3G18880	AT3G24090	AT3G28780	AT3G47440	AT3G51632	AT3G55110	AT3G60830	AT4G02070	AT4G11450
AT3G09930	AT3G13510	AT3G18910	AT3G24280	AT3G28790	AT3G47460	AT3G51720	AT3G55590	AT3G60840	AT4G02140	AT4G11560
AT3G10180	AT3G13640	AT3G19090	AT3G24340	AT3G28830	AT3G48210	AT3G51740	AT3G55620	AT3G60900	AT4G02150	AT4G11760
AT3G10310	AT3G13960	AT3G19120	AT3G24440	AT3G28840	AT3G48425	AT3G52090	AT3G55660	AT3G61250	AT4G02250	AT4G12700
AT3G10320	AT3G14000	AT3G19300	AT3G24450	AT3G28980	AT3G48480	AT3G52110	AT3G55890	AT3G61310	AT4G02290	AT4G12960
AT3G10540	AT3G14190	AT3G19590	AT3G24490	AT3G29060	AT3G48710	AT3G52540	AT3G56100	AT3G61490	AT4G02460	AT4G13230
AT3G10590	AT3G14800	AT3G19790	AT3G24495	AT3G29070	AT3G48900	AT3G52620	AT3G56120	AT3G61610	AT4G02485	AT4G13960
AT3G10730	AT3G14890	AT3G20010	AT3G24630	AT3G29280	AT3G49250	AT3G52780	AT3G56370	AT3G61670	AT4G02560	AT4G14150
AT3G10880	AT3G14980	AT3G20020	AT3G24640	AT3G29390	AT3G49490	AT3G52810	AT3G56600	AT3G61760	AT4G02710	AT4G14200
AT3G10890	AT3G15150	AT3G20150	AT3G24660	AT3G42170	AT3G49600	AT3G52890	AT3G56640	AT3G61830	AT4G02800	AT4G14330
AT3G11000	AT3G15400	AT3G20180	AT3G24810	AT3G42640	AT3G49890	AT3G53080	AT3G56870	AT3G61960	AT4G03100	AT4G14368
AT3G11040	AT3G15510	AT3G20260	AT3G24890	AT3G42660	AT3G49900	AT3G53140	AT3G56960	AT3G62050	AT4G03270	AT4G14490
AT3G11160	AT3G15550	AT3G20475	AT3G25100	AT3G43120	AT3G50070	AT3G53190	AT3G57060	AT3G62170	AT4G04080	AT4G14695
AT3G11500	AT3G16040	AT3G20560	AT3G25150	AT3G43210	AT3G50110	AT3G53350	AT3G57300	AT3G62180	AT4G04460	AT4G14720
AT3G11520	AT3G16150	AT3G20670	AT3G25160	AT3G43610	AT3G50170	AT3G53380	AT3G57390	AT3G62230	AT4G04930	AT4G14770
AT3G11730	AT3G16170	AT3G20740	AT3G25165	AT3G43840	AT3G50220	AT3G53520	AT3G57690	AT3G62300	AT4G05190	AT4G14780
AT3G12060	AT3G16490	AT3G20865	AT3G25400	AT3G43920	AT3G50410	AT3G53650	AT3G57830	AT3G62620	AT4G05520	AT4G14920
AT3G12160	AT3G16730	AT3G21000	AT3G25980	AT3G44050	AT3G50530	AT3G53730	AT3G57860	AT3G62640	AT4G08310	AT4G14930
AT3G12170	AT3G16980	AT3G21480	AT3G26125	AT3G44960	AT3G50780	AT3G53760	AT3G57920	AT3G62840	AT4G08670	AT4G14950
AT3G12380	AT3G17160	AT3G21970	AT3G26370	AT3G45010	AT3G50870	AT3G54080	AT3G58160	AT3G63130	AT4G09140	AT4G14970
AT3G12550	AT3G17220	AT3G22220	AT3G26750	AT3G45090	AT3G50890	AT3G54250	AT3G58540	AT3G63240	AT4G09510	AT4G15140
AT3G12660	AT3G17360	AT3G22520	AT3G26850	AT3G45280	AT3G51030	AT3G54340	AT3G58650	AT3G63250	AT4G09960	AT4G15790

AT4G15830	AT4G22680	AT4G27370	AT4G31840	AT4G36350	AT5G03510	AT5G07720	AT5G13820	AT5G18410	AT5G25150	AT5G39650
AT4G15890	AT4G22730	AT4G27480	AT4G31880	AT4G37210	AT5G03590	AT5G07810	AT5G13840	AT5G18550	AT5G25400	AT5G39880
AT4G15950	AT4G22790	AT4G27490	AT4G31890	AT4G37280	AT5G03620	AT5G08110	AT5G13960	AT5G18580	AT5G25475	AT5G40040
AT4G16130	AT4G22860	AT4G27510	AT4G32420	AT4G37490	AT5G03680	AT5G08170	AT5G14380	AT5G18620	AT5G25510	AT5G40260
AT4G16340	AT4G22960	AT4G28190	AT4G32440	AT4G37740	AT5G03740	AT5G08210	AT5G14500	AT5G18750	AT5G25550	AT5G40480
AT4G16480	AT4G22970	AT4G28230	AT4G32551	AT4G37750	AT5G03790	AT5G08480	AT5G14610	AT5G19580	AT5G25570	AT5G40550
AT4G16620	AT4G23800	AT4G28280	AT4G32620	AT4G37840	AT5G03870	AT5G08565	AT5G14640	AT5G19600	AT5G25580	AT5G40640
AT4G16845	AT4G23860	AT4G28310	AT4G32710	AT4G38130	AT5G04110	AT5G08580	AT5G14670	AT5G19610	AT5G25590	AT5G40870
AT4G16970	AT4G23895	AT4G28430	AT4G32730	AT4G38230	AT5G04290	AT5G08630	AT5G14890	AT5G19640	AT5G25880	AT5G41870
AT4G17000	AT4G23900	AT4G28440	AT4G32830	AT4G38660	AT5G04460	AT5G09500	AT5G14990	AT5G20240	AT5G26060	AT5G42170
AT4G17483	AT4G24580	AT4G28470	AT4G32970	AT4G39110	AT5G04940	AT5G09550	AT5G15070	AT5G20540	AT5G26700	AT5G42700
AT4G18050	AT4G24610	AT4G28530	AT4G33130	AT4G39170	AT5G05080	AT5G09790	AT5G15170	AT5G20680	AT5G26710	AT5G42780
AT4G18465	AT4G24710	AT4G28680	AT4G33140	AT4G39550	AT5G05670	AT5G10260	AT5G15460	AT5G20710	AT5G26850	AT5G43020
AT4G18470	AT4G24790	AT4G28950	AT4G33400	AT4G39630	AT5G06050	AT5G10310	AT5G15510	AT5G20930	AT5G27240	AT5G43080
AT4G18550	AT4G25040	AT4G29030	AT4G34160	AT4G39860	AT5G06160	AT5G10400	AT5G15570	AT5G22430	AT5G27630	AT5G43810
AT4G18820	AT4G25120	AT4G29130	AT4G34340	AT4G40000	AT5G06590	AT5G10650	AT5G15610	AT5G22740	AT5G27680	AT5G43990
AT4G18920	AT4G25150	AT4G29170	AT4G34400	AT5G01370	AT5G06670	AT5G11020	AT5G15630	AT5G22760	AT5G27740	AT5G44180
AT4G18960	AT4G25240	AT4G29340	AT4G34590	AT5G01660	AT5G06780	AT5G11160	AT5G15800	AT5G22880	AT5G28290	AT5G44200
AT4G20050	AT4G25590	AT4G29360	AT4G34990	AT5G01770	AT5G06940	AT5G11510	AT5G16260	AT5G23420	AT5G28640	AT5G44300
AT4G20320	AT4G25950	AT4G29830	AT4G35010	AT5G01890	AT5G07180	AT5G11780	AT5G16505	AT5G23580	AT5G33300	AT5G44620
AT4G20400	AT4G26260	AT4G29940	AT4G35050	AT5G01910	AT5G07280	AT5G12080	AT5G16510	AT5G23720	AT5G35090	AT5G44630
AT4G20910	AT4G26390	AT4G30090	AT4G35500	AT5G02010	AT5G07420	AT5G12100	AT5G16690	AT5G23880	AT5G35520	AT5G45400
AT4G21060	AT4G26660	AT4G30130	AT4G35620	AT5G02140	AT5G07430	AT5G12270	AT5G16760	AT5G23910	AT5G35930	AT5G45560
AT4G21270	AT4G26760	AT4G30200	AT4G35650	AT5G02370	AT5G07510	AT5G12440	AT5G17070	AT5G23940	AT5G37010	AT5G45700
AT4G21430	AT4G26830	AT4G30520	AT4G35670	AT5G02420	AT5G07520	AT5G13000	AT5G17160	AT5G24330	AT5G37020	AT5G45760
AT4G21550	AT4G26840	AT4G30850	AT4G35700	AT5G02520	AT5G07540	AT5G13150	AT5G17410	AT5G24610	AT5G38070	AT5G45880
AT4G21820	AT4G26870	AT4G30860	AT4G35730	AT5G02550	AT5G07550	AT5G13290	AT5G17480	AT5G24750	AT5G38110	AT5G45960
AT4G22140	AT4G27060	AT4G30870	AT4G35905	AT5G03250	AT5G07560	AT5G13300	AT5G17620	AT5G24870	AT5G38690	AT5G46200
AT4G22250	AT4G27180	AT4G31360	AT4G36180	AT5G03415	AT5G07660	AT5G13350	AT5G17930	AT5G24970	AT5G38760	AT5G46280
AT4G22360	AT4G27240	AT4G31610	AT4G36260	AT5G03440	AT5G07680	AT5G13520	AT5G18000	AT5G25090	AT5G38880	AT5G46700

AT5G46770	AT5G51120	AT5G56740	AT5G61480	AT5G65120
AT5G46940	AT5G51350	AT5G56790	AT5G61605	AT5G65420
AT5G47000	AT5G51560	AT5G57130	AT5G61700	AT5G65440
AT5G47100	AT5G51590	AT5G57320	AT5G61720	AT5G65450
AT5G47160	AT5G51600	AT5G57410	AT5G61865	AT5G65460
AT5G47600	AT5G51660	AT5G57670	AT5G62230	AT5G66020
AT5G47750	AT5G51850	AT5G57810	AT5G62290	AT5G66150
AT5G48140	AT5G51940	AT5G57970	AT5G62310	AT5G66230
AT5G48170	AT5G52340	AT5G58230	AT5G62390	AT5G66560
AT5G48310	AT5G52510	AT5G58575	AT5G62410	AT5G66740
AT5G48360	AT5G52950	AT5G59040	AT5G62500	AT5G66750
AT5G48480	AT5G53190	AT5G59120	AT5G62550	AT5G66940
AT5G48500	AT5G53620	AT5G59370	AT5G62710	AT5G67100
AT5G48600	AT5G53820	AT5G59740	AT5G62750	AT5G67110
AT5G48650	AT5G53950	AT5G59810	AT5G62850	AT5G67130
AT5G48820	AT5G54570	AT5G59840	AT5G62960	AT5G67200
AT5G49010	AT5G54670	AT5G59845	AT5G63070	AT5G67260
AT5G49020	AT5G54870	AT5G59910	AT5G63135	AT5G67270
AT5G49100	AT5G55520	AT5G60020	AT5G63540	AT5G67320
AT5G49120	AT5G55660	AT5G60140	AT5G63920	AT5G67460
AT5G49180	AT5G55670	AT5G60150	AT5G63940	AT5G67550
AT5G49500	AT5G55720	AT5G60200	AT5G63950	ATMG00490
AT5G49550	AT5G55760	AT5G60210	AT5G63960	ATMG00500
AT5G49555	AT5G55820	AT5G60690	AT5G64080	
AT5G50010	AT5G55830	AT5G60720	AT5G64200	
AT5G50030	AT5G55950	AT5G60930	AT5G64610	
AT5G50330	AT5G56330	AT5G61120	AT5G64670	
AT5G50790	AT5G56510	AT5G61190	AT5G64690	
AT5G50930	AT5G56580	AT5G61430	AT5G64950	
AT5G51030	AT5G56590	AT5G61460	AT5G64980	

Supplementary Table S4 – C) Co-expressed genes of *ARF6-ARF8* as obtained from CORNET online database

AT1G01370	AT1G09600	AT1G14840	AT1G20410	AT1G28290	AT1G50660	AT1G62360	AT1G68400	AT1G77270	AT2G19330	AT2G28355
AT1G02060	AT1G09800	AT1G14900	AT1G21090	AT1G29900	AT1G50950	AT1G62450	AT1G68640	AT1G77470	AT2G19670	AT2G28550
AT1G02180	AT1G10030	AT1G15290	AT1G21480	AT1G29980	AT1G51060	AT1G62500	AT1G69120	AT1G78430	AT2G19770	AT2G28620
AT1G02190	AT1G10640	AT1G15460	AT1G21560	AT1G30330	AT1G51460	AT1G62870	AT1G69180	AT1G78770	AT2G20100	AT2G28790
AT1G02205	AT1G10780	AT1G15480	AT1G21690	AT1G30490	AT1G51670	AT1G62950	AT1G69400	AT1G79420	AT2G20240	AT2G29040
AT1G02730	AT1G10850	AT1G15660	AT1G21740	AT1G30520	AT1G52150	AT1G63100	AT1G69420	AT1G79720	AT2G20440	AT2G29890
AT1G02800	AT1G10930	AT1G15940	AT1G21810	AT1G30570	AT1G52310	AT1G63160	AT1G69910	AT1G79820	AT2G20870	AT2G29940
AT1G03170	AT1G10980	AT1G16070	AT1G21880	AT1G30690	AT1G52570	AT1G63470	AT1G69950	AT1G80070	AT2G22180	AT2G30290
AT1G03270	AT1G11130	AT1G16680	AT1G22110	AT1G31150	AT1G52580	AT1G63480	AT1G70210	AT1G80080	AT2G22610	AT2G30630
AT1G03620	AT1G11600	AT1G16705	AT1G22130	AT1G32240	AT1G52680	AT1G63640	AT1G70510	AT1G80370	AT2G22840	AT2G30650
AT1G03710	AT1G11730	AT1G16780	AT1G22730	AT1G32250	AT1G53160	AT1G63650	AT1G70830	AT1G80850	AT2G23360	AT2G30690
AT1G03780	AT1G11820	AT1G16900	AT1G22760	AT1G32730	AT1G53300	AT1G63680	AT1G70895	AT2G01210	AT2G23700	AT2G31270
AT1G04050	AT1G11850	AT1G17110	AT1G23380	AT1G33680	AT1G54180	AT1G63710	AT1G71680	AT2G01750	AT2G24230	AT2G31830
AT1G04090	AT1G12070	AT1G17140	AT1G23790	AT1G35490	AT1G54690	AT1G63850	AT1G72250	AT2G02170	AT2G24440	AT2G32410
AT1G04520	AT1G12330	AT1G17920	AT1G24070	AT1G35780	AT1G54840	AT1G64300	AT1G72260	AT2G04280	AT2G24490	AT2G32440
AT1G04760	AT1G12430	AT1G18040	AT1G24400	AT1G43190	AT1G55200	AT1G64450	AT1G72290	AT2G04675	AT2G24630	AT2G32670
AT1G05440	AT1G12570	AT1G18090	AT1G25320	AT1G44110	AT1G55360	AT1G64580	AT1G72410	AT2G05210	AT2G25060	AT2G32765
AT1G05470	AT1G13030	AT1G18250	AT1G25470	AT1G44760	AT1G56210	AT1G65010	AT1G72480	AT2G05790	AT2G25420	AT2G33330
AT1G05670	AT1G13220	AT1G18370	AT1G25472	AT1G44780	AT1G57820	AT1G65380	AT1G72670	AT2G06925	AT2G26180	AT2G33560
AT1G05950	AT1G13250	AT1G18450	AT1G25510	AT1G44900	AT1G58430	AT1G65470	AT1G73590	AT2G07170	AT2G26330	AT2G33620
AT1G06420	AT1G13560	AT1G18550	AT1G26190	AT1G47340	AT1G59540	AT1G65730	AT1G74690	AT2G13350	AT2G26730	AT2G33670
AT1G06950	AT1G13710	AT1G18650	AT1G26480	AT1G47980	AT1G59640	AT1G65900	AT1G74850	AT2G13680	AT2G26760	AT2G33690
AT1G07370	AT1G13730	AT1G19100	AT1G26540	AT1G48410	AT1G60060	AT1G66740	AT1G75090	AT2G16270	AT2G27040	AT2G33860
AT1G07850	AT1G13790	AT1G19780	AT1G27120	AT1G49430	AT1G60560	AT1G66850	AT1G75150	AT2G16440	AT2G27228	AT2G34650
AT1G08560	AT1G14180	AT1G19830	AT1G27190	AT1G49480	AT1G60800	AT1G66940	AT1G75240	AT2G16640	AT2G27230	AT2G35310
AT1G08730	AT1G14350	AT1G19850	AT1G27360	AT1G49580	AT1G60860	AT1G67040	AT1G76310	AT2G16780	AT2G27990	AT2G35340
AT1G09160	AT1G14440	AT1G19890	AT1G27370	AT1G49730	AT1G61010	AT1G67630	AT1G76540	AT2G17950	AT2G28070	AT2G35620
AT1G09450	AT1G14510	AT1G20230	AT1G28270	AT1G50310	AT1G61110	AT1G68220	AT1G76630	AT2G19170	AT2G28100	AT2G36010

AT2G36020	AT2G45190	AT3G07820	AT3G17350	AT3G24150	AT3G49660	AT3G57860	AT4G01730	AT4G16442	AT4G26840	AT4G34980
AT2G36190	AT2G45340	AT3G08680	AT3G17360	AT3G24495	AT3G49670	AT3G57920	AT4G02150	AT4G16590	AT4G27060	AT4G35010
AT2G36350	AT2G45590	AT3G09070	AT3G17630	AT3G24630	AT3G50070	AT3G58160	AT4G02290	AT4G17240	AT4G28280	AT4G35050
AT2G36400	AT2G45600	AT3G09670	AT3G17680	AT3G24660	AT3G50620	AT3G59420	AT4G02680	AT4G18390	AT4G28680	AT4G35280
AT2G36660	AT2G45650	AT3G09930	AT3G18000	AT3G24810	AT3G50780	AT3G60660	AT4G02800	AT4G18820	AT4G28950	AT4G35700
AT2G36885	AT2G45700	AT3G10140	AT3G18050	AT3G25165	AT3G50890	AT3G60740	AT4G03100	AT4G18920	AT4G29360	AT4G35730
AT2G37080	AT2G47050	AT3G10410	AT3G18524	AT3G25500	AT3G51280	AT3G61250	AT4G03190	AT4G20160	AT4G30090	AT4G36180
AT2G37300	AT2G47230	AT3G10540	AT3G18570	AT3G25670	AT3G51490	AT3G61310	AT4G03210	AT4G20400	AT4G30130	AT4G36360
AT2G37390	AT2G47610	AT3G10570	AT3G18800	AT3G26140	AT3G51720	AT3G61360	AT4G04080	AT4G20910	AT4G30520	AT4G36520
AT2G37420	AT2G47900	AT3G11000	AT3G18850	AT3G26540	AT3G51740	AT3G61610	AT4G04890	AT4G21070	AT4G30990	AT4G37110
AT2G38770	AT3G01020	AT3G12390	AT3G19120	AT3G26932	AT3G52110	AT3G61670	AT4G04930	AT4G21550	AT4G31360	AT4G37210
AT2G39090	AT3G01240	AT3G12550	AT3G19570	AT3G28500	AT3G52620	AT3G61830	AT4G05190	AT4G21750	AT4G31590	AT4G37640
AT2G40030	AT3G01250	AT3G12870	AT3G20010	AT3G28730	AT3G52890	AT3G62060	AT4G08150	AT4G21820	AT4G31610	AT4G37740
AT2G40070	AT3G01270	AT3G13065	AT3G20020	AT3G28790	AT3G52940	AT3G62170	AT4G08670	AT4G22860	AT4G31805	AT4G37750
AT2G40460	AT3G02110	AT3G13160	AT3G20180	AT3G42170	AT3G53080	AT3G62230	AT4G09160	AT4G23440	AT4G31820	AT4G37840
AT2G40640	AT3G02210	AT3G13190	AT3G20260	AT3G42640	AT3G53140	AT3G62300	AT4G10270	AT4G23620	AT4G31840	AT4G38050
AT2G41210	AT3G02300	AT3G13400	AT3G20670	AT3G42670	AT3G53190	AT3G62640	AT4G10850	AT4G23740	AT4G31880	AT4G38150
AT2G41550	AT3G02310	AT3G13510	AT3G21100	AT3G43920	AT3G53760	AT3G63130	AT4G11030	AT4G23750	AT4G31890	AT4G38660
AT2G41890	AT3G02890	AT3G13674	AT3G21480	AT3G44230	AT3G54080	AT3G63200	AT4G11760	AT4G23800	AT4G32551	AT4G39010
AT2G42120	AT3G04470	AT3G13690	AT3G21970	AT3G44600	AT3G54350	AT3G63240	AT4G12130	AT4G23940	AT4G32605	AT4G39170
AT2G42200	AT3G05180	AT3G13960	AT3G22520	AT3G45980	AT3G54430	AT3G63290	AT4G13640	AT4G24670	AT4G32620	AT4G39480
AT2G42800	AT3G05470	AT3G14190	AT3G22780	AT3G46520	AT3G54630	AT3G63300	AT4G14200	AT4G24710	AT4G32710	AT4G39550
AT2G43230	AT3G05720	AT3G14240	AT3G22790	AT3G46750	AT3G54650	AT4G00350	AT4G14695	AT4G24790	AT4G33400	AT4G39900
AT2G43800	AT3G06030	AT3G15030	AT3G23210	AT3G47440	AT3G54670	AT4G00370	AT4G14770	AT4G25150	AT4G34160	AT5G01075
AT2G43900	AT3G06130	AT3G15095	AT3G23350	AT3G48425	AT3G54750	AT4G00480	AT4G15110	AT4G25240	AT4G34400	AT5G01370
AT2G44150	AT3G06160	AT3G15270	AT3G23670	AT3G48480	AT3G54790	AT4G00870	AT4G15140	AT4G25950	AT4G34440	AT5G01590
AT2G44190	AT3G06220	AT3G15550	AT3G23830	AT3G48710	AT3G55110	AT4G01470	AT4G15830	AT4G26660	AT4G34610	AT5G01660
AT2G44830	AT3G07540	AT3G15650	AT3G23890	AT3G49250	AT3G57830	AT4G01690	AT4G16340	AT4G26760	AT4G34730	AT5G01890

AT5G02030	AT5G14210	AT5G26850	AT5G46770	AT5G58230
AT5G02190	AT5G14380	AT5G27630	AT5G46940	AT5G58550
AT5G02720	AT5G14600	AT5G28640	AT5G47500	AT5G58600
AT5G03250	AT5G15510	AT5G35090	AT5G47600	AT5G59010
AT5G04940	AT5G15570	AT5G35670	AT5G48360	AT5G60210
AT5G05550	AT5G15580	AT5G35750	AT5G48480	AT5G60690
AT5G05610	AT5G15800	AT5G37010	AT5G49020	AT5G61000
AT5G06430	AT5G16100	AT5G37020	AT5G49160	AT5G61480
AT5G06970	AT5G16240	AT5G37300	AT5G49330	AT5G61720
AT5G07180	AT5G16290	AT5G37890	AT5G50030	AT5G62230
AT5G07560	AT5G16690	AT5G38070	AT5G51030	AT5G62710
AT5G08000	AT5G17160	AT5G38150	AT5G51350	AT5G62850
AT5G08550	AT5G18750	AT5G38880	AT5G51550	AT5G63920
AT5G08580	AT5G18910	AT5G39410	AT5G51560	AT5G63960
AT5G09550	AT5G19580	AT5G39840	AT5G51590	AT5G64980
AT5G09790	AT5G19610	AT5G40870	AT5G52360	AT5G65410
AT5G10310	AT5G20740	AT5G41880	AT5G53210	AT5G65640
AT5G10560	AT5G23300	AT5G42620	AT5G53620	AT5G65820
AT5G11130	AT5G23400	AT5G42920	AT5G54148	AT5G66560
AT5G11550	AT5G23880	AT5G43020	AT5G55340	AT5G66740
AT5G12050	AT5G23910	AT5G43810	AT5G55520	AT5G66770
AT5G12080	AT5G23940	AT5G43990	AT5G55720	AT5G66940
AT5G12440	AT5G25090	AT5G44300	AT5G56220	AT5G67100
AT5G12900	AT5G25400	AT5G44560	AT5G57130	AT5G67110
AT5G13000	AT5G25475	AT5G44620	AT5G57320	AT5G67130
AT5G13290	AT5G25550	AT5G44630	AT5G57590	AT5G67200
AT5G13300	AT5G25590	AT5G45960	AT5G57690	AT5G67270
AT5G13350	AT5G26670	AT5G46570	AT5G57810	
AT5G13840	AT5G26710	AT5G46700	AT5G58050	
