# Supplementary material for: The rolB‐transgenic Nicotiana tabacum plants exhibit upregulated ARF7 and ARF19 gene expression
Source: Plant Direct. 2022 Jun 18;6(6):e414. doi: 10.1002/pld3.414 (PMC9219009; doi:10.1002/pld3.414)
Supplement: Supplementary file 13 — Table S6 Functional categories represented by AuxRE motif possessing co‐expressed genes of A) ARF7‐ARF19; B) ARF5 and C) ARF6‐ARF8 analyzed by BiNGO. [file PLD3-6-e414-s012.pdf]

Supplementary Table S6 – A) Functional categories represented by AuxRE motif possessing co-expressed genes of *ARF7-ARF19* analyzed by BiNGO

<table>
<tr><th>GO-ID</th><th>p-value</th><th>corr p-value</th><th>x</th><th>n</th><th>X</th><th>N</th><th>Description</th><th>Genes in test set</th></tr>
<tr><td>22622</td><td>7.51E-09</td><td>2.36E-06</td><td>12.00</td><td>230</td><td>126</td><td>22304</td><td>root system development</td><td>AT4G29040|AT5G12330|AT1G19220|AT3G62100|AT5G03540|AT5G58270|AT2G38120|AT1G36160|AT1G19850|AT5G20730|AT5G43900|AT5G16910</td></tr>
<tr><td>48364</td><td>7.51E-09</td><td>2.36E-06</td><td>12.00</td><td>230</td><td>126</td><td>22304</td><td>root development</td><td>AT4G29040|AT5G12330|AT1G19220|AT3G62100|AT5G03540|AT5G58270|AT2G38120|AT1G36160|AT1G19850|AT5G20730|AT5G43900|AT5G16910</td></tr>
<tr><td>48513</td><td>2.26E-08</td><td>3.64E-06</td><td>19.00</td><td>719</td><td>126</td><td>22304</td><td>organ development</td><td>AT1G79280|AT5G19530|AT2G38120|AT5G60690|AT1G19850|AT5G16910|AT3G15880|AT2G46410|AT2G45420|AT3G63440|AT4G29040|AT5G12330|AT1G19220|AT3G62100|AT5G03540|AT5G58270|AT1G36160|AT5G20730|AT5G43900</td></tr>
<tr><td>48731</td><td>2.31E-08</td><td>3.64E-06</td><td>19.00</td><td>720</td><td>126</td><td>22304</td><td>system development</td><td>AT1G79280|AT5G19530|AT2G38120|AT5G60690|AT1G19850|AT5G16910|AT3G15880|AT2G46410|AT2G45420|AT3G63440|AT4G29040|AT5G12330|AT1G19220|AT3G62100|AT5G03540|AT5G58270|AT1G36160|AT5G20730|AT5G43900</td></tr>
<tr><td>48856</td><td>2.21E-07</td><td>2.45E-05</td><td>25.00</td><td>1392</td><td>126</td><td>22304</td><td>anatomical structure development</td><td>AT5G19530|AT5G64340|AT3G15880|AT2G46410|AT2G45420|AT3G63440|AT5G12330|AT5G03540|AT5G58270|AT5G43900|AT1G79280|AT2G38120|AT5G60690|AT1G19850|AT5G16910|AT1G49320|AT4G29040|AT4G37590|AT1G19220|AT3G62100|AT5G04240|AT5G37020|AT1G36160|AT5G20730|AT2G03150</td></tr>
<tr><td>32502</td><td>2.50E-07</td><td>2.45E-05</td><td>29.00</td><td>1820</td><td>126</td><td>22304</td><td>developmental process</td><td>AT4G01550|AT5G19530|AT5G64340|AT3G15880|AT2G46410|AT2G45420|AT3G63440|AT5G12330|AT5G03540|AT5G58270|AT5G50230|AT5G43900|AT1G79280|AT2G38120|AT3G61850|AT5G60690|AT1G19850|AT5G16910|AT1G49320|AT3G12680|AT4G29040|AT4G37590|AT1G19220|AT3G62100|AT5G04240|AT5G37020|AT1G36160|AT5G20730|AT2G03150</td></tr>
<tr><td>9888</td><td>2.72E-07</td><td>2.45E-05</td><td>11.00</td><td>260</td><td>126</td><td>22304</td><td>tissue development</td><td>AT3G15880|AT2G46410|AT2G45420|AT4G29040|AT5G19530|AT2G38120|AT5G60690|AT1G36160|AT1G19850|AT5G43900|AT5G16910</td></tr>
<tr><td>32501</td><td>1.09E-06</td><td>8.60E-05</td><td>27.00</td><td>1732</td><td>126</td><td>22304</td><td>multicellular organismal process</td><td>AT4G01550|AT5G19530|AT3G15880|AT2G46410|AT2G45420|AT3G63440|AT5G12330|AT5G03540|AT5G58270|AT5G50230|AT5G43900|AT1G79280|AT1G09570|AT2G38120|AT3G61850|AT5G60690|AT1G19850|AT5G16910|AT1G49320|AT4G29040|AT4G37590|AT1G19220|AT3G62100|AT5G37020|AT1G36160|AT5G20730|AT2G03150</td></tr>
</table>

<table>
<tr><th></th><th></th><th></th><th></th><th></th><th></th><th></th><th></th><th></th></tr>
<tr><td>7275</td><td>1.56E-06</td><td>1.09E-04</td><td>26.00</td><td>1655</td><td>126</td><td>22304</td><td>multicellular organismal development</td><td>AT4G01550|AT5G19530|AT3G15880|AT2G46410|AT2G45420|AT3G63440|AT5G12330|AT5G03540|AT5G58270|AT5G50230|AT5G43900|AT1G79280|AT2G38120|AT3G61850|AT5G60690|AT1G19850|AT5G16910|AT1G49320|AT4G29040|AT4G37590|AT1G19220|AT3G62100|AT5G37020|AT1G36160|AT5G20730|AT2G03150</td></tr>
<tr><td>65007</td><td>2.03E-06</td><td>1.28E-04</td><td>39.00</td><td>3243</td><td>126</td><td>22304</td><td>biological regulation</td><td>AT5G22760|AT4G01550|AT1G68810|AT5G23450|AT3G49760|AT4G04920|AT3G04030|AT5G19530|AT5G64340|AT2G46410|AT2G21230|AT3G60030|AT3G63440|AT5G07920|AT5G58270|AT1G20110|AT5G03520|AT1G02120|AT4G14580|AT5G43900|AT4G32010|AT1G79280|AT3G49350|AT1G09570|AT2G38120|AT1G72840|AT5G48560|AT5G60690|AT1G70920|AT5G16910|AT5G53570|AT4G29040|AT4G08180|AT5G04240|AT1G20160|AT5G37020|AT5G58380|AT5G20730|AT4G33430</td></tr>
<tr><td>10386</td><td>3.48E-06</td><td>1.99E-04</td><td>3.00</td><td>6</td><td>126</td><td>22304</td><td>lateral root primordium development</td><td>AT4G29040|AT1G19220|AT5G20730</td></tr>
<tr><td>48527</td><td>1.61E-05</td><td>8.46E-04</td><td>5.00</td><td>56</td><td>126</td><td>22304</td><td>lateral root development</td><td>AT4G29040|AT1G19220|AT5G03540|AT2G38120|AT5G20730</td></tr>
<tr><td>48528</td><td>2.66E-05</td><td>1.29E-03</td><td>5.00</td><td>62</td><td>126</td><td>22304</td><td>post-embryonic root development</td><td>AT4G29040|AT1G19220|AT5G03540|AT2G38120|AT5G20730</td></tr>
<tr><td>50794</td><td>3.15E-05</td><td>1.42E-03</td><td>30.00</td><td>2448</td><td>126</td><td>22304</td><td>regulation of cellular process</td><td>AT5G22760|AT4G01550|AT1G68810|AT5G23450|AT3G49760|AT3G04030|AT2G46410|AT2G21230|AT3G60030|AT5G07920|AT5G58270|AT1G20110|AT5G03520|AT1G02120|AT4G14580|AT4G32010|AT1G79280|AT3G49350|AT1G09570|AT1G72840|AT5G48560|AT5G60690|AT1G70920|AT5G53570|AT4G29040|AT4G08180|AT5G04240|AT5G37020|AT5G58380|AT5G20730</td></tr>
<tr><td>9653</td><td>3.63E-05</td><td>1.52E-03</td><td>12.00</td><td>515</td><td>126</td><td>22304</td><td>anatomical structure morphogenesis</td><td>AT3G15880|AT3G63440|AT4G29040|AT5G04240|AT5G19530|AT5G03540|AT2G38120|AT5G64340|AT5G60690|AT1G36160|AT5G43900|AT5G16910</td></tr>
<tr><td>9791</td><td>3.92E-05</td><td>1.54E-03</td><td>16.00</td><td>884</td><td>126</td><td>22304</td><td>post-embryonic development</td><td>AT1G79280|AT2G38120|AT3G61850|AT1G19850|AT1G49320|AT3G15880|AT3G63440|AT4G29040|AT4G37590|AT1G19220|AT3G62100|AT5G03540|AT5G37020|AT1G36160|AT5G20730|AT2G03150</td></tr>
<tr><td>9933</td><td>5.55E-05</td><td>2.06E-03</td><td>4.00</td><td>37</td><td>126</td><td>22304</td><td>meristem structural organization</td><td>AT3G15880|AT4G29040|AT5G60690|AT1G36160</td></tr>
<tr><td>9630</td><td>6.18E-05</td><td>2.16E-03</td><td>4.00</td><td>38</td><td>126</td><td>22304</td><td>gravitropism</td><td>AT1G09570|AT3G62100|AT2G38120|AT5G20730</td></tr>
</table>

50896	6.68E-05	2.21E-03	35.00	3207	126	22304	response to stimulus	AT4G04920IAT3G21630IAT2G32250IAT2G46410IAT3G14430IAT5G12330IAT4G18880IAT5G58270IAT2G24040IAT1G02120IAT3G04880IAT3G53990IAT1G09570IAT2G38120IAT3G61850IAT1G72840IAT1G19850IAT1G66480IAT5G16910IAT5G10520IAT5G11670IAT2G37280IAT4G29040IAT3G60240IAT4G37590IAT1G19220IAT3G62100IAT5G04240IAT2G21620IAT3G12360IAT5G37020IAT1G21610IAT1G36160IAT5G20730IAT4G33430
50790	7.81E-05	2.46E-03	6.00	125	126	22304	regulation of catalytic activity	AT3G49350IAT5G53570IAT5G23450IAT1G20160IAT5G07920IAT5G58270
48532	9.20E-05	2.76E-03	4.00	42	126	22304	anatomical structure arrangement	AT3G15880IAT4G29040IAT5G60690IAT1G36160
9629	1.11E-04	3.17E-03	4.00	44	126	22304	response to gravity	AT1G09570IAT3G62100IAT2G38120IAT5G20730
65009	1.24E-04	3.41E-03	6.00	136	126	22304	regulation of molecular function	AT3G49350IAT5G53570IAT5G23450IAT1G20160IAT5G07920IAT5G58270
50789	1.37E-04	3.60E-03	31.00	2783	126	22304	regulation of biological process	AT5G22760IAT4G01550IAT1G68810IAT5G23450IAT3G49760IAT4G04920IAT3G04030IAT2G46410IAT2G21230IAT3G60030IAT5G07920IAT5G58270IAT1G20110IAT5G03520IAT1G02120IAT4G14580IAT4G32010IAT1G79280IAT3G49350IAT1G09570IAT1G72840IAT5G48560IAT5G60690IAT1G70920IAT5G53570IAT4G29040IAT4G08180IAT5G04240IAT5G37020IAT5G58380IAT5G20730
31323	1.51E-04	3.78E-03	22.00	1664	126	22304	regulation of cellular metabolic process	AT5G22760IAT1G79280IAT3G49350IAT4G01550IAT1G68810IAT5G23450IAT3G49760IAT1G09570IAT3G04030IAT5G48560IAT5G60690IAT1G70920IAT2G46410IAT2G21230IAT5G53570IAT3G60030IAT5G04240IAT5G07920IAT5G37020IAT5G58270IAT5G20730IAT4G32010
9606	1.56E-04	3.78E-03	4.00	48	126	22304	tropism	AT1G09570IAT3G62100IAT2G38120IAT5G20730
48869	1.85E-04	4.31E-03	10.00	435	126	22304	cellular developmental process	AT2G46410IAT3G12680IAT4G29040IAT5G04240IAT5G19530IAT2G38120IAT5G64340IAT5G60690IAT5G43900IAT5G16910
48507	2.05E-04	4.56E-03	5.00	95	126	22304	meristem development	AT3G15880IAT4G29040IAT5G60690IAT1G36160IAT1G19850
19222	2.12E-04	4.56E-03	23.00	1825	126	22304	regulation of metabolic process	AT5G22760IAT1G79280IAT3G49350IAT4G01550IAT1G68810IAT5G23450IAT3G49760IAT4G04920IAT1G09570IAT3G04030IAT5G48560IAT5G60690IAT1G70920IAT2G46410IAT2G21230IAT5G53570IAT3G60030IAT5G04240IAT5G07920IAT5G37020IAT5G58270IAT5G20730IAT4G32010
10014	2.17E-04	4.56E-03	3.00	21	126	22304	meristem initiation	AT3G15880IAT5G60690IAT1G36160
10015	2.49E-04	5.05E-03	5.00	99	126	22304	root morphogenesis	AT4G29040IAT5G03540IAT2G38120IAT5G43900IAT5G16910
30154	2.84E-04	5.60E-03	8.00	296	126	22304	cell differentiation	AT2G46410IAT3G12680IAT4G29040IAT5G19530IAT2G38120IAT5G60690IAT5G43900IAT5G16910

48586	3.13E-04	5.98E-03	2.00	5	126	22304	lation of long-day photoperiodism, flow	AT4G04920IAT5G04240
6796	4.22E-04	7.69E-03	15.00	979	126	22304	phosphate metabolic process	AT3G59700IAT3G55450IAT5G27840IAT2G02800IAT1G18160IAT5G63930IAT5G05160IAT5G53320I AT2G45910IAT5G03320IAT3G07690IAT5G58380IAT5G38560IAT4G14580IAT1G03740
6793	4.27E-04	7.69E-03	15.00	980	126	22304	phosphorus metabolic process	AT3G59700IAT3G55450IAT5G27840IAT2G02800IAT1G18160IAT5G63930IAT5G05160IAT5G53320I AT2G45910IAT5G03320IAT3G07690IAT5G58380IAT5G38560IAT4G14580IAT1G03740
9628	9.06E-04	1.59E-02	16.00	1168	126	22304	response to abiotic stimulus	AT3G04880IAT3G53990IAT4G04920IAT1G09570IAT2G38120IAT3G61850IAT1G66480IAT2G32250I AT5G16910IAT4G29040IAT4G37590IAT3G62100IAT2G21620IAT3G12360IAT2G24040IAT5G20730
6468	9.69E-04	1.65E-02	13.00	842	126	22304	protein amino acid phosphorylation	AT3G59700IAT3G55450IAT2G02800IAT1G18160IAT5G63930IAT5G05160IAT5G53320IAT2G45910I AT5G03320IAT5G58380IAT5G38560IAT4G14580IAT1G03740
48569	1.03E-03	1.65E-02	6.00	202	126	22304	post-embryonic organ development	AT1G79280IAT4G29040IAT1G19220IAT5G03540IAT2G38120IAT5G20730
48589	1.05E-03	1.65E-02	6.00	203	126	22304	developmental growth	AT4G29040IAT5G04240IAT5G19530IAT5G64340IAT5G43900IAT5G16910
40007	1.06E-03	1.65E-02	7.00	279	126	22304	growth	AT4G29040IAT5G04240IAT5G19530IAT5G64340IAT4G33430IAT5G43900IAT5G16910
51171	1.08E-03	1.65E-02	19.00	1545	126	22304	ation of nitrogen compound metabolic pr	AT5G22760IAT3G49350IAT4G01550IAT1G68810IAT3G49760IAT1G09570IAT3G04030IAT5G48560I AT5G60690IAT1G70920IAT2G46410IAT2G21230IAT5G53570IAT3G60030IAT5G04240IAT5G37020I AT5G58270IAT5G20730IAT4G32010
9733	1.13E-03	1.69E-02	7.00	282	126	22304	response to auxin stimulus	AT5G12330IAT1G19220IAT3G62100IAT5G37020IAT2G38120IAT1G19850IAT5G20730
43687	1.30E-03	1.90E-02	15.00	1093	126	22304	post-translational protein modification	AT3G59700IAT3G55450IAT5G27840IAT2G02800IAT1G18160IAT5G63930IAT5G05160IAT5G53320I AT2G45910IAT5G04240IAT5G03320IAT5G58380IAT5G38560IAT4G14580IAT1G03740
42221	1.45E-03	2.08E-02	20.00	1710	126	22304	response to chemical stimulus	AT1G09570IAT2G38120IAT1G19850IAT3G21630IAT2G46410IAT3G14430IAT5G11670IAT2G37280I AT4G29040IAT5G12330IAT1G19220IAT3G62100IAT5G04240IAT2G21620IAT5G37020IAT4G18880I AT5G58270IAT1G36160IAT5G20730IAT4G33430
48469	1.60E-03	2.10E-02	3.00	41	126	22304	cell maturation	AT2G38120IAT5G43900IAT5G16910
48764	1.60E-03	2.10E-02	3.00	41	126	22304	trichoblast maturation	AT2G38120IAT5G43900IAT5G16910
48765	1.60E-03	2.10E-02	3.00	41	126	22304	root hair cell differentiation	AT2G38120IAT5G43900IAT5G16910

80090	1.66E-03	2.10E-02	19.00	1604	126	22304	regulation of primary metabolic process	AT5G22760IAT1G79280IAT3G49350IAT4G01550IAT1G68810IAT3G49760IAT1G09570IAT3G04030I<br>AT5G48560IAT5G60690IAT1G70920IAT2G46410IAT2G21230IAT5G53570IAT3G60030IAT5G04240I<br>AT5G37020IAT5G20730IAT4G32010
7205	1.68E-03	2.10E-02	2.00	11	126	22304	C activity by G-protein coupled recepto	AT5G23450IAT5G07920
32147	1.68E-03	2.10E-02	2.00	11	126	22304	activation of protein kinase activity	AT5G23450IAT5G07920
6464	1.70E-03	2.10E-02	16.00	1241	126	22304	protein modification process	AT4G24990IAT3G59700IAT3G55450IAT5G27840IAT2G02800IAT1G18160IAT5G63930IAT5G05160I<br>AT5G53320IAT2G45910IAT5G04240IAT5G03320IAT5G58380IAT5G38560IAT4G14580IAT1G03740
16310	1.94E-03	2.30E-02	13.00	910	126	22304	phosphorylation	AT3G59700IAT3G55450IAT2G02800IAT1G18160IAT5G63930IAT5G05160IAT5G53320IAT2G45910I<br>AT5G03320IAT5G58380IAT5G38560IAT4G14580IAT1G03740
51239	1.94E-03	2.30E-02	6.00	229	126	22304	ulation of multicellular organismal proc	AT2G46410IAT1G79280IAT4G04920IAT5G04240IAT5G58270IAT4G32010
10054	1.97E-03	2.30E-02	3.00	44	126	22304	trichoblast differentiation	AT2G38120IAT5G43900IAT5G16910
9638	2.01E-03	2.31E-02	2.00	12	126	22304	phototropism	AT1G09570IAT5G20730
10033	2.19E-03	2.47E-02	14.00	1037	126	22304	response to organic substance	AT2G38120IAT1G19850IAT3G21630IAT2G46410IAT4G29040IAT5G12330IAT1G19220IAT3G62100I<br>AT5G04240IAT5G37020IAT4G18880IAT1G36160IAT5G20730IAT4G33430
19219	2.34E-03	2.53E-02	18.00	1527	126	22304	se, nucleoside, nucleotide and nucleic a	AT5G22760IAT3G49350IAT4G01550IAT1G68810IAT3G49760IAT1G09570IAT3G04030IAT5G48560I<br>AT5G60690IAT1G70920IAT2G46410IAT2G21230IAT5G53570IAT3G60030IAT5G04240IAT5G37020I<br>AT5G20730IAT4G32010
48829	2.37E-03	2.53E-02	2.00	13	126	22304	root cap development	AT4G29040IAT2G38120
10072	2.37E-03	2.53E-02	2.00	13	126	22304	mary shoot apical meristem specificati	AT3G15880IAT1G36160
16049	2.46E-03	2.58E-02	6.00	240	126	22304	cell growth	AT5G04240IAT5G19530IAT5G64340IAT4G33430IAT5G43900IAT5G16910
45860	2.76E-03	2.76E-02	2.00	14	126	22304	ositive regulation of protein kinase activi	AT5G23450IAT5G07920
10089	2.76E-03	2.76E-02	2.00	14	126	22304	xylem development	AT2G45420IAT5G19530
33674	2.76E-03	2.76E-02	2.00	14	126	22304	positive regulation of kinase activity	AT5G23450IAT5G07920
9637	2.84E-03	2.80E-02	3.00	50	126	22304	response to blue light	AT1G09570IAT5G20730IAT1G66480
21700	3.18E-03	3.06E-02	3.00	52	126	22304	developmental maturation	AT2G38120IAT5G43900IAT5G16910
8361	3.25E-03	3.06E-02	6.00	254	126	22304	regulation of cell size	AT5G04240IAT5G19530IAT5G64340IAT4G33430IAT5G43900IAT5G16910
32535	3.31E-03	3.06E-02	6.00	255	126	22304	regulation of cellular component size	AT5G04240IAT5G19530IAT5G64340IAT4G33430IAT5G43900IAT5G16910
90066	3.31E-03	3.06E-02	6.00	255	126	22304	regulation of anatomical structure size	AT5G04240IAT5G19530IAT5G64340IAT4G33430IAT5G43900IAT5G16910
10053	3.36E-03	3.06E-02	3.00	53	126	22304	root epidermal cell differentiation	AT2G38120IAT5G43900IAT5G16910
51347	4.07E-03	3.67E-02	2.00	17	126	22304	ositive regulation of transferase activity	AT5G23450IAT5G07920

7186	4.57E-03	3.88E-02	2.00	18	126	22304	ein coupled receptor protein signaling p	AT5G23450|AT5G07920
51640	4.57E-03	3.88E-02	2.00	18	126	22304	organelle localization	AT1G66480|AT5G43900
32313	4.57E-03	3.88E-02	2.00	18	126	22304	regulation of Rab GTPase activity	AT3G49350|AT5G53570
32483	4.57E-03	3.88E-02	2.00	18	126	22304	gulation of Rab protein signal transducti	AT3G49350|AT5G53570
50793	4.61E-03	3.88E-02	6.00	273	126	22304	regulation of developmental process	AT2G46410|AT1G79280|AT4G29040|AT4G04920|AT5G04240|AT4G32010
43412	4.77E-03	3.91E-02	16.00	1378	126	22304	macromolecule modification	AT4G24990|AT3G59700|AT3G55450|AT5G27840|AT2G02800|AT1G18160|AT5G63930|AT5G05160|AT5G53320|AT2G45910|AT5G04240|AT5G03320|AT5G58380|AT5G38560|AT4G14580|AT1G03740
7166	4.96E-03	3.91E-02	4.00	121	126	22304	l surface receptor linked signaling pathw	AT5G05160|AT5G23450|AT5G53320|AT5G07920
6665	5.08E-03	3.91E-02	2.00	19	126	22304	sphingolipid metabolic process	AT3G24180|AT5G23450
23052	5.24E-03	3.91E-02	13.00	1023	126	22304	signaling	AT5G23450|AT1G09570|AT1G72840|AT5G05160|AT5G53320|AT4G08180|AT5G07920|AT5G58380|AT1G20110|AT5G03520|AT5G20730|AT4G14580|AT4G33430
9913	5.26E-03	3.91E-02	4.00	123	126	22304	epidermal cell differentiation	AT2G46410|AT2G38120|AT5G43900|AT5G16910
7398	5.56E-03	3.91E-02	4.00	125	126	22304	ectoderm development	AT2G46410|AT2G38120|AT5G43900|AT5G16910
8544	5.56E-03	3.91E-02	4.00	125	126	22304	epidermis development	AT2G46410|AT2G38120|AT5G43900|AT5G16910
48508	5.63E-03	3.91E-02	2.00	20	126	22304	embryonic meristem development	AT3G15880|AT1G36160
40014	5.65E-03	3.91E-02	1.00	1	126	22304	gulation of multicellular organism grow	AT5G58270
48227	5.65E-03	3.91E-02	1.00	1	126	22304	lasma membrane to endosome transpo	AT2G44100
35266	5.65E-03	3.91E-02	1.00	1	126	22304	meristem growth	AT4G29040
33233	5.65E-03	3.91E-02	1.00	1	126	22304	regulation of protein sumoylation	AT1G79280
33234	5.65E-03	3.91E-02	1.00	1	126	22304	egative regulation of protein sumoylatic	AT1G79280
48759	5.65E-03	3.91E-02	1.00	1	126	22304	vessel member cell differentiation	AT5G19530
32491	5.65E-03	3.91E-02	1.00	1	126	22304	detection of molecule of fungal origin	AT3G21630
10203	5.65E-03	3.91E-02	1.00	1	126	22304	ponse to very low fluence red light stimu	AT1G09570
31326	5.99E-03	4.06E-02	17.00	1540	126	22304	egulation of cellular biosynthetic proces	AT5G22760|AT4G01550|AT1G68810|AT3G49760|AT1G09570|AT3G04030|AT5G48560|AT5G60690|AT1G70920|AT2G46410|AT2G21230|AT3G60030|AT5G04240|AT5G37020|AT5G58270|AT5G20730|AT4G32010
9889	5.99E-03	4.06E-02	17.00	1540	126	22304	regulation of biosynthetic process	AT5G22760|AT4G01550|AT1G68810|AT3G49760|AT1G09570|AT3G04030|AT5G48560|AT5G60690|AT1G70920|AT2G46410|AT2G21230|AT3G60030|AT5G04240|AT5G37020|AT5G58270|AT5G20730|AT4G32010
60255	6.55E-03	4.39E-02	18.00	1685	126	22304	lation of macromolecule metabolic pro	AT5G22760|AT1G79280|AT4G01550|AT1G68810|AT3G49760|AT4G04920|AT1G09570|AT3G04030|AT5G48560|AT5G60690|AT1G70920|AT2G46410|AT2G21230|AT3G60030|AT5G04240|AT5G37020|AT5G20730|AT4G32010
48367	7.22E-03	4.79E-02	6.00	300	126	22304	shoot development	AT3G15880|AT3G63440|AT4G29040|AT1G19220|AT1G36160|AT5G20730

Supplementary Table S6 – B) Functional categories represented by AuxRE motif possessing co-expressed genes of *ARF5* analyzed by BiNGO

<table>
<tr><th>GO-ID</th><th>p-value</th><th>corr p-value</th><th>x</th><th>n</th><th>X</th><th>N</th><th>Description</th><th>Genes in test set</th></tr>
<tr><td>51276</td><td>5.64E-15</td><td>3.20E-12</td><td>29</td><td>210</td><td>502</td><td>22304</td><td>chromosome organization</td><td>AT3G54670|AT1G63020|AT5G22880|AT1G04050|AT5G18620|AT1G57820|AT2G35160|AT3G20475|AT5G13960|AT4G38130|AT4G37280|AT2G40550|AT4G30870|AT5G58230|AT3G02680|AT4G29830|AT2G31970|AT1G07790|AT3G53730|AT5G43990|AT3G20670|AT5G61460|AT5G67320|AT5G10400|AT5G64610|AT1G76540|AT1G18450|AT5G62410|AT2G23380</td></tr>
<tr><td>16043</td><td>7.04E-15</td><td>3.20E-12</td><td>63</td><td>935</td><td>502</td><td>22304</td><td>cellular component organization</td><td>AT5G67270|AT5G14380|AT5G18580|AT1G63020|AT5G22880|AT3G23670|AT2G46180|AT1G04050|AT5G18620|AT1G57820|AT2G35160|AT2G34790|AT3G20475|AT5G13960|AT4G38130|AT4G37280|AT2G40550|AT3G51300|AT5G58230|AT4G29830|AT5G23720|AT2G31970|AT1G07790|AT1G08560|AT3G53730|AT4G05520|AT1G80350|AT5G43990|AT3G56960|AT3G56640|AT2G13680|AT3G20670|AT3G59550|AT1G76540|AT5G62410|AT4G14150|AT1G03780|AT1G79250|AT3G28780|AT3G54670|AT3G07880|AT3G06030|AT5G49500|AT3G01700|AT4G22970|AT5G57320|AT4G30870|AT1G01510|AT1G11130|AT3G02680|AT5G23940|AT2G35630|AT3G59530|AT5G61460|AT5G67320|AT5G10400|AT5G35520|AT4G05190|AT3G12160|AT5G64610|AT1G18450|AT2G23380|AT2G41550</td></tr>
<tr><td>6996</td><td>7.68E-14</td><td>2.32E-11</td><td>44</td><td>526</td><td>502</td><td>22304</td><td>organelle organization</td><td>AT5G67270|AT3G54670|AT1G63020|AT3G06030|AT5G22880|AT3G23670|AT2G46180|AT1G04050|AT5G18620|AT1G57820|AT2G35160|AT2G34790|AT3G20475|AT5G13960|AT5G57320|AT4G38130|AT4G37280|AT2G40550|AT4G30870|AT1G01510|AT5G58230|AT3G02680|AT4G29830|AT5G23720|AT2G35630|AT2G31970|AT1G07790|AT3G53730|AT1G80350|AT5G43990|AT3G20670|AT5G61460|AT3G59550|AT5G67320|AT5G10400|AT5G35520|AT4G05190|AT5G64610|AT1G76540|AT1G18450|AT5G62410|AT2G23380|AT4G14150|AT1G03780</td></tr>
<tr><td>51726</td><td>6.17E-13</td><td>1.40E-10</td><td>20</td><td>111</td><td>502</td><td>22304</td><td>regulation of cell cycle</td><td>AT1G07270|AT5G48820|AT5G24330|AT2G17620|AT5G65420|AT4G03270|AT3G19590|AT4G22970|AT3G50070|AT2G33560|AT4G34160|AT2G20000|AT5G09790|AT1G18040|AT1G76540|AT1G70210|AT4G35620|AT1G78770|AT3G50410|AT5G58230</td></tr>
<tr><td>7049</td><td>4.30E-12</td><td>7.81E-10</td><td>22</td><td>152</td><td>502</td><td>22304</td><td>cell cycle</td><td>AT3G02680|AT2G35630|AT3G54670|AT3G23670|AT5G65420|AT3G02820|AT2G13680|AT5G61460|AT4G22970|AT3G59550|AT5G35520|AT4G05190|AT4G34160|AT3G20475|AT3G63130|AT1G76540|AT1G70210|AT2G40550|AT4G30870|AT4G14150|AT1G78770|AT1G03780</td></tr>
<tr><td>22402</td><td>3.40E-11</td><td>5.14E-09</td><td>19</td><td>122</td><td>502</td><td>22304</td><td>cell cycle process</td><td>AT3G02680|AT2G35630|AT3G54670|AT3G23670|AT5G65420|AT5G61460|AT4G22970|AT3G59550|AT5G35520|AT4G05190|AT4G34160|AT3G20475|AT3G63130|AT1G76540|AT1G70210|AT2G40550|AT4G30870|AT4G14150|AT1G03780</td></tr>
<tr><td>6325</td><td>8.80E-11</td><td>1.14E-08</td><td>21</td><td>160</td><td>502</td><td>22304</td><td>chromatin organization</td><td>AT4G29830|AT1G07790|AT3G53730|AT1G63020|AT5G43990|AT5G22880|AT1G04050|AT3G20670|AT5G18620|AT1G57820|AT2G35160|AT5G67320|AT5G10400|AT5G13960|AT5G64610|AT4G38130|AT1G76540|AT1G18450|AT4G37280|AT2G23380|AT5G58230</td></tr>
<tr><td>6259</td><td>1.20E-10</td><td>1.36E-08</td><td>29</td><td>311</td><td>502</td><td>22304</td><td>DNA metabolic process</td><td>AT1G44900|AT1G12370|AT1G08130|AT1G57820|AT2G35160|AT4G02460|AT2G16440|AT4G24790|AT1G52500|AT3G20475|AT5G13960|AT4G29170|AT1G04020|AT2G40550|AT4G30870|AT2G01120|AT1G07270|AT3G49250|AT3G02680|AT5G16690|AT1G31360|AT2G31970|AT5G46280|AT3G02820|AT5G61460|AT4G02070|AT5G63960|AT2G24490|AT2G42120</td></tr>
</table>

<table>
<tr><td>22403</td><td>1.14E-09</td><td>1.15E-07</td><td>14</td><td>75</td><td>502</td><td>22304</td><td>cell cycle phase</td><td>AT3G02680|AT2G35630|AT5G65420|AT4G22970|AT3G59550|AT5G35520|AT4G05190|AT4G34160|AT3G20475|AT3G63130|AT1G76540|AT1G70210|AT4G30870|AT1G03780</td></tr>
<tr><td>9653</td><td>7.63E-09</td><td>6.93E-07</td><td>35</td><td>515</td><td>502</td><td>22304</td><td>anatomical structure morphogenesis</td><td>AT1G44900|AT3G28780|AT5G14380|AT5G18580|AT2G34710|AT1G59640|AT3G07880|AT3G59420|AT1G70510|AT3G01700|AT4G22970|AT3G04630|AT4G20910|AT2G20000|AT5G13300|AT2G20300|AT3G51300|AT1G01510|AT5G58230|AT2G27990|AT1G11130|AT5G23940|AT3G02000|AT1G80350|AT3G56960|AT3G56640|AT2G13680|AT3G59530|AT5G60690|AT3G12160|AT1G48410|AT2G23380|AT1G79250|AT5G13290|AT4G37750</td></tr>
<tr><td>3</td><td>1.30E-08</td><td>1.07E-06</td><td>50</td><td>931</td><td>502</td><td>22304</td><td>reproduction</td><td>AT3G55620|AT1G13120|AT5G14380|AT2G34710|AT1G59640|AT3G59420|AT1G70510|AT2G34790|AT1G75920|AT4G02460|AT5G07560|AT2G20300|AT1G01370|AT3G51300|AT5G58230|AT4G13230|AT5G40480|AT3G56960|AT3G04680|AT3G56640|AT5G24330|AT2G13680|AT1G30330|AT4G28190|AT1G48410|AT2G03850|AT1G79250|AT5G19610|AT3G01700|AT4G22970|AT4G02560|AT2G35340|AT4G20910|AT2G20000|AT1G19890|AT1G06520|AT2G27990|AT1G11130|AT5G23940|AT3G02000|AT5G27740|AT3G22990|AT1G19850|AT5G08170|AT3G12160|AT1G18450|AT5G37020|AT2G23380|AT3G07060|AT4G37750</td></tr>
<tr><td>22414</td><td>4.64E-08</td><td>3.51E-06</td><td>48</td><td>911</td><td>502</td><td>22304</td><td>reproductive process</td><td>AT3G55620|AT1G13120|AT5G14380|AT2G34710|AT1G59640|AT3G59420|AT5G19610|AT1G70510|AT3G01700|AT4G22970|AT2G34790|AT4G02460|AT4G02560|AT2G35340|AT4G20910|AT2G20000|AT2G20300|AT1G01370|AT1G19890|AT3G51300|AT5G58230|AT1G06520|AT4G13230|AT2G27990|AT1G11130|AT5G23940|AT5G40480|AT3G02000|AT3G56960|AT3G04680|AT5G27740|AT3G56640|AT5G24330|AT3G22990|AT2G13680|AT1G30330|AT1G19850|AT5G08170|AT3G12160|AT4G28190|AT1G48410|AT1G18450|AT2G03850|AT5G37020|AT2G23380|AT3G07060|AT1G79250|AT4G37750</td></tr>
<tr><td>7275</td><td>5.10E-08</td><td>3.56E-06</td><td>72</td><td>1655</td><td>502</td><td>22304</td><td>multicellular organismal development</td><td>AT3G55620|AT1G44900|AT1G13120|AT5G14380|AT2G34710|AT1G59640|AT3G23670|AT3G59420|AT1G70510|AT2G34790|AT4G02460|AT3G04630|AT4G28530|AT5G07680|AT2G20300|AT1G77980|AT3G51300|AT5G58230|AT4G13230|AT5G40480|AT1G80350|AT3G56960|AT3G04680|AT3G56640|AT5G24330|AT2G13680|AT1G30330|AT3G59550|AT5G09790|AT4G28190|AT1G48410|AT2G03850|AT4G14150|AT1G79250|AT5G13290|AT3G28780|AT3G07880|AT3G01700|AT4G22970|AT4G02560|AT2G35340|AT4G20910|AT2G20000|AT5G13300|AT5G61430|AT1G02800|AT5G28640|AT4G29170|AT1G04020|AT1G78770|AT1G01510|AT1G06520|AT2G27990|AT1G61110|AT1G11130|AT4G22140|AT5G23940|AT3G02000|AT5G27740|AT3G22990|AT5G65420|AT3G59530|AT5G60690|AT1G34355|AT1G19850|AT5G08170|AT3G12160|AT1G18450|AT5G37020|AT2G23380|AT3G07060|AT4G37750</td></tr>
<tr><td>7017</td><td>6.12E-08</td><td>3.81E-06</td><td>14</td><td>101</td><td>502</td><td>22304</td><td>microtubule-based process</td><td>AT1G12430|AT5G23720|AT2G35630|AT1G80350|AT3G06030|AT1G18550|AT2G36200|AT4G05190|AT3G63130|AT5G06670|AT4G27180|AT3G20150|AT1G03780|AT1G01510</td></tr>
<tr><td>16568</td><td>6.35E-08</td><td>3.81E-06</td><td>13</td><td>86</td><td>502</td><td>22304</td><td>chromatin modification</td><td>AT4G29830|AT1G63020|AT5G43990|AT1G04050|AT5G18620|AT1G57820|AT2G35160|AT5G67320|AT5G13960|AT4G38130|AT1G76540|AT2G23380|AT5G58230</td></tr>
</table>

<table>
<tr><td>32501</td><td>6.72E-08</td><td>3.81E-06</td><td>74</td><td>1732</td><td>502</td><td>22304</td><td>multicellular organismal process</td><td>AT3G55620|AT1G44900|AT5G67270|AT1G13120|AT5G14380|AT2G34710|AT1G59640|
AT3G23670|AT3G59420|AT1G70510|AT2G34790|AT4G02460|AT3G04630|AT4G28530|
AT5G07680|AT2G20300|AT1G77980|AT3G51300|AT5G58230|AT4G13230|AT5G40480|
AT1G80350|AT3G56960|AT3G04680|AT3G56640|AT5G24330|AT2G13680|AT1G30330|
AT3G59550|AT5G09790|AT4G28190|AT1G48410|AT2G03850|AT4G14150|AT1G79250|
AT5G13290|AT3G28780|AT3G07880|AT5G19610|AT3G01700|AT4G22970|AT4G02560|
AT2G35340|AT4G20910|AT2G20000|AT5G13300|AT5G61430|AT1G02800|AT5G28640|
AT4G29170|AT1G04020|AT1G78770|AT1G01510|AT1G06520|AT2G27990|AT1G61110|
AT1G11130|AT4G22140|AT5G23940|AT3G02000|AT5G27740|AT3G22990|AT5G65420|
AT3G59530|AT5G60690|AT1G34355|AT1G19850|AT5G08170|AT3G12160|AT1G18450|
AT5G37020|AT2G23380|AT3G07060|AT4G37750</td></tr>
<tr><td>3006</td><td>1.42E-07</td><td>7.58E-06</td><td>44</td><td>829</td><td>502</td><td>22304</td><td>reproductive developmental process</td><td>AT3G55620|AT1G13120|AT5G14380|AT2G34710|AT1G59640|AT3G59420|AT1G70510|
AT3G01700|AT4G22970|AT2G34790|AT4G02460|AT4G02560|AT2G35340|AT4G20910|
AT2G20000|AT2G20300|AT3G51300|AT5G58230|AT1G06520|AT4G13230|AT2G27990|
AT1G11130|AT5G23940|AT5G40480|AT3G02000|AT3G56960|AT3G04680|AT5G27740|
AT3G56640|AT5G24330|AT3G22990|AT2G13680|AT1G30330|AT1G19850|AT5G08170|
AT3G12160|AT4G28190|AT1G48410|AT1G18450|AT2G03850|AT5G37020|AT2G23380|
AT3G07060|AT1G79250</td></tr>
<tr><td>6260</td><td>1.87E-07</td><td>9.41E-06</td><td>13</td><td>94</td><td>502</td><td>22304</td><td>DNA replication</td><td>AT1G07270|AT1G44900|AT5G16690|AT1G08130|AT5G46280|AT3G02820|AT2G16440|
AT4G24790|AT5G63960|AT2G40550|AT2G24490|AT2G01120|AT2G42120</td></tr>
<tr><td>32502</td><td>2.29E-07</td><td>1.09E-05</td><td>75</td><td>1820</td><td>502</td><td>22304</td><td>developmental process</td><td>AT3G55620|AT1G44900|AT1G13120|AT5G14380|AT5G18580|AT2G34710|AT1G59640|
AT3G23670|AT3G59420|AT1G70510|AT2G34790|AT4G02460|AT3G04630|AT4G28530|
AT5G07680|AT2G20300|AT1G77980|AT3G51300|AT5G58230|AT4G13230|AT5G40480|
AT1G80350|AT3G56960|AT3G04680|AT3G56640|AT5G24330|AT2G13680|AT1G30330|
AT2G19620|AT3G59550|AT5G09790|AT4G28190|AT1G48410|AT2G03850|AT4G14150|
AT1G79250|AT5G13290|AT3G28780|AT3G07880|AT3G01700|AT4G22970|AT4G02560|
AT2G35340|AT4G20910|AT2G20000|AT5G13300|AT5G61430|AT1G02800|AT5G28640|
AT4G29170|AT1G04020|AT1G78770|AT1G01510|AT1G06520|AT2G27990|AT1G61110|
AT1G11130|AT4G22140|AT5G23940|AT3G02000|AT5G27740|AT3G22990|AT5G65420|
AT3G59530|AT5G60690|AT1G34355|AT1G19850|AT5G08170|AT3G12160|AT3G12680|
AT1G18450|AT5G37020|AT2G23380|AT3G07060|AT4G37750</td></tr>
</table>

<table>
<tr><td>50789</td><td>6.54E-07</td><td>2.97E-05</td><td>101</td><td>2783</td><td>502</td><td>22304</td><td>regulation of biological process</td><td>AT5G22760|AT1G44900|AT5G25510|AT1G63020|AT1G31150|AT1G57820|AT2G35160|AT4G28530|AT1G25540|AT4G14920|AT4G35620|AT3G50410|AT1G07270|AT5G23720|AT5G44200|AT1G30330|AT4G22680|AT5G09790|AT1G48410|AT5G10260|AT3G46770|AT2G24490|AT1G07540|AT1G64800|AT3G19590|AT4G22970|AT3G50070|AT2G20000|AT3G13000|AT1G18040|AT2G37290|AT3G63300|AT1G78770|AT2G45460|AT1G26610|AT1G11130|AT3G49250|AT5G48820|AT2G35310|AT3G02000|AT1G60860|AT4G31610|AT2G17620|AT5G65420|AT1G26590|AT3G12160|AT4G34160|AT3G61250|AT5G08565|AT5G64610|AT3G14980|AT5G37020|AT2G34710|AT1G59640|AT1G63100|AT4G03270|AT2G13570|AT3G59420|AT5G25150|AT5G07680|AT5G59840|AT5G13960|AT4G38130|AT5G55760|AT1G77980|AT5G66940|AT4G35700|AT3G51300|AT5G58230|AT3G51030|AT3G06740|AT4G29830|AT2G35530|AT5G24330|AT3G02820|AT2G13680|AT1G76540|AT4G28190|AT1G77470|AT5G13290|AT1G35490|AT5G20240|AT4G02560|AT2G33560|AT4G20910|AT4G34590|AT5G61430|AT1G70210|AT1G04020|AT4G31360|AT2G27990|AT4G34990|AT1G61110|AT4G22140|AT3G05670|AT1G27360|AT5G60690|AT1G16070|AT2G23380|AT4G14770|AT4G37750</td></tr>
<tr><td>65007</td><td>9.09E-07</td><td>3.93E-05</td><td>113</td><td>3243</td><td>502</td><td>22304</td><td>biological regulation</td><td>AT5G22760|AT1G44900|AT5G25510|AT1G63020|AT1G31150|AT1G57820|AT2G35160|AT4G28530|AT1G25540|AT4G14920|AT4G35620|AT3G50410|AT5G59120|AT1G07270|AT5G23720|AT3G56640|AT5G44200|AT1G30330|AT4G22680|AT5G09790|AT1G48410|AT5G10260|AT3G46770|AT2G24490|AT1G07540|AT1G64800|AT3G07880|AT3G01700|AT3G19590|AT4G22970|AT3G50070|AT2G20000|AT3G13000|AT1G18040|AT2G37290|AT3G63300|AT1G78770|AT1G01510|AT2G45460|AT1G26610|AT1G11130|AT3G49250|AT5G48820|AT2G35310|AT3G02000|AT1G60860|AT4G31610|AT2G17620|AT5G65420|AT1G26590|AT3G12160|AT4G34160|AT3G61250|AT5G08565|AT5G64610|AT3G14980|AT5G37020|AT5G14380|AT5G18580|AT2G34710|AT1G59640|AT1G63100|AT4G03270|AT2G13570|AT3G59420|AT5G25150|AT5G07680|AT5G59840|AT5G13960|AT4G38130|AT5G55760|AT1G77980|AT5G66940|AT4G35700|AT3G51300|AT5G58230|AT3G51030|AT3G06740|AT4G29830|AT2G35530|AT2G31970|AT1G80350|AT3G56960|AT5G24330|AT3G02820|AT2G13680|AT5G62290|AT1G76540|AT4G28190|AT1G77470|AT1G79250|AT5G13290|AT1G35490|AT5G20240|AT4G02560|AT2G33560|AT4G20910|AT4G34590|AT5G61430|AT1G70210|AT1G04020|AT4G31360|AT2G27990|AT4G34990|AT1G61110|AT4G22140|AT3G05670|AT1G27360|AT5G60690|AT1G16070|AT2G23380|AT4G14770|AT4G37750</td></tr>
<tr><td>279</td><td>1.15E-06</td><td>4.74E-05</td><td>10</td><td>62</td><td>502</td><td>22304</td><td>M phase</td><td>AT5G35520|AT4G05190|AT3G02680|AT3G20475|AT3G63130|AT2G35630|AT4G30870|AT4G22970|AT3G59550|AT1G03780</td></tr>
<tr><td>48856</td><td>2.17E-06</td><td>8.56E-05</td><td>59</td><td>1392</td><td>502</td><td>22304</td><td>anatomical structure development</td><td>AT3G55620|AT1G44900|AT1G13120|AT5G14380|AT5G18580|AT2G34710|AT1G59640|AT3G59420|AT1G70510|AT2G34790|AT4G02460|AT3G04630|AT2G20300|AT3G51300|AT5G58230|AT4G13230|AT5G40480|AT1G80350|AT3G56960|AT3G04680|AT3G56640|AT5G24330|AT2G13680|AT1G30330|AT4G28190|AT1G48410|AT2G03850|AT1G79250|AT5G13290|AT3G28780|AT3G07880|AT3G01700|AT4G22970|AT4G02560|AT2G35340|AT4G20910|AT2G20000|AT5G13300|AT5G28640|AT1G04020|AT1G78770|AT1G01510|AT2G27990|AT1G11130|AT5G23940|AT3G02000|AT5G27740|AT3G22990|AT5G65420|AT3G59530|AT5G60690|AT1G19850|AT5G08170|AT3G12160|AT1G18450|AT5G37020|AT2G23380|AT3G07060|AT4G37750</td></tr>
</table>

48869	2.32E-06	8.79E-05	27	435	502	22304	cellular developmental process	AT3G28780IAT5G14380IAT5G18580IAT3G07880IAT3G01700IAT4G22970IAT2G34790IAT2G20000IAT3G51300IAT1G01510IAT5G58230IAT1G06520IAT2G27990IAT1G11130IAT5G23940IAT1G80350IAT3G56960IAT3G56640IAT5G65420IAT2G13680IAT3G59530IAT5G60690IAT2G19620IAT3G12160IAT3G12680IAT1G18450IAT1G79250
6974	4.44E-06	1.61E-04	15	163	502	22304	response to DNA damage stimulus	AT1G31360IAT2G31970IAT1G12370IAT1G08130IAT3G02820IAT5G61460IAT4G02070IAT4G02460IAT1G52500IAT3G20475IAT4G29170IAT1G04020IAT2G40550IAT2G24490IAT4G30870
278	5.80E-06	2.00E-04	7	32	502	22304	mitotic cell cycle	AT5G35520IAT4G34160IAT2G35630IAT1G76540IAT1G70210IAT5G65420IAT3G59550
50794	5.94E-06	2.00E-04	88	2448	502	22304	regulation of cellular process	AT5G22760IAT1G44900IAT5G25510IAT2G34710IAT1G59640IAT1G63020IAT1G63100IAT1G31150IAT4G03270IAT2G13570IAT3G59420IAT5G25150IAT1G57820IAT2G35160IAT4G28530IAT1G25540IAT5G07680IAT5G59840IAT4G14920IAT4G38130IAT5G55760IAT1G77980IAT5G66940IAT4G35700IAT3G51300IAT4G35620IAT3G50410IAT5G58230IAT3G51030IAT3G06740IAT1G07270IAT5G23720IAT2G35530IAT5G24330IAT3G02820IAT2G13680IAT1G30330IAT4G22680IAT5G09790IAT1G76540IAT5G10260IAT3G46770IAT1G07540IAT1G64800IAT1G35490IAT5G20240IAT3G19590IAT4G22970IAT3G50070IAT2G33560IAT2G20000IAT4G34590IAT3G13000IAT5G61430IAT1G18040IAT2G37290IAT3G63300IAT1G70210IAT1G78770IAT4G31360IAT2G45460IAT1G26610IAT2G27990IAT4G34990IAT1G61110IAT1G11130IAT4G22140IAT5G48820IAT2G35310IAT3G02000IAT1G60860IAT4G31610IAT3G05670IAT2G17620IAT5G65420IAT1G26590IAT1G27360IAT5G60690IAT3G12160IAT4G34160IAT3G61250IAT1G16070IAT5G08565IAT5G64610IAT3G14980IAT5G37020IAT4G14770IAT4G37750
9555	7.25E-06	2.35E-04	14	149	502	22304	pollen development	AT3G28780IAT3G23670IAT2G13680IAT3G59530IAT1G34355IAT3G59550IAT4G02460IAT5G09790IAT1G77980IAT1G18450IAT4G29170IAT4G14150IAT5G58230IAT1G06520
6281	1.06E-05	3.32E-04	14	154	502	22304	DNA repair	AT1G31360IAT2G31970IAT1G12370IAT1G08130IAT5G61460IAT4G02070IAT4G02460IAT1G52500IAT3G20475IAT4G29170IAT1G04020IAT2G40550IAT2G24490IAT4G30870
7010	1.17E-05	3.54E-04	11	97	502	22304	cytoskeleton organization	AT4G05190IAT5G23720IAT5G67270IAT2G35630IAT5G57320IAT1G80350IAT3G06030IAT3G23670IAT4G14150IAT1G03780IAT1G01510
60255	1.40E-05	3.99E-04	65	1685	502	22304	regulation of macromolecule metabolic process	AT5G22760IAT2G34710IAT1G59640IAT1G63020IAT1G63100IAT1G31150IAT2G13570IAT5G25150IAT1G57820IAT2G35160IAT4G28530IAT1G25540IAT5G07680IAT5G13960IAT4G14920IAT4G38130IAT5G55760IAT1G77980IAT5G66940IAT4G35700IAT3G50410IAT5G58230IAT3G06740IAT5G23720IAT2G35530IAT5G44200IAT5G24330IAT3G02820IAT1G30330IAT4G22680IAT5G09790IAT1G48410IAT3G46770IAT2G24490IAT1G07540IAT1G64800IAT1G35490IAT5G20240IAT4G20910IAT4G34590IAT3G13000IAT5G61430IAT1G78770IAT2G45460IAT1G26610IAT4G34990IAT1G61110IAT4G22140IAT3G49250IAT2G35310IAT3G02000IAT4G31610IAT3G05670IAT1G26590IAT1G27360IAT5G60690IAT3G61250IAT1G16070IAT5G08565IAT5G64610IAT3G14980IAT5G37020IAT2G23380IAT4G14770IAT4G37750
30154	1.41E-05	3.99E-04	20	296	502	22304	cell differentiation	AT5G23940IAT5G14380IAT3G07880IAT1G80350IAT3G56960IAT3G56640IAT2G13680IAT5G60690IAT3G01700IAT2G19620IAT2G34790IAT3G12160IAT2G20000IAT3G12680IAT1G18450IAT3G51300IAT1G79250IAT5G58230IAT1G06520IAT2G27990

<table>
<tr><th></th><th></th><th></th><th></th><th></th><th></th><th></th><th></th><th></th></tr>
<tr><td>51128</td><td>1.93E-05</td><td>5.24E-04</td><td>7</td><td>38</td><td>502</td><td>22304</td><td>regulation of cellular component organization</td><td>AT3G12160|AT2G20000|AT2G13680|AT3G51300|AT3G19590|AT4G22970|AT2G33560</td></tr>
<tr><td>6310</td><td>1.96E-05</td><td>5.24E-04</td><td>8</td><td>52</td><td>502</td><td>22304</td><td>DNA recombination</td><td>AT3G02680|AT3G20475|AT1G31360|AT2G31970|AT1G08130|AT4G30870|AT5G61460|AT4G02460</td></tr>
<tr><td>10468</td><td>2.27E-05</td><td>5.89E-04</td><td>63</td><td>1642</td><td>502</td><td>22304</td><td>regulation of gene expression</td><td>AT5G22760|AT2G34710|AT1G59640|AT1G63020|AT1G63100|AT1G31150|AT2G13570|AT5G25150|AT1G57820|AT2G35160|AT4G28530|AT1G25540|AT5G07680|AT5G13960|AT4G14920|AT4G38130|AT5G55760|AT1G77980|AT5G66940|AT4G35700|AT3G50410|AT5G58230|AT3G06740|AT5G23720|AT2G35530|AT5G44200|AT5G24330|AT1G30330|AT4G22680|AT5G09790|AT1G48410|AT3G46770|AT2G24490|AT1G07540|AT1G64800|AT1G35490|AT5G20240|AT4G20910|AT4G34590|AT3G13000|AT5G61430|AT2G45460|AT1G26610|AT4G34990|AT1G61110|AT4G22140|AT3G49250|AT2G35310|AT3G02000|AT4G31610|AT3G05670|AT1G26590|AT1G27360|AT5G60690|AT3G61250|AT1G16070|AT5G08565|AT5G64610|AT3G14980|AT5G37020|AT2G23380|AT4G14770|AT4G37750</td></tr>
<tr><td>22607</td><td>2.46E-05</td><td>6.20E-04</td><td>18</td><td>258</td><td>502</td><td>22304</td><td>cellular component assembly</td><td>AT3G28780|AT2G35630|AT1G07790|AT3G53730|AT5G22880|AT3G23670|AT5G49500|AT2G13680|AT3G59530|AT3G20670|AT1G57820|AT5G10400|AT4G05190|AT3G20475|AT4G30870|AT4G14150|AT1G03780|AT5G58230</td></tr>
<tr><td>71103</td><td>2.59E-05</td><td>6.33E-04</td><td>10</td><td>87</td><td>502</td><td>22304</td><td>DNA conformation change</td><td>AT1G44900|AT1G07790|AT3G53730|AT5G22880|AT5G46280|AT3G20670|AT1G57820|AT2G16440|AT5G58230|AT5G10400</td></tr>
<tr><td>19222</td><td>2.65E-05</td><td>6.33E-04</td><td>68</td><td>1825</td><td>502</td><td>22304</td><td>regulation of metabolic process</td><td>AT5G22760|AT2G34710|AT1G59640|AT1G63020|AT1G63100|AT1G31150|AT2G13570|AT5G25150|AT1G57820|AT2G35160|AT4G28530|AT1G25540|AT5G07680|AT5G13960|AT4G14920|AT4G38130|AT5G55760|AT1G77980|AT5G66940|AT4G35700|AT3G50410|AT5G58230|AT3G06740|AT5G23720|AT2G35530|AT5G44200|AT5G24330|AT3G02820|AT1G30330|AT4G22680|AT5G09790|AT1G48410|AT3G46770|AT2G24490|AT1G07540|AT1G64800|AT1G35490|AT5G20240|AT4G20910|AT4G34590|AT3G13000|AT5G61430|AT2G37290|AT1G78770|AT2G45460|AT1G26610|AT4G34990|AT1G61110|AT4G22140|AT3G49250|AT5G48820|AT2G35310|AT3G02000|AT1G60860|AT4G31610|AT3G05670|AT1G26590|AT1G27360|AT5G60690|AT3G61250|AT1G16070|AT5G08565|AT5G64610|AT3G14980|AT5G37020|AT2G23380|AT4G14770|AT4G37750</td></tr>
<tr><td>226</td><td>2.75E-05</td><td>6.40E-04</td><td>7</td><td>40</td><td>502</td><td>22304</td><td>microtubule cytoskeleton organization</td><td>AT4G05190|AT5G23720|AT2G35630|AT1G80350|AT3G06030|AT1G03780|AT1G01510</td></tr>
<tr><td>48608</td><td>2.85E-05</td><td>6.47E-04</td><td>35</td><td>735</td><td>502</td><td>22304</td><td>reproductive structure development</td><td>AT3G55620|AT1G13120|AT2G34710|AT1G59640|AT3G59420|AT1G70510|AT4G22970|AT2G34790|AT4G02460|AT4G02560|AT2G35340|AT4G20910|AT2G20000|AT2G20300|AT5G58230|AT4G13230|AT2G27990|AT1G11130|AT5G23940|AT5G40480|AT3G02000|AT3G04680|AT5G27740|AT5G24330|AT3G22990|AT1G30330|AT1G19850|AT5G08170|AT4G28190|AT1G48410|AT1G18450|AT2G03850|AT5G37020|AT2G23380|AT3G07060</td></tr>
<tr><td>6333</td><td>3.73E-05</td><td>8.26E-04</td><td>9</td><td>73</td><td>502</td><td>22304</td><td>chromatin assembly or disassembly</td><td>AT1G07790|AT5G64610|AT3G53730|AT5G22880|AT4G37280|AT3G20670|AT1G57820|AT5G58230|AT5G10400</td></tr>
<tr><td>80</td><td>4.46E-05</td><td>9.41E-04</td><td>3</td><td>4</td><td>502</td><td>22304</td><td>G1 phase of mitotic cell cycle</td><td>AT4G34160|AT1G70210|AT5G65420</td></tr>
<tr><td>51318</td><td>4.46E-05</td><td>9.41E-04</td><td>3</td><td>4</td><td>502</td><td>22304</td><td>G1 phase</td><td>AT4G34160|AT1G70210|AT5G65420</td></tr>
</table>

<table>
<tr><td>9887</td><td>5.37E-05</td><td>1.11E-03</td><td>14</td><td>178</td><td>502</td><td>22304</td><td>organ morphogenesis</td><td>AT5G23940|AT3G02000|AT1G59640|AT1G80350|AT1G70510|AT4G20910|AT5G13300|AT2G20300|AT1G48410|AT2G23380|AT1G01510|AT5G58230|AT2G27990|AT4G37750</td></tr>
<tr><td>48507</td><td>5.57E-05</td><td>1.12E-03</td><td>10</td><td>95</td><td>502</td><td>22304</td><td>meristem development</td><td>AT1G11130|AT1G44900|AT2G20000|AT2G34710|AT2G20300|AT4G28190|AT5G60690|AT1G19850|AT5G13290|AT2G27990</td></tr>
<tr><td>19219</td><td>6.51E-05</td><td>1.28E-03</td><td>58</td><td>1527</td><td>502</td><td>22304</td><td>regulation of nucleobase, nucleoside, nucleotide and nucleic acid metabolic process</td><td>AT5G22760|AT2G34710|AT1G59640|AT1G63020|AT1G63100|AT1G31150|AT2G13570|AT5G25150|AT1G57820|AT2G35160|AT4G28530|AT1G25540|AT5G07680|AT4G14920|AT4G38130|AT5G55760|AT1G77980|AT5G66940|AT4G35700|AT3G50410|AT3G06740|AT2G35530|AT5G24330|AT3G02820|AT1G30330|AT4G22680|AT5G09790|AT3G46770|AT1G07540|AT1G64800|AT1G35490|AT5G20240|AT4G34590|AT3G13000|AT5G61430|AT2G37290|AT1G78770|AT2G45460|AT1G26610|AT4G34990|AT1G61110|AT4G22140|AT2G35310|AT3G02000|AT1G60860|AT4G31610|AT3G05670|AT1G26590|AT1G27360|AT5G60690|AT3G61250|AT1G16070|AT5G08565|AT5G64610|AT3G14980|AT5G37020|AT4G14770|AT4G37750</td></tr>
<tr><td>90304</td><td>7.51E-05</td><td>1.45E-03</td><td>38</td><td>866</td><td>502</td><td>22304</td><td>nucleic acid metabolic process</td><td>AT1G44900|AT5G51940|AT1G12370|AT1G63020|AT1G08130|AT5G64200|AT1G57820|AT2G35160|AT4G02460|AT2G16440|AT3G19090|AT4G24790|AT1G52500|AT3G20475|AT4G20910|AT5G13960|AT4G29170|AT1G04020|AT2G40550|AT4G30870|AT2G01120|AT1G80070|AT1G07270|AT3G49250|AT3G02680|AT5G16690|AT1G31360|AT2G31970|AT3G04680|AT5G44200|AT5G46280|AT3G02820|AT5G61460|AT4G02070|AT5G63960|AT2G24490|AT2G41550|AT2G42120</td></tr>
<tr><td>16569</td><td>8.14E-05</td><td>1.51E-03</td><td>8</td><td>63</td><td>502</td><td>22304</td><td>covalent chromatin modification</td><td>AT4G29830|AT5G13960|AT4G38130|AT1G63020|AT1G76540|AT2G23380|AT2G35160|AT5G67320</td></tr>
<tr><td>19953</td><td>8.14E-05</td><td>1.51E-03</td><td>8</td><td>63</td><td>502</td><td>22304</td><td>sexual reproduction</td><td>AT5G07560|AT2G20000|AT1G01370|AT1G18450|AT1G19890|AT1G75920|AT1G06520|AT4G37750</td></tr>
<tr><td>51171</td><td>8.95E-05</td><td>1.60E-03</td><td>58</td><td>1545</td><td>502</td><td>22304</td><td>regulation of nitrogen compound metabolic process</td><td>AT5G22760|AT2G34710|AT1G59640|AT1G63020|AT1G63100|AT1G31150|AT2G13570|AT5G25150|AT1G57820|AT2G35160|AT4G28530|AT1G25540|AT5G07680|AT4G14920|AT4G38130|AT5G55760|AT1G77980|AT5G66940|AT4G35700|AT3G50410|AT3G06740|AT2G35530|AT5G24330|AT3G02820|AT1G30330|AT4G22680|AT5G09790|AT3G46770|AT1G07540|AT1G64800|AT1G35490|AT5G20240|AT4G34590|AT3G13000|AT5G61430|AT2G37290|AT1G78770|AT2G45460|AT1G26610|AT4G34990|AT1G61110|AT4G22140|AT2G35310|AT3G02000|AT1G60860|AT4G31610|AT3G05670|AT1G26590|AT1G27360|AT5G60690|AT3G61250|AT1G16070|AT5G08565|AT5G64610|AT3G14980|AT5G37020|AT4G14770|AT4G37750</td></tr>
<tr><td>32989</td><td>9.17E-05</td><td>1.60E-03</td><td>17</td><td>260</td><td>502</td><td>22304</td><td>cellular component morphogenesis</td><td>AT1G11130|AT3G28780|AT5G23940|AT5G14380|AT5G18580|AT3G07880|AT1G80350|AT3G56960|AT3G56640|AT2G13680|AT3G59530|AT3G01700|AT4G22970|AT3G12160|AT3G51300|AT1G79250|AT1G01510</td></tr>
<tr><td>9888</td><td>9.17E-05</td><td>1.60E-03</td><td>17</td><td>260</td><td>502</td><td>22304</td><td>tissue development</td><td>AT1G11130|AT1G44900|AT5G23940|AT2G34710|AT3G07880|AT1G80350|AT5G60690|AT4G22970|AT1G19850|AT2G20000|AT5G13300|AT2G20300|AT4G28190|AT1G78770|AT5G13290|AT5G58230|AT2G27990</td></tr>
<tr><td>48229</td><td>9.83E-05</td><td>1.64E-03</td><td>15</td><td>212</td><td>502</td><td>22304</td><td>gametophyte development</td><td>AT3G28780|AT3G23670|AT2G13680|AT3G59530|AT1G34355|AT3G59550|AT2G34790|AT4G02460|AT5G09790|AT1G77980|AT1G18450|AT4G29170|AT4G14150|AT5G58230|AT1G06520</td></tr>
</table>

<table>
<tr><td>6464</td><td>9.90E-05</td><td>1.64E-03</td><td>49</td><td>1241</td><td>502</td><td>22304</td><td>protein modification process</td><td>AT1G64630|AT4G21060|AT2G28590|AT5G43020|AT1G55200|AT1G57820|AT2G35160|AT5G13960|AT4G38130|AT5G55760|AT1G75640|AT1G27120|AT5G11020|AT5G51560|AT2G44830|AT3G51740|AT4G35500|AT2G40790|AT5G47750|AT4G29830|AT4G26840|AT5G15460|AT5G24970|AT1G09450|AT1G76540|AT4G39110|AT5G56580|AT1G61860|AT1G62950|AT3G24660|AT2G36350|AT3G04810|AT3G53380|AT5G28290|AT5G08110|AT3G57830|AT3G02000|AT3G56100|AT2G32765|AT5G23580|AT5G67200|AT5G67320|AT1G11730|AT4G36180|AT4G17483|AT2G23380|AT3G10540|AT5G51350|AT2G24230</td></tr>
<tr><td>22603</td><td>9.93E-05</td><td>1.64E-03</td><td>6</td><td>34</td><td>502</td><td>22304</td><td>regulation of anatomical structure morphogenesis</td><td>AT1G11130|AT3G12160|AT2G20000|AT1G76540|AT1G04020|AT2G13680</td></tr>
<tr><td>9932</td><td>1.25E-04</td><td>2.02E-03</td><td>9</td><td>85</td><td>502</td><td>22304</td><td>cell tip growth</td><td>AT3G12160|AT5G14380|AT3G07880|AT3G56960|AT3G56640|AT2G13680|AT3G51300|AT3G01700|AT1G79250</td></tr>
<tr><td>9860</td><td>1.27E-04</td><td>2.02E-03</td><td>8</td><td>67</td><td>502</td><td>22304</td><td>pollen tube growth</td><td>AT3G12160|AT5G14380|AT3G56960|AT3G56640|AT2G13680|AT3G51300|AT3G01700|AT1G79250</td></tr>
<tr><td>48638</td><td>1.38E-04</td><td>2.17E-03</td><td>6</td><td>36</td><td>502</td><td>22304</td><td>regulation of developmental growth</td><td>AT1G44900|AT3G12160|AT2G20000|AT4G28190|AT2G13680|AT5G13290</td></tr>
<tr><td>10556</td><td>1.47E-04</td><td>2.27E-03</td><td>56</td><td>1504</td><td>502</td><td>22304</td><td>regulation of macromolecule biosynthetic process</td><td>AT5G22760|AT2G34710|AT1G59640|AT1G63020|AT1G63100|AT1G31150|AT2G13570|AT5G25150|AT1G57820|AT2G35160|AT4G28530|AT1G25540|AT5G07680|AT4G14920|AT4G38130|AT5G55760|AT1G77980|AT5G66940|AT4G35700|AT3G50410|AT3G06740|AT2G35530|AT5G24330|AT3G02820|AT1G30330|AT4G22680|AT5G09790|AT3G46770|AT1G07540|AT1G64800|AT1G35490|AT5G20240|AT4G34590|AT3G13000|AT5G61430|AT1G78770|AT2G45460|AT1G26610|AT4G34990|AT1G61110|AT4G22140|AT2G35310|AT3G02000|AT4G31610|AT3G05670|AT1G26590|AT1G27360|AT5G60690|AT3G61250|AT1G16070|AT5G08565|AT5G64610|AT3G14980|AT5G37020|AT4G14770|AT4G37750</td></tr>
<tr><td>51325</td><td>1.54E-04</td><td>2.30E-03</td><td>4</td><td>13</td><td>502</td><td>22304</td><td>interphase</td><td>AT4G34160|AT1G76540|AT1G70210|AT5G65420</td></tr>
<tr><td>51329</td><td>1.54E-04</td><td>2.30E-03</td><td>4</td><td>13</td><td>502</td><td>22304</td><td>interphase of mitotic cell cycle</td><td>AT4G34160|AT1G76540|AT1G70210|AT5G65420</td></tr>
<tr><td>50793</td><td>1.65E-04</td><td>2.42E-03</td><td>17</td><td>273</td><td>502</td><td>22304</td><td>regulation of developmental process</td><td>AT1G11130|AT4G22140|AT1G44900|AT4G29830|AT1G27360|AT2G13680|AT4G02560|AT1G25540|AT3G12160|AT4G20910|AT2G20000|AT1G76540|AT4G28190|AT1G04020|AT5G13290|AT5G58230|AT2G27990</td></tr>
<tr><td>7346</td><td>2.12E-04</td><td>3.05E-03</td><td>4</td><td>14</td><td>502</td><td>22304</td><td>regulation of mitotic cell cycle</td><td>AT2G20000|AT1G76540|AT3G19590|AT2G33560</td></tr>
<tr><td>48513</td><td>2.15E-04</td><td>3.05E-03</td><td>32</td><td>719</td><td>502</td><td>22304</td><td>organ development</td><td>AT1G44900|AT2G34710|AT1G59640|AT3G07880|AT3G59420|AT1G70510|AT4G22970|AT3G04630|AT4G20910|AT2G20000|AT5G13300|AT2G20300|AT5G28640|AT1G04020|AT1G78770|AT1G01510|AT5G58230|AT2G27990|AT1G11130|AT5G23940|AT3G02000|AT1G80350|AT3G04680|AT5G24330|AT3G22990|AT5G60690|AT1G19850|AT4G28190|AT1G48410|AT2G23380|AT5G13290|AT4G37750</td></tr>
<tr><td>48731</td><td>2.21E-04</td><td>3.08E-03</td><td>32</td><td>720</td><td>502</td><td>22304</td><td>system development</td><td>AT1G44900|AT2G34710|AT1G59640|AT3G07880|AT3G59420|AT1G70510|AT4G22970|AT3G04630|AT4G20910|AT2G20000|AT5G13300|AT2G20300|AT5G28640|AT1G04020|AT1G78770|AT1G01510|AT5G58230|AT2G27990|AT1G11130|AT5G23940|AT3G02000|AT1G80350|AT3G04680|AT5G24330|AT3G22990|AT5G60690|AT1G19850|AT4G28190|AT1G48410|AT2G23380|AT5G13290|AT4G37750</td></tr>
<tr><td>51239</td><td>2.30E-04</td><td>3.16E-03</td><td>15</td><td>229</td><td>502</td><td>22304</td><td>regulation of multicellular organismal process</td><td>AT1G11130|AT4G22140|AT1G44900|AT4G29830|AT1G27360|AT2G13680|AT4G02560|AT1G25540|AT3G12160|AT4G20910|AT1G76540|AT4G28190|AT1G04020|AT5G13290|AT5G58230</td></tr>
</table>

<table>
<tr><td>9791</td><td>2.38E-04</td><td>3.20E-03</td><td>37</td><td>884</td><td>502</td><td>22304</td><td>post-embryonic development</td><td>AT3G55620|AT1G13120|AT2G34710|AT1G59640|AT3G59420|AT1G70510|AT4G22970|AT2G34790|AT4G02460|AT4G02560|AT2G35340|AT4G20910|AT2G20000|AT2G20300|AT5G58230|AT4G13230|AT2G27990|AT1G11130|AT4G22140|AT5G23940|AT5G40480|AT3G02000|AT3G04680|AT5G27740|AT5G24330|AT3G22990|AT5G65420|AT1G30330|AT1G19850|AT5G08170|AT4G28190|AT1G48410|AT1G18450|AT2G03850|AT5G37020|AT2G23380|AT3G07060</td></tr>
<tr><td>80090</td><td>2.40E-04</td><td>3.20E-03</td><td>58</td><td>1604</td><td>502</td><td>22304</td><td>regulation of primary metabolic process</td><td>AT5G22760|AT2G34710|AT1G59640|AT1G63020|AT1G63100|AT1G31150|AT2G13570|AT5G25150|AT1G57820|AT2G35160|AT4G28530|AT1G25540|AT5G07680|AT4G14920|AT4G38130|AT5G55760|AT1G77980|AT5G66940|AT4G35700|AT3G50410|AT3G06740|AT2G35530|AT5G24330|AT3G02820|AT1G30330|AT4G22680|AT5G09790|AT3G46770|AT1G07540|AT1G64800|AT1G35490|AT5G20240|AT4G34590|AT3G13000|AT5G61430|AT2G37290|AT1G78770|AT2G45460|AT1G26610|AT4G34990|AT1G61110|AT4G22140|AT2G35310|AT3G02000|AT1G60860|AT4G31610|AT3G05670|AT1G26590|AT1G27360|AT5G60690|AT3G61250|AT1G16070|AT5G08565|AT5G64610|AT3G14980|AT5G37020|AT4G14770|AT4G37750</td></tr>
<tr><td>10564</td><td>2.52E-04</td><td>3.31E-03</td><td>5</td><td>26</td><td>502</td><td>22304</td><td>regulation of cell cycle process</td><td>AT1G76540|AT3G19590|AT1G78770|AT4G22970|AT2G33560</td></tr>
<tr><td>45449</td><td>2.62E-04</td><td>3.38E-03</td><td>54</td><td>1468</td><td>502</td><td>22304</td><td>regulation of transcription</td><td>AT5G22760|AT2G34710|AT1G59640|AT1G63020|AT1G63100|AT1G31150|AT2G13570|AT5G25150|AT1G57820|AT2G35160|AT4G28530|AT1G25540|AT5G07680|AT4G14920|AT4G38130|AT5G55760|AT1G77980|AT5G66940|AT4G35700|AT3G50410|AT3G06740|AT2G35530|AT5G24330|AT1G30330|AT4G22680|AT5G09790|AT3G46770|AT1G07540|AT1G64800|AT1G35490|AT5G20240|AT4G34590|AT3G13000|AT5G61430|AT2G45460|AT1G26610|AT4G34990|AT1G61110|AT4G22140|AT2G35310|AT3G02000|AT4G31610|AT3G05670|AT1G26590|AT1G27360|AT5G60690|AT3G61250|AT1G16070|AT5G08565|AT5G64610|AT3G14980|AT5G37020|AT4G14770|AT4G37750</td></tr>
<tr><td>31326</td><td>2.68E-04</td><td>3.38E-03</td><td>56</td><td>1540</td><td>502</td><td>22304</td><td>regulation of cellular biosynthetic process</td><td>AT5G22760|AT2G34710|AT1G59640|AT1G63020|AT1G63100|AT1G31150|AT2G13570|AT5G25150|AT1G57820|AT2G35160|AT4G28530|AT1G25540|AT5G07680|AT4G14920|AT4G38130|AT5G55760|AT1G77980|AT5G66940|AT4G35700|AT3G50410|AT3G06740|AT2G35530|AT5G24330|AT3G02820|AT1G30330|AT4G22680|AT5G09790|AT3G46770|AT1G07540|AT1G64800|AT1G35490|AT5G20240|AT4G34590|AT3G13000|AT5G61430|AT1G78770|AT2G45460|AT1G26610|AT4G34990|AT1G61110|AT4G22140|AT2G35310|AT3G02000|AT4G31610|AT3G05670|AT1G26590|AT1G27360|AT5G60690|AT3G61250|AT1G16070|AT5G08565|AT5G64610|AT3G14980|AT5G37020|AT4G14770|AT4G37750</td></tr>
<tr><td>9889</td><td>2.68E-04</td><td>3.38E-03</td><td>56</td><td>1540</td><td>502</td><td>22304</td><td>regulation of biosynthetic process</td><td>AT5G22760|AT2G34710|AT1G59640|AT1G63020|AT1G63100|AT1G31150|AT2G13570|AT5G25150|AT1G57820|AT2G35160|AT4G28530|AT1G25540|AT5G07680|AT4G14920|AT4G38130|AT5G55760|AT1G77980|AT5G66940|AT4G35700|AT3G50410|AT3G06740|AT2G35530|AT5G24330|AT3G02820|AT1G30330|AT4G22680|AT5G09790|AT3G46770|AT1G07540|AT1G64800|AT1G35490|AT5G20240|AT4G34590|AT3G13000|AT5G61430|AT1G78770|AT2G45460|AT1G26610|AT4G34990|AT1G61110|AT4G22140|AT2G35310|AT3G02000|AT4G31610|AT3G05670|AT1G26590|AT1G27360|AT5G60690|AT3G61250|AT1G16070|AT5G08565|AT5G64610|AT3G14980|AT5G37020|AT4G14770|AT4G37750</td></tr>
</table>

31497	2.80E-04	3.49E-03	7	57	502	22304	chromatin assembly	AT1G07790IAT3G53730IAT5G22880IAT3G20670IAT1G57820IAT5G58230IAT5G10400
30865	2.84E-04	3.49E-03	4	15	502	22304	cortical cytoskeleton organization	AT5G23720IAT5G67270IAT1G80350IAT3G06030
9793	3.24E-04	3.93E-03	20	372	502	22304	embryonic development ending in seed dormancy	AT1G11130IAT3G55620IAT5G23940IAT1G13120IAT5G40480IAT2G34710IAT5G27740IAT3G59420IAT4G22970IAT1G19850IAT2G34790IAT5G08170IAT2G35340IAT2G20000IAT2G20300IAT1G48410IAT2G03850IAT3G07060IAT5G58230IAT4G13230
904	3.39E-04	4.06E-03	10	118	502	22304	cell morphogenesis involved in differentiation	AT3G12160IAT5G23940IAT5G14380IAT1G80350IAT3G56960IAT3G56640IAT2G13680IAT3G51300IAT3G01700IAT1G79250
902	3.47E-04	4.08E-03	15	238	502	22304	cell morphogenesis	AT1G11130IAT5G23940IAT5G14380IAT5G18580IAT3G07880IAT1G80350IAT3G56960IAT3G56640IAT2G13680IAT3G01700IAT4G22970IAT3G12160IAT3G51300IAT1G79250IAT1G01510
31323	3.50E-04	4.08E-03	59	1664	502	22304	regulation of cellular metabolic process	AT5G22760IAT2G34710IAT1G59640IAT1G63020IAT1G63100IAT1G31150IAT2G13570IAT5G25150IAT1G57820IAT2G35160IAT4G28530IAT1G25540IAT5G07680IAT4G14920IAT4G38130IAT5G55760IAT1G77980IAT5G66940IAT4G35700IAT3G50410IAT3G06740IAT2G35530IAT5G24330IAT3G02820IAT1G30330IAT4G22680IAT5G09790IAT3G46770IAT1G07540IAT1G64800IAT1G35490IAT5G20240IAT4G34590IAT3G13000IAT5G61430IAT2G37290IAT1G78770IAT2G45460IAT1G26610IAT4G34990IAT1G61110IAT4G22140IAT5G48820IAT2G35310IAT3G02000IAT1G60860IAT4G31610IAT3G05670IAT1G26590IAT1G27360IAT5G60690IAT3G61250IAT1G16070IAT5G08565IAT5G64610IAT3G14980IAT5G37020IAT4G14770IAT4G37750
6323	3.87E-04	4.39E-03	7	60	502	22304	DNA packaging	AT1G07790IAT3G53730IAT5G22880IAT3G20670IAT1G57820IAT5G58230IAT5G10400
16570	3.87E-04	4.39E-03	7	60	502	22304	histone modification	AT4G29830IAT5G13960IAT4G38130IAT1G76540IAT2G23380IAT2G35160IAT5G67320
7167	4.28E-04	4.74E-03	9	100	502	22304	enzyme linked receptor protein signaling pathway	AT3G57830IAT5G43020IAT1G62950IAT3G56100IAT4G36180IAT3G24660IAT1G75640IAT5G51350IAT3G51740
7169	4.28E-04	4.74E-03	9	100	502	22304	transmembrane receptor protein tyrosine kinase signaling pathway	AT3G57830IAT5G43020IAT1G62950IAT3G56100IAT4G36180IAT3G24660IAT1G75640IAT5G51350IAT3G51740
7018	4.88E-04	5.34E-03	6	45	502	22304	microtubule-based movement	AT1G12430IAT5G06670IAT4G27180IAT3G20150IAT1G18550IAT2G36200
48646	5.33E-04	5.76E-03	9	103	502	22304	anatomical structure formation involved in morphogenesis	AT3G28780IAT4G20910IAT3G02000IAT2G20300IAT3G59420IAT2G13680IAT3G59530IAT1G70510IAT2G27990
43687	5.45E-04	5.82E-03	42	1093	502	22304	post-translational protein modification	AT1G64630IAT1G61860IAT2G28590IAT5G43020IAT1G62950IAT3G24660IAT2G36350IAT1G55200IAT3G04810IAT1G57820IAT2G35160IAT3G53380IAT5G28290IAT5G13960IAT4G38130IAT5G55760IAT1G75640IAT5G11020IAT5G51560IAT2G44830IAT3G51740IAT4G35500IAT2G40790IAT3G57830IAT5G47750IAT4G29830IAT3G02000IAT4G26840IAT5G24970IAT3G56100IAT1G09450IAT5G23580IAT5G67200IAT5G67320IAT1G76540IAT4G36180IAT2G23380IAT3G10540IAT5G51350IAT4G39110IAT5G56580IAT2G24230

43412	6.06E-04	6.40E-03	50	1378	502	22304	macromolecule modification	AT1G64630lAT4G21060lAT2G28590lAT5G43020lAT1G55200lAT1G57820lAT2G35160lAT5G13960lAT4G38130lAT5G55760lAT1G75640lAT1G27120lAT5G11020lAT5G51560lAT2G44830lAT3G51740lAT4G35500lAT2G40790lAT5G47750lAT4G29830lAT4G26840lAT5G15460lAT5G24970lAT1G09450lAT1G76540lAT4G39110lAT5G56580lAT1G61860lAT1G62950lAT3G24660lAT2G36350lAT3G04810lAT3G53380lAT5G28290lAT5G08110lAT3G57830lAT3G49250lAT3G02000lAT3G56100lAT2G32765lAT5G23580lAT5G67200lAT5G67320lAT1G11730lAT4G36180lAT4G17483lAT2G23380lAT3G10540lAT5G51350lAT2G24230
48610	6.48E-04	6.76E-03	10	128	502	22304	reproductive cellular process	AT3G12160lAT5G14380lAT1G18450lAT3G56960lAT3G56640lAT2G13680lAT3G51300lAT3G01700lAT1G79250lAT1G06520
44085	6.84E-04	7.06E-03	22	454	502	22304	cellular component biogenesis	AT3G28780lAT2G35630lAT1G07790lAT3G53730lAT1G80350lAT5G22880lAT3G23670lAT5G49500lAT2G13680lAT3G59530lAT3G20670lAT1G57820lAT5G15630lAT5G10400lAT4G05190lAT3G20475lAT1G74420lAT2G03210lAT4G30870lAT4G14150lAT1G03780lAT5G58230
60560	7.73E-04	7.79E-03	12	179	502	22304	developmental growth involved in morphogenesis	AT3G12160lAT5G14380lAT5G18580lAT3G07880lAT3G56960lAT3G56640lAT2G13680lAT3G51300lAT3G01700lAT4G22970lAT1G79250lAT1G01510
9826	7.73E-04	7.79E-03	12	179	502	22304	unidimensional cell growth	AT3G12160lAT5G14380lAT5G18580lAT3G07880lAT3G56960lAT3G56640lAT2G13680lAT3G51300lAT3G01700lAT4G22970lAT1G79250lAT1G01510
9790	7.91E-04	7.89E-03	21	429	502	22304	embryonic development	AT1G11130lAT3G55620lAT1G44900lAT5G23940lAT1G13120lAT5G40480lAT2G34710lAT5G27740lAT3G59420lAT4G22970lAT1G19850lAT2G34790lAT5G08170lAT2G35340lAT2G20000lAT2G20300lAT1G48410lAT2G03850lAT3G07060lAT5G58230lAT4G13230
48588	8.93E-04	8.81E-03	8	89	502	22304	developmental cell growth	AT3G12160lAT5G14380lAT3G56960lAT3G56640lAT2G13680lAT3G51300lAT3G01700lAT1G79250
48508	9.22E-04	8.87E-03	4	20	502	22304	embryonic meristem development	AT1G11130lAT2G20000lAT2G34710lAT2G20300
55046	9.22E-04	8.87E-03	4	20	502	22304	microgametogenesis	AT1G18450lAT3G23670lAT4G14150lAT1G06520
40029	9.29E-04	8.87E-03	11	158	502	22304	regulation of gene expression, epigenetic	AT3G49250lAT4G20910lAT5G13960lAT5G55760lAT1G63020lAT1G48410lAT5G44200lAT2G23380lAT1G57820lAT2G35160lAT5G58230
10605	9.37E-04	8.87E-03	12	183	502	22304	negative regulation of macromolecule metabolic process	AT3G49250lAT4G20910lAT3G02000lAT4G34590lAT4G38130lAT5G55760lAT1G63020lAT1G48410lAT5G44200lAT3G02820lAT2G24490lAT2G35160
48316	1.03E-03	9.61E-03	21	438	502	22304	seed development	AT1G11130lAT3G55620lAT5G23940lAT1G13120lAT5G40480lAT2G34710lAT5G27740lAT3G59420lAT4G22970lAT1G19850lAT2G34790lAT4G02460lAT5G08170lAT2G35340lAT2G20000lAT2G20300lAT1G48410lAT2G03850lAT3G07060lAT5G58230lAT4G13230
35295	1.11E-03	1.02E-02	8	92	502	22304	tube development	AT3G12160lAT5G14380lAT3G56960lAT3G56640lAT2G13680lAT3G51300lAT3G01700lAT1G79250
48868	1.11E-03	1.02E-02	8	92	502	22304	pollen tube development	AT3G12160lAT5G14380lAT3G56960lAT3G56640lAT2G13680lAT3G51300lAT3G01700lAT1G79250
10014	1.12E-03	1.02E-02	4	21	502	22304	meristem initiation	AT1G11130lAT2G20000lAT2G34710lAT5G60690
10558	1.17E-03	1.05E-02	7	72	502	22304	negative regulation of macromolecule biosynthetic process	AT3G02000lAT4G34590lAT4G38130lAT5G55760lAT1G63020lAT3G02820lAT2G35160

<table>
<tr><td>9987</td><td>1.18E-03</td><td>1.05E-02</td><td>199</td><td>7393</td><td>502</td><td>22304</td><td>cellular process</td><td>AT1G44900|AT1G64630|AT5G67270|AT4G21060|AT1G08130|AT2G46180|AT5G64200|AT1G70510|AT2G16640|AT2G35160|AT5G06670|AT1G75640|AT3G51740|AT1G07270|AT1G09450|AT5G43990|AT3G56640|AT1G16680|AT3G60100|AT3G20670|AT5G64670|AT5G27240|AT4G27180|AT2G03210|AT4G16130|AT2G24490|AT3G45280|AT2G32670|AT5G51940|AT3G54670|AT3G06030|AT5G49500|AT2G36350|AT3G01700|AT4G22970|AT1G52500|AT2G20000|AT1G01510|AT1G06520|AT3G57830|AT5G16690|AT3G02000|AT1G31360|AT4G04930|AT5G65420|AT4G02070|AT4G29130|AT5G62390|AT5G08170|AT5G35520|AT3G12160|AT4G34160|AT1G75310|AT3G10540|AT2G42120|AT1G12430|AT5G47100|AT5G14380|AT1G55200|AT5G18620|AT3G54250|AT5G65450|AT2G34790|AT2G16440|AT3G20475|AT5G13960|AT5G55760|AT1G27120|AT2G40550|AT3G51300|AT5G58230|AT2G40790|AT1G80070|AT4G29830|AT2G31970|AT3G53730|AT5G15460|AT3G51310|AT5G24970|AT3G04680|AT3G02820|AT5G62290|AT3G12060|AT2G36190|AT4G14150|AT3G28780|AT3G53380|AT5G08110|AT4G20910|AT4G29170|AT1G04020|AT4G31360|AT1G11570|AT2G35630|AT5G46280|AT5G60690|AT4G02150|AT1G74420|AT5G63960|AT3G20260|AT1G08730|AT2G28590|AT1G63020|AT5G22880|AT3G23670|AT1G04050|AT1G57820|AT2G16360|AT1G25540|AT5G13000|AT3G20150|AT2G44830|AT4G35500|AT5G23720|AT1G07790|AT3G25165|AT4G05520|AT5G44200|AT2G19620|AT1G48410|AT5G62410|AT3G07880|AT3G24660|AT1G18550|AT3G04810|AT5G28290|AT4G30870|AT1G78770|AT1G11130|AT3G49250|AT5G23940|AT3G56100|AT5G61460|AT5G67200|AT5G67320|AT5G10400|AT4G05190|AT5G64610|AT3G12680|AT1G18450|AT1G52570|AT4G36180|AT3G18660|AT2G41550|AT4G35650|AT3G55620|AT5G18580|AT5G43020|AT1G33410|AT5G15630|AT4G02460|AT3G19090|AT5G09500|AT5G56330|AT4G38130|AT4G37280|AT5G11020|AT1G54340|AT5G51560|AT3G51030|AT5G47750|AT4G26840|AT1G08560|AT1G80350|AT3G56960|AT2G13680|AT3G59550|AT3G63130|AT2G23800|AT1G76540|AT1G03780|AT4G39110|AT1G79250|AT5G56580|AT1G61860|AT1G62950|AT2G39820|AT1G12370|AT2G40930|AT4G24790|AT5G57320|AT3G16150|AT1G79820|AT1G70210|AT2G01120|AT2G27990|AT5G66150|AT3G02680|AT2G32765|AT3G59530|AT5G23580|AT2G36200|AT1G11730|AT2G18230|AT1G73850|AT4G17483|AT2G23380|AT5G51350|AT2G24230</td></tr>
<tr><td>6268</td><td>1.21E-03</td><td>1.06E-02</td><td>3</td><td>10</td><td>502</td><td>22304</td><td>DNA unwinding involved in replication</td><td>AT1G44900|AT5G46280|AT2G16440</td></tr>
<tr><td>51510</td><td>1.21E-03</td><td>1.06E-02</td><td>3</td><td>10</td><td>502</td><td>22304</td><td>regulation of unidimensional cell growth</td><td>AT3G12160|AT2G20000|AT2G13680</td></tr>
<tr><td>7276</td><td>1.34E-03</td><td>1.16E-02</td><td>4</td><td>22</td><td>502</td><td>22304</td><td>gamete generation</td><td>AT2G20000|AT1G18450|AT1G06520|AT4G37750</td></tr>
<tr><td>40008</td><td>1.44E-03</td><td>1.23E-02</td><td>6</td><td>55</td><td>502</td><td>22304</td><td>regulation of growth</td><td>AT1G44900|AT3G12160|AT2G20000|AT4G28190|AT2G13680|AT5G13290</td></tr>
<tr><td>51225</td><td>1.49E-03</td><td>1.26E-02</td><td>2</td><td>3</td><td>502</td><td>22304</td><td>spindle assembly</td><td>AT4G05190|AT1G03780</td></tr>
<tr><td>6312</td><td>1.49E-03</td><td>1.26E-02</td><td>2</td><td>3</td><td>502</td><td>22304</td><td>mitotic recombination</td><td>AT2G31970|AT4G30870</td></tr>
<tr><td>48519</td><td>1.54E-03</td><td>1.27E-02</td><td>18</td><td>362</td><td>502</td><td>22304</td><td>negative regulation of biological process</td><td>AT3G49250|AT5G48820|AT4G29830|AT3G02000|AT1G63020|AT5G44200|AT3G02820|AT2G35160|AT3G19590|AT2G33560|AT4G20910|AT4G34590|AT4G38130|AT5G55760|AT1G48410|AT1G77470|AT2G24490|AT2G27990</td></tr>
<tr><td>48645</td><td>1.54E-03</td><td>1.27E-02</td><td>5</td><td>38</td><td>502</td><td>22304</td><td>organ formation</td><td>AT4G20910|AT3G02000|AT2G20300|AT1G70510|AT2G27990</td></tr>
<tr><td>31327</td><td>1.61E-03</td><td>1.30E-02</td><td>7</td><td>76</td><td>502</td><td>22304</td><td>negative regulation of cellular biosynthetic process</td><td>AT3G02000|AT4G34590|AT4G38130|AT5G55760|AT1G63020|AT3G02820|AT2G35160</td></tr>
<tr><td>9890</td><td>1.61E-03</td><td>1.30E-02</td><td>7</td><td>76</td><td>502</td><td>22304</td><td>negative regulation of biosynthetic process</td><td>AT3G02000|AT4G34590|AT4G38130|AT5G55760|AT1G63020|AT3G02820|AT2G35160</td></tr>
</table>

6270	1.63E-03	1.31E-02	3	11	502	22304	DNA-dependent DNA replication initiation	AT1G44900IAT5G46280IAT2G16440
7166	1.68E-03	1.34E-02	9	121	502	22304	cell surface receptor linked signaling pathway	AT3G57830IAT5G43020IAT1G62950IAT3G56100IAT4G36180IAT3G24660IAT1G75640I AT5G51350IAT3G51740
10154	1.82E-03	1.44E-02	21	459	502	22304	fruit development	AT1G11130IAT3G55620IAT5G23940IAT1G13120IAT5G40480IAT2G34710IAT5G27740I AT3G59420IAT4G22970IAT1G19850IAT2G34790IAT4G02460IAT5G08170IAT2G35340I AT2G20000IAT2G20300IAT1G48410IAT2G03850IAT3G07060IAT5G58230IAT4G13230
9892	1.84E-03	1.44E-02	12	198	502	22304	negative regulation of metabolic process	AT3G49250IAT4G20910IAT3G02000IAT4G34590IAT4G38130IAT5G55760IAT1G63020I AT1G48410IAT5G44200IAT3G02820IAT2G24490IAT2G35160
7059	1.88E-03	1.46E-02	4	24	502	22304	chromosome segregation	AT3G54670IAT2G40550IAT5G61460IAT4G22970
65004	1.90E-03	1.46E-02	6	58	502	22304	protein-DNA complex assembly	AT1G07790IAT3G53730IAT5G22880IAT3G20670IAT1G57820IAT5G10400
9908	2.08E-03	1.59E-02	13	228	502	22304	flower development	AT1G11130IAT3G02000IAT2G34710IAT1G59640IAT3G04680IAT5G24330IAT3G22990I AT1G70510IAT1G30330IAT1G19850IAT4G20910IAT4G28190IAT5G37020
22604	2.14E-03	1.62E-02	3	12	502	22304	regulation of cell morphogenesis	AT3G12160IAT2G20000IAT2G13680
6139	2.17E-03	1.63E-02	40	1104	502	22304	nucleobase, nucleoside, nucleotide and nucleic acid metabolic process	AT1G44900IAT5G51940IAT1G12370IAT1G63020IAT1G08130IAT5G64200IAT1G57820I AT2G35160IAT4G02460IAT2G16440IAT3G19090IAT4G24790IAT1G52500IAT3G20475I AT4G20910IAT5G13960IAT4G29170IAT1G04020IAT2G40550IAT4G30870IAT2G01120I AT5G58230IAT1G80070IAT1G07270IAT3G49250IAT3G02680IAT5G16690IAT1G31360I AT2G31970IAT3G04680IAT5G44200IAT5G46280IAT3G02820IAT5G61460IAT4G02070I AT5G63960IAT2G23380IAT2G24490IAT2G41550IAT2G42120
33043	2.20E-03	1.64E-02	4	25	502	22304	regulation of organelle organization	AT3G51300IAT3G19590IAT4G22970IAT2G33560
48589	2.26E-03	1.67E-02	12	203	502	22304	developmental growth	AT3G12160IAT5G14380IAT5G18580IAT3G07880IAT3G56960IAT3G56640IAT2G13680I AT3G51300IAT3G01700IAT4G22970IAT1G79250IAT1G01510
9909	2.43E-03	1.78E-02	8	104	502	22304	regulation of flower development	AT1G25540IAT4G22140IAT4G29830IAT4G20910IAT4G28190IAT5G13290IAT5G58230I AT4G02560
48509	2.46E-03	1.79E-02	6	61	502	22304	regulation of meristem development	AT1G44900IAT1G76540IAT4G28190IAT1G04020IAT1G27360IAT5G13290
42127	2.56E-03	1.84E-02	4	26	502	22304	regulation of cell proliferation	AT1G44900IAT4G34160IAT3G50070IAT4G37750
7062	2.74E-03	1.91E-02	3	13	502	22304	sister chromatid cohesion	AT3G54670IAT2G40550IAT5G61460
32506	2.74E-03	1.91E-02	3	13	502	22304	cytokinetic process	AT2G35630IAT3G23670IAT4G14150
45787	2.94E-03	1.91E-02	2	4	502	22304	positive regulation of cell cycle	AT3G50410IAT5G58230
45841	2.94E-03	1.91E-02	2	4	502	22304	negative regulation of mitotic metaphase/anaphase transition	AT3G19590IAT2G33560
31507	2.94E-03	1.91E-02	2	4	502	22304	heterochromatin formation	AT1G57820IAT5G58230
31577	2.94E-03	1.91E-02	2	4	502	22304	spindle checkpoint	AT3G19590IAT2G33560
7051	2.94E-03	1.91E-02	2	4	502	22304	spindle organization	AT4G05190IAT1G03780
7094	2.94E-03	1.91E-02	2	4	502	22304	mitotic cell cycle spindle assembly checkpoint	AT3G19590IAT2G33560
70828	2.94E-03	1.91E-02	2	4	502	22304	heterochromatin organization	AT1G57820IAT5G58230
71173	2.94E-03	1.91E-02	2	4	502	22304	spindle assembly checkpoint	AT3G19590IAT2G33560
71174	2.94E-03	1.91E-02	2	4	502	22304	mitotic cell cycle spindle checkpoint	AT3G19590IAT2G33560
9855	2.94E-03	1.91E-02	2	4	502	22304	determination of bilateral symmetry	AT2G34710IAT5G60690
10071	2.94E-03	1.91E-02	2	4	502	22304	root meristem specification	AT1G11130IAT2G20000
16571	2.95E-03	1.91E-02	4	27	502	22304	histone methylation	AT4G29830IAT5G13960IAT2G23380IAT2G35160

48468	2.99E-03	1.92E-02	11	183	502	22304	cell development	AT3G12160|AT5G23940|AT5G14380|AT1G80350|AT3G56960|AT3G56640|AT2G13680|AT3G51300|AT3G01700|AT1G79250|AT2G27990
16049	3.24E-03	2.07E-02	13	240	502	22304	cell growth	AT5G14380|AT5G18580|AT3G07880|AT1G80350|AT3G56960|AT3G56640|AT2G13680|AT3G01700|AT4G22970|AT3G12160|AT3G51300|AT1G79250|AT1G01510
43622	3.43E-03	2.13E-02	3	14	502	22304	cortical microtubule organization	AT5G23720|AT1G80350|AT3G06030
45786	3.43E-03	2.13E-02	3	14	502	22304	negative regulation of cell cycle	AT5G48820|AT3G19590|AT2G33560
32392	3.43E-03	2.13E-02	3	14	502	22304	DNA geometric change	AT1G44900|AT5G46280|AT2G16440
32508	3.43E-03	2.13E-02	3	14	502	22304	DNA duplex unwinding	AT1G44900|AT5G46280|AT2G16440
48444	3.85E-03	2.36E-02	4	29	502	22304	floral organ morphogenesis	AT4G20910|AT3G02000|AT1G59640|AT1G70510
48563	3.85E-03	2.36E-02	4	29	502	22304	post-embryonic organ morphogenesis	AT4G20910|AT3G02000|AT1G59640|AT1G70510
22621	3.94E-03	2.40E-02	15	304	502	22304	shoot system development	AT1G11130|AT5G23940|AT2G34710|AT1G80350|AT3G04680|AT3G22990|AT5G13300|AT2G20300|AT1G48410|AT5G28640|AT1G04020|AT2G23380|AT1G01510|AT5G58230|AT2G27990
48523	4.13E-03	2.49E-02	11	191	502	22304	negative regulation of cellular process	AT5G48820|AT3G02000|AT4G34590|AT4G38130|AT5G55760|AT1G63020|AT3G02820|AT2G35160|AT3G19590|AT2G33560|AT2G27990
6298	4.21E-03	2.49E-02	3	15	502	22304	mismatch repair	AT3G20475|AT4G02070|AT4G02460
6338	4.21E-03	2.49E-02	3	15	502	22304	chromatin remodeling	AT5G18620|AT1G57820|AT5G58230
6476	4.21E-03	2.49E-02	3	15	502	22304	protein amino acid deacetylation	AT4G38130|AT5G55760|AT5G67320
31122	4.21E-03	2.49E-02	3	15	502	22304	cytoplasmic microtubule organization	AT5G23720|AT1G80350|AT3G06030
7389	4.28E-03	2.51E-02	8	114	502	22304	pattern specification process	AT4G20910|AT5G13300|AT2G34710|AT1G02800|AT5G60690|AT1G70510|AT1G19850|AT2G27990
10629	4.47E-03	2.60E-02	10	166	502	22304	negative regulation of gene expression	AT3G49250|AT4G20910|AT3G02000|AT4G38130|AT5G55760|AT1G63020|AT1G48410|AT5G44200|AT2G24490|AT2G35160
9856	4.71E-03	2.61E-02	9	141	502	22304	pollination	AT3G12160|AT5G14380|AT3G56960|AT3G56640|AT2G13680|AT5G19610|AT3G51300|AT3G01700|AT1G79250
31324	4.75E-03	2.61E-02	7	92	502	22304	negative regulation of cellular metabolic process	AT3G02000|AT4G34590|AT4G38130|AT5G55760|AT1G63020|AT3G02820|AT2G35160
80092	4.83E-03	2.61E-02	2	5	502	22304	regulation of pollen tube growth	AT3G12160|AT2G13680
6467	4.83E-03	2.61E-02	2	5	502	22304	protein thiol-disulfide exchange	AT3G02000|AT2G40790
10769	4.83E-03	2.61E-02	2	5	502	22304	regulation of cell morphogenesis involved in differentiation	AT3G12160|AT2G13680
51784	4.83E-03	2.61E-02	2	5	502	22304	negative regulation of nuclear division	AT3G19590|AT2G33560
45839	4.83E-03	2.61E-02	2	5	502	22304	negative regulation of mitosis	AT3G19590|AT2G33560
60284	4.83E-03	2.61E-02	2	5	502	22304	regulation of cell development	AT3G12160|AT2G13680
7093	4.83E-03	2.61E-02	2	5	502	22304	mitotic cell cycle checkpoint	AT3G19590|AT2G33560
70925	4.83E-03	2.61E-02	2	5	502	22304	organelle assembly	AT4G05190|AT1G03780
30071	4.83E-03	2.61E-02	2	5	502	22304	regulation of mitotic metaphase/anaphase transition	AT3G19590|AT2G33560
9799	4.83E-03	2.61E-02	2	5	502	22304	specification of symmetry	AT2G34710|AT5G60690
8361	5.21E-03	2.80E-02	13	254	502	22304	regulation of cell size	AT5G14380|AT5G18580|AT3G07880|AT1G80350|AT3G56960|AT3G56640|AT2G13680|AT3G01700|AT4G22970|AT3G12160|AT3G51300|AT1G79250|AT1G01510

6468	5.28E-03	2.82E-02	31	842	502	22304	protein amino acid phosphorylation	AT1G64630lAT1G61860lAT2G28590lAT5G43020lAT1G62950lAT3G24660lAT2G36350lAT1G55200lAT3G04810lAT3G53380lAT5G28290lAT1G75640lAT5G11020lAT5G51560lAT2G44830lAT3G51740lAT4G35500lAT3G57830lAT5G47750lAT5G24970lAT3G56100lAT1G09450lAT5G23580lAT5G67200lAT1G76540lAT4G36180lAT3G10540lAT5G51350lAT4G39110lAT5G56580lAT2G24230
32535	5.38E-03	2.84E-02	13	255	502	22304	regulation of cellular component size	AT5G14380lAT5G18580lAT3G07880lAT1G80350lAT3G56960lAT3G56640lAT2G13680lAT3G01700lAT4G22970lAT3G12160lAT3G51300lAT1G79250lAT1G01510
90066	5.38E-03	2.84E-02	13	255	502	22304	regulation of anatomical structure size	AT5G14380lAT5G18580lAT3G07880lAT1G80350lAT3G56960lAT3G56640lAT2G13680lAT3G01700lAT4G22970lAT3G12160lAT3G51300lAT1G79250lAT1G01510
9846	5.53E-03	2.89E-02	4	32	502	22304	pollen germination	AT3G56960lAT3G56640lAT2G13680lAT5G19610
6355	5.54E-03	2.89E-02	30	810	502	22304	regulation of transcription, DNA-dependent	AT5G22760lAT1G35490lAT2G34710lAT1G63020lAT1G31150lAT5G20240lAT2G13570lAT1G57820lAT2G35160lAT4G34590lAT4G14920lAT5G55760lAT2G45460lAT4G34990lAT3G06740lAT4G22140lAT2G35310lAT4G31610lAT3G05670lAT5G24330lAT5G60690lAT1G30330lAT4G22680lAT3G61250lAT5G09790lAT3G14980lAT5G37020lAT3G46770lAT1G64800lAT4G37750
45934	5.63E-03	2.90E-02	6	72	502	22304	negative regulation of nucleobase, nucleoside, nucleotide and nucleic acid metabolic process	AT3G02000lAT4G38130lAT5G55760lAT1G63020lAT3G02820lAT2G35160
51172	5.63E-03	2.90E-02	6	72	502	22304	negative regulation of nitrogen compound metabolic process	AT3G02000lAT4G38130lAT5G55760lAT1G63020lAT3G02820lAT2G35160
51252	5.83E-03	2.99E-02	30	813	502	22304	regulation of RNA metabolic process	AT5G22760lAT1G35490lAT2G34710lAT1G63020lAT1G31150lAT5G20240lAT2G13570lAT1G57820lAT2G35160lAT4G34590lAT4G14920lAT5G55760lAT2G45460lAT4G34990lAT3G06740lAT4G22140lAT2G35310lAT4G31610lAT3G05670lAT5G24330lAT5G60690lAT1G30330lAT4G22680lAT3G61250lAT5G09790lAT3G14980lAT5G37020lAT3G46770lAT1G64800lAT4G37750
43414	6.02E-03	3.07E-02	6	73	502	22304	macromolecule methylation	AT3G49250lAT4G29830lAT5G13960lAT2G23380lAT1G57820lAT2G35160
87	6.09E-03	3.07E-02	3	17	502	22304	M phase of mitotic cell cycle	AT5G35520lAT2G35630lAT3G59550
7067	6.09E-03	3.07E-02	3	17	502	22304	mitosis	AT5G35520lAT2G35630lAT3G59550
16458	6.11E-03	3.07E-02	8	121	502	22304	gene silencing	AT3G49250lAT4G20910lAT5G55760lAT1G63020lAT1G48410lAT5G44200lAT2G24490lAT2G35160
6730	6.33E-03	3.16E-02	7	97	502	22304	one-carbon metabolic process	AT3G49250lAT4G29830lAT5G13960lAT5G56330lAT2G23380lAT1G57820lAT2G35160
8213	6.89E-03	3.34E-02	4	34	502	22304	protein amino acid alkylation	AT4G29830lAT5G13960lAT2G23380lAT2G35160
6305	6.89E-03	3.34E-02	4	34	502	22304	DNA alkylation	AT3G49250lAT5G13960lAT1G57820lAT2G35160
6306	6.89E-03	3.34E-02	4	34	502	22304	DNA methylation	AT3G49250lAT5G13960lAT1G57820lAT2G35160
6479	6.89E-03	3.34E-02	4	34	502	22304	protein amino acid methylation	AT4G29830lAT5G13960lAT2G23380lAT2G35160
10015	7.06E-03	3.34E-02	7	99	502	22304	root morphogenesis	AT1G11130lAT1G44900lAT2G20000lAT3G07880lAT3G59420lAT5G13290lAT3G04630
31032	7.14E-03	3.34E-02	2	6	502	22304	actomyosin structure organization	AT3G23670lAT4G14150
51783	7.14E-03	3.34E-02	2	6	502	22304	regulation of nuclear division	AT3G19590lAT2G33560
912	7.14E-03	3.34E-02	2	6	502	22304	assembly of actomyosin apparatus involved in cell cycle cytokinesis	AT3G23670lAT4G14150
914	7.14E-03	3.34E-02	2	6	502	22304	phragmoplast assembly	AT3G23670lAT4G14150
7088	7.14E-03	3.34E-02	2	6	502	22304	regulation of mitosis	AT3G19590lAT2G33560

<table>
<tr><td>48446</td><td>7.14E-03</td><td>3.34E-02</td><td>2</td><td>6</td><td>502</td><td>22304</td><td>petal morphogenesis</td><td>AT3G02000|AT1G59640</td></tr>
<tr><td>10082</td><td>7.14E-03</td><td>3.34E-02</td><td>2</td><td>6</td><td>502</td><td>22304</td><td>regulation of root meristem growth</td><td>AT1G44900|AT5G13290</td></tr>
<tr><td>6342</td><td>7.19E-03</td><td>3.35E-02</td><td>3</td><td>18</td><td>502</td><td>22304</td><td>chromatin silencing</td><td>AT5G55760|AT1G63020|AT2G35160</td></tr>
<tr><td>6304</td><td>7.64E-03</td><td>3.54E-02</td><td>4</td><td>35</td><td>502</td><td>22304</td><td>DNA modification</td><td>AT3G49250|AT5G13960|AT1G57820|AT2G35160</td></tr>
<tr><td>32259</td><td>7.78E-03</td><td>3.57E-02</td><td>6</td><td>77</td><td>502</td><td>22304</td><td>methylation</td><td>AT3G49250|AT4G29830|AT5G13960|AT2G23380|AT1G57820|AT2G35160</td></tr>
<tr><td>6334</td><td>7.81E-03</td><td>3.57E-02</td><td>5</td><td>55</td><td>502</td><td>22304</td><td>nucleosome assembly</td><td>AT1G07790|AT3G53730|AT5G22880|AT3G20670|AT5G10400</td></tr>
<tr><td>34728</td><td>7.81E-03</td><td>3.57E-02</td><td>5</td><td>55</td><td>502</td><td>22304</td><td>nucleosome organization</td><td>AT1G07790|AT3G53730|AT5G22880|AT3G20670|AT5G10400</td></tr>
<tr><td>70192</td><td>8.40E-03</td><td>3.73E-02</td><td>3</td><td>19</td><td>502</td><td>22304</td><td>chromosome organization involved in meiosis</td><td>AT3G02680|AT3G20475|AT4G30870</td></tr>
<tr><td>911</td><td>8.40E-03</td><td>3.73E-02</td><td>3</td><td>19</td><td>502</td><td>22304</td><td>cytokinesis by cell plate formation</td><td>AT2G35630|AT3G23670|AT4G14150</td></tr>
<tr><td>7129</td><td>8.40E-03</td><td>3.73E-02</td><td>3</td><td>19</td><td>502</td><td>22304</td><td>synapsis</td><td>AT3G02680|AT3G20475|AT4G30870</td></tr>
<tr><td>1558</td><td>8.40E-03</td><td>3.73E-02</td><td>3</td><td>19</td><td>502</td><td>22304</td><td>regulation of cell growth</td><td>AT3G12160|AT2G20000|AT2G13680</td></tr>
<tr><td>48367</td><td>8.42E-03</td><td>3.73E-02</td><td>14</td><td>300</td><td>502</td><td>22304</td><td>shoot development</td><td>AT5G23940|AT2G34710|AT1G80350|AT3G04680|AT3G22990|AT5G13300|AT2G20300|AT1G48410|AT5G28640|AT1G04020|AT2G23380|AT1G01510|AT5G58230|AT2G27990</td></tr>
<tr><td>48609</td><td>8.42E-03</td><td>3.73E-02</td><td>5</td><td>56</td><td>502</td><td>22304</td><td>reproductive process in a multicellular organism</td><td>AT2G20000|AT1G18450|AT5G24330|AT1G06520|AT4G37750</td></tr>
<tr><td>33554</td><td>8.77E-03</td><td>3.85E-02</td><td>16</td><td>364</td><td>502</td><td>22304</td><td>cellular response to stress</td><td>AT1G31360|AT2G31970|AT1G12370|AT1G08130|AT3G02820|AT5G61460|AT4G02070|AT5G62390|AT4G02460|AT1G52500|AT3G20475|AT4G29170|AT1G04020|AT2G40550|AT2G24490|AT4G30870</td></tr>
<tr><td>44260</td><td>8.78E-03</td><td>3.85E-02</td><td>103</td><td>3667</td><td>502</td><td>22304</td><td>cellular macromolecule metabolic process</td><td>AT1G44900|AT1G64630|AT4G21060|AT2G28590|AT1G63020|AT1G08130|AT5G64200|AT1G57820|AT2G35160|AT2G16360|AT5G13000|AT1G75640|AT2G44830|AT3G51740|AT4G35500|AT1G07270|AT1G09450|AT5G44200|AT1G16680|AT5G64670|AT5G27240|AT2G24490|AT5G51940|AT3G24660|AT2G36350|AT3G04810|AT5G28290|AT1G52500|AT4G30870|AT3G57830|AT3G49250|AT5G16690|AT3G02000|AT1G31360|AT3G56100|AT5G61460|AT4G02070|AT5G67200|AT5G62390|AT5G67320|AT4G36180|AT3G18660|AT1G75310|AT3G10540|AT2G41550|AT2G42120|AT3G55620|AT5G43020|AT1G55200|AT5G65450|AT4G02460|AT2G16440|AT3G19090|AT3G20475|AT5G09500|AT5G13960|AT4G38130|AT5G55760|AT1G27120|AT5G11020|AT2G40550|AT5G51560|AT2G40790|AT1G80070|AT5G47750|AT4G29830|AT4G26840|AT2G31970|AT5G15460|AT5G24970|AT3G04680|AT3G02820|AT2G13680|AT3G12060|AT1G76540|AT2G36190|AT4G39110|AT5G56580|AT1G61860|AT1G62950|AT2G39820|AT1G12370|AT2G40930|AT3G53380|AT4G24790|AT5G08110|AT4G20910|AT3G16150|AT4G29170|AT1G04020|AT2G01120|AT3G02680|AT2G32765|AT5G46280|AT5G23580|AT1G11730|AT1G73850|AT5G63960|AT3G20260|AT4G17483|AT2G23380|AT5G51350|AT2G24230</td></tr>
<tr><td>16310</td><td>8.86E-03</td><td>3.87E-02</td><td>32</td><td>910</td><td>502</td><td>22304</td><td>phosphorylation</td><td>AT1G64630|AT1G61860|AT2G28590|AT5G43020|AT1G62950|AT3G24660|AT2G36350|AT1G55200|AT3G04810|AT3G54250|AT3G53380|AT5G28290|AT1G75640|AT5G11020|AT5G51560|AT2G44830|AT3G51740|AT4G35500|AT3G57830|AT5G47750|AT5G24970|AT3G56100|AT1G09450|AT5G23580|AT5G67200|AT1G76540|AT4G36180|AT3G10540|AT5G51350|AT4G39110|AT5G56580|AT2G24230</td></tr>
<tr><td>9933</td><td>9.31E-03</td><td>4.03E-02</td><td>4</td><td>37</td><td>502</td><td>22304</td><td>meristem structural organization</td><td>AT1G11130|AT2G20000|AT2G34710|AT5G60690</td></tr>
<tr><td>10016</td><td>9.32E-03</td><td>4.03E-02</td><td>9</td><td>157</td><td>502</td><td>22304</td><td>shoot morphogenesis</td><td>AT5G23940|AT5G13300|AT2G34710|AT1G48410|AT1G80350|AT2G23380|AT1G01510|AT5G58230|AT2G27990</td></tr>
</table>

10927	9.72E-03	4.10E-02	3	20	502	22304	cellular component assembly involved in morphogenesis	AT3G28780IAT2G13680IAT3G59530
10208	9.72E-03	4.10E-02	3	20	502	22304	pollen wall assembly	AT3G28780IAT2G13680IAT3G59530
75	9.85E-03	4.10E-02	2	7	502	22304	cell cycle checkpoint	AT3G19590IAT2G33560
35196	9.85E-03	4.10E-02	2	7	502	22304	production of miRNAs involved in gene silencing by miRNA	AT4G20910IAT5G44200
52018	9.85E-03	4.10E-02	2	7	502	22304	modulation by symbiont of RNA levels in host	AT4G20910IAT1G48410
52249	9.85E-03	4.10E-02	2	7	502	22304	modulation of RNA levels in other organism involved in symbiotic interaction	AT4G20910IAT1G48410
9616	9.85E-03	4.10E-02	2	7	502	22304	virus induced gene silencing	AT4G20910IAT1G48410
10216	9.85E-03	4.10E-02	2	7	502	22304	maintenance of DNA methylation	AT5G13960IAT2G35160
40007	1.10E-02	4.57E-02	13	279	502	22304	growth	AT5G14380IAT5G18580IAT3G07880IAT1G80350IAT3G56960IAT3G56640IAT2G13680IAT3G01700IAT4G22970IAT3G12160IAT3G51300IAT1G79250IAT1G01510
280	1.11E-02	4.58E-02	3	21	502	22304	nuclear division	AT5G35520IAT2G35630IAT3G59550
48449	1.11E-02	4.58E-02	3	21	502	22304	floral organ formation	AT4G20910IAT3G02000IAT1G70510
32504	1.12E-02	4.58E-02	5	60	502	22304	multicellular organism reproduction	AT2G20000IAT1G18450IAT5G24330IAT1G06520IAT4G37750
16481	1.20E-02	4.86E-02	5	61	502	22304	negative regulation of transcription	AT3G02000IAT4G38130IAT5G55760IAT1G63020IAT2G35160
51321	1.20E-02	4.86E-02	5	61	502	22304	meiotic cell cycle	AT3G02680IAT3G20475IAT2G13680IAT4G30870IAT4G22970
48827	1.22E-02	4.92E-02	11	222	502	22304	phyllome development	AT5G23940IAT5G13300IAT1G48410IAT1G80350IAT3G04680IAT5G28640IAT1G04020IAT3G22990IAT2G23380IAT1G01510IAT5G58230

Supplementary Table S6 – C) Functional categories represented by AuxRE motif possessing co-expressed genes of *ARF6-ARF8* analyzed by BiNGO

<table>
<tr><th>GO-ID</th><th>p-value</th><th>corr p-value</th><th>x</th><th>n</th><th>X</th><th>N</th><th>Description</th><th>Genes in test set</th></tr>
<tr><td>7166</td><td>2.63E-07</td><td>9.04E-05</td><td>11</td><td>121</td><td>270</td><td>22304</td><td>cell surface receptor linked signaling pathway</td><td>AT3G57830|AT3G49670|AT5G43020|AT1G62950|AT3G24660|AT4G36180|AT5G14210|AT3G51740|AT5G51350|AT1G27190|AT5G57690</td></tr>
<tr><td>7169</td><td>3.80E-07</td><td>9.04E-05</td><td>10</td><td>100</td><td>270</td><td>22304</td><td>transmembrane receptor protein tyrosine kinase signaling pathway</td><td>AT3G57830|AT3G49670|AT5G43020|AT1G62950|AT3G24660|AT4G36180|AT5G14210|AT3G51740|AT5G51350|AT1G27190</td></tr>
<tr><td>7167</td><td>3.80E-07</td><td>9.04E-05</td><td>10</td><td>100</td><td>270</td><td>22304</td><td>enzyme linked receptor protein signaling pathway</td><td>AT3G57830|AT3G49670|AT5G43020|AT1G62950|AT3G24660|AT4G36180|AT5G14210|AT3G51740|AT5G51350|AT1G27190</td></tr>
<tr><td>50789</td><td>5.28E-07</td><td>9.40E-05</td><td>63</td><td>2783</td><td>270</td><td>22304</td><td>regulation of biological process</td><td>AT1G44900|AT1G59640|AT4G31820|AT1G63100|AT1G31150|AT5G65410|AT3G59420|AT1G57820|AT4G00480|AT1G49480|AT5G66940|AT4G35700|AT1G75240|AT5G58230|AT3G49670|AT5G23400|AT2G13680|AT4G21750|AT1G30330|AT5G66770|AT5G09790|AT1G48410|AT1G76540|AT1G77470|AT2G24490|AT5G13290|AT1G35490|AT4G18390|AT3G25670|AT1G70895|AT3G50070|AT4G31805|AT2G33560|AT5G08550|AT4G20910|AT1G18040|AT1G74850|AT3G63300|AT1G70210|AT3G06160|AT1G78770|AT4G31360|AT2G27990|AT1G61110|AT1G11130|AT3G49250|AT2G35310|AT1G60860|AT4G31610|AT1G27360|AT5G60690|AT4G23750|AT4G34160|AT3G61250|AT1G16070|AT3G42670|AT2G26330|AT5G37020|AT5G05610|AT4G14770|AT2G28550|AT4G37750|AT5G57690</td></tr>
<tr><td>9887</td><td>1.89E-06</td><td>2.03E-04</td><td>12</td><td>178</td><td>270</td><td>22304</td><td>organ morphogenesis</td><td>AT4G20910|AT5G23940|AT5G13300|AT1G59640|AT1G48410|AT4G18390|AT2G26330|AT1G70510|AT5G58230|AT2G27990|AT2G28550|AT4G37750</td></tr>
<tr><td>6260</td><td>2.12E-06</td><td>2.03E-04</td><td>9</td><td>94</td><td>270</td><td>22304</td><td>DNA replication</td><td>AT5G08550|AT1G44900|AT5G16690|AT5G63960|AT5G41880|AT2G24490|AT2G16440|AT4G24790|AT2G42120</td></tr>
<tr><td>48513</td><td>2.49E-06</td><td>2.03E-04</td><td>25</td><td>719</td><td>270</td><td>22304</td><td>organ development</td><td>AT1G44900|AT1G59640|AT4G31820|AT4G18390|AT3G59420|AT1G70510|AT4G20910|AT5G13300|AT1G23380|AT5G28640|AT1G78770|AT5G58230|AT2G27990|AT1G11130|AT5G23940|AT3G49670|AT5G60690|AT4G21750|AT1G19850|AT4G23750|AT1G48410|AT2G26330|AT5G13290|AT2G28550|AT4G37750</td></tr>
<tr><td>48731</td><td>2.55E-06</td><td>2.03E-04</td><td>25</td><td>720</td><td>270</td><td>22304</td><td>system development</td><td>AT1G44900|AT1G59640|AT4G31820|AT4G18390|AT3G59420|AT1G70510|AT4G20910|AT5G13300|AT1G23380|AT5G28640|AT1G78770|AT5G58230|AT2G27990|AT1G11130|AT5G23940|AT3G49670|AT5G60690|AT4G21750|AT1G19850|AT4G23750|AT1G48410|AT2G26330|AT5G13290|AT2G28550|AT4G37750</td></tr>
<tr><td>7049</td><td>2.56E-06</td><td>2.03E-04</td><td>11</td><td>152</td><td>270</td><td>22304</td><td>cell cycle</td><td>AT5G08550|AT4G05190|AT4G34160|AT3G63130|AT3G54670|AT1G76540|AT3G23670|AT1G70210|AT2G13680|AT1G78770|AT1G03780</td></tr>
<tr><td>32501</td><td>5.19E-06</td><td>3.70E-04</td><td>43</td><td>1732</td><td>270</td><td>22304</td><td>multicellular organismal process</td><td>AT1G44900|AT5G67270|AT5G14380|AT1G59640|AT3G23670|AT4G31820|AT4G18390|AT3G59420|AT5G19610|AT1G70510|AT2G35340|AT4G20910|AT5G13300|AT1G02205|AT1G02800|AT1G23380|AT5G28640|AT3G10570|AT1G78770|AT5G58230|AT2G27990|AT1G04520|AT1G61110|AT1G11130|AT2G33330|AT5G23940|AT3G49670|AT2G13680|AT5G60690|AT4G21750|AT1G19850|AT1G30330|AT4G23750|AT5G09790|AT1G48410|AT1G18450|AT2G26330|AT5G37020|AT3G52940|AT3G63290|AT5G13290|AT2G28550|AT4G37750</td></tr>
<tr><td>51726</td><td>8.42E-06</td><td>5.27E-04</td><td>9</td><td>111</td><td>270</td><td>22304</td><td>regulation of cell cycle</td><td>AT4G34160|AT5G09790|AT1G18040|AT1G76540|AT1G70210|AT1G78770|AT3G50070|AT5G58230|AT2G33560</td></tr>
<tr><td>50794</td><td>9.04E-06</td><td>5.27E-04</td><td>54</td><td>2448</td><td>270</td><td>22304</td><td>regulation of cellular process</td><td>AT1G44900|AT1G59640|AT1G63100|AT1G31150|AT5G65410|AT3G59420|AT1G57820|AT4G00480|AT1G49480|AT5G66940|AT4G35700|AT1G75240|AT5G58230|AT5G23400|AT2G13680|AT4G21750|AT1G30330|AT5G66770|AT5G09790|AT1G76540|AT1G35490|AT4G18390|AT3G25670|AT1G70895|AT3G50070|AT4G31805|AT2G33560|AT5G08550|AT1G18040|AT1G74850|AT3G63300|AT1G70210|AT3G06160|AT1G78770|AT4G31360|AT2G27990|AT1G61110|AT1G11130|AT2G35310|AT1G60860|AT4G31610|AT1G27360|AT5G60690|AT4G23750|AT4G34160|AT3G61250|AT1G16070|AT2G26330|AT5G37020|AT5G05610|AT4G14770|AT2G28550|AT4G37750|AT5G57690</td></tr>
</table>

<table>
<tr><td>3</td><td>9.61E-06</td><td>5.27E-04</td><td>28</td><td>931</td><td>270</td><td>22304</td><td>reproduction</td><td>AT5G14380|AT1G59640|AT4G31820|AT3G59420|AT5G19610|AT1G70510|AT2G35340|AT5G07560|AT4G20910|AT1G01370|AT1G19890|AT3G10570|AT5G58230|AT2G27990|AT1G11130|AT5G23940|AT3G49670|AT2G13680|AT4G21750|AT1G19850|AT1G30330|AT4G23750|AT1G48410|AT1G18450|AT5G37020|AT3G52940|AT2G28550|AT4G37750</td></tr>
<tr><td>22402</td><td>1.81E-05</td><td>7.75E-04</td><td>9</td><td>122</td><td>270</td><td>22304</td><td>cell cycle process</td><td>AT5G08550|AT4G05190|AT4G34160|AT3G63130|AT3G54670|AT1G76540|AT3G23670|AT1G70210|AT1G03780</td></tr>
<tr><td>22414</td><td>1.81E-05</td><td>7.75E-04</td><td>27</td><td>911</td><td>270</td><td>22304</td><td>reproductive process</td><td>AT5G14380|AT1G59640|AT4G31820|AT3G59420|AT5G19610|AT1G70510|AT2G35340|AT4G20910|AT1G01370|AT1G19890|AT3G10570|AT5G58230|AT2G27990|AT1G11130|AT5G23940|AT3G49670|AT2G13680|AT4G21750|AT1G19850|AT1G30330|AT4G23750|AT1G48410|AT1G18450|AT5G37020|AT3G52940|AT2G28550|AT4G37750</td></tr>
<tr><td>9653</td><td>1.86E-05</td><td>7.75E-04</td><td>19</td><td>515</td><td>270</td><td>22304</td><td>anatomical structure morphogenesis</td><td>AT1G11130|AT1G44900|AT5G23940|AT5G14380|AT1G59640|AT4G18390|AT3G59420|AT2G13680|AT5G60690|AT1G70510|AT4G20910|AT5G13300|AT1G48410|AT2G26330|AT5G58230|AT5G13290|AT2G27990|AT2G28550|AT4G37750</td></tr>
<tr><td>9888</td><td>1.86E-05</td><td>7.75E-04</td><td>13</td><td>260</td><td>270</td><td>22304</td><td>tissue development</td><td>AT1G11130|AT1G44900|AT5G23940|AT3G49670|AT5G60690|AT4G21750|AT1G19850|AT5G13300|AT1G23380|AT1G78770|AT5G58230|AT5G13290|AT2G27990</td></tr>
<tr><td>48507</td><td>2.04E-05</td><td>7.75E-04</td><td>8</td><td>95</td><td>270</td><td>22304</td><td>meristem development</td><td>AT1G11130|AT1G44900|AT3G49670|AT1G23380|AT5G60690|AT1G19850|AT5G13290|AT2G27990</td></tr>
<tr><td>65007</td><td>2.07E-05</td><td>7.75E-04</td><td>65</td><td>3243</td><td>270</td><td>22304</td><td>biological regulation</td><td>AT1G44900|AT5G14380|AT1G59640|AT4G31820|AT1G63100|AT1G31150|AT5G65410|AT3G59420|AT1G57820|AT4G00480|AT1G49480|AT5G66940|AT4G35700|AT1G75240|AT5G58230|AT3G49670|AT5G23400|AT2G13680|AT4G21750|AT1G30330|AT5G66770|AT5G09790|AT1G48410|AT1G76540|AT3G14240|AT1G77470|AT2G24490|AT5G13290|AT1G35490|AT4G18390|AT3G25670|AT1G70895|AT3G50070|AT4G31805|AT2G33560|AT5G08550|AT4G20910|AT1G18040|AT1G74850|AT3G63300|AT1G70210|AT3G06160|AT1G78770|AT4G31360|AT2G27990|AT1G61110|AT1G11130|AT3G49250|AT2G35310|AT1G60860|AT4G31610|AT1G27360|AT5G60690|AT4G23750|AT4G34160|AT3G61250|AT1G16070|AT3G42670|AT2G26330|AT5G37020|AT5G05610|AT4G14770|AT2G28550|AT4G37750|AT5G57690</td></tr>
<tr><td>6259</td><td>2.86E-05</td><td>1.02E-03</td><td>14</td><td>311</td><td>270</td><td>22304</td><td>DNA metabolic process</td><td>AT1G44900|AT3G49250|AT4G21070|AT5G16690|AT5G41880|AT1G57820|AT2G16440|AT4G24790|AT5G08550|AT3G10140|AT5G63960|AT2G24490|AT1G80850|AT2G42120</td></tr>
<tr><td>60255</td><td>3.23E-05</td><td>1.10E-03</td><td>40</td><td>1685</td><td>270</td><td>22304</td><td>regulation of macromolecule metabolic process</td><td>AT1G35490|AT1G59640|AT4G18390|AT1G63100|AT1G31150|AT5G65410|AT1G57820|AT4G00480|AT1G49480|AT4G31805|AT5G08550|AT4G20910|AT1G74850|AT5G66940|AT3G06160|AT4G35700|AT1G75240|AT1G78770|AT5G58230|AT1G61110|AT3G49250|AT2G35310|AT4G31610|AT1G27360|AT5G60690|AT4G21750|AT1G30330|AT5G66770|AT4G23750|AT3G61250|AT1G16070|AT5G09790|AT1G48410|AT3G42670|AT5G37020|AT5G05610|AT2G24490|AT4G14770|AT2G28550|AT4G37750</td></tr>
<tr><td>6468</td><td>3.64E-05</td><td>1.18E-03</td><td>25</td><td>842</td><td>270</td><td>22304</td><td>protein amino acid phosphorylation</td><td>AT5G43020|AT1G62950|AT3G24660|AT2G36350|AT1G55200|AT1G27190|AT1G49730|AT5G51560|AT3G51740|AT2G44830|AT3G57830|AT3G49670|AT1G09450|AT5G14210|AT5G67200|AT5G18910|AT2G26730|AT1G76540|AT4G36180|AT3G10540|AT5G51350|AT2G45590|AT1G60800|AT2G24230|AT4G34440</td></tr>
<tr><td>10468</td><td>4.05E-05</td><td>1.22E-03</td><td>39</td><td>1642</td><td>270</td><td>22304</td><td>regulation of gene expression</td><td>AT1G35490|AT1G59640|AT4G18390|AT1G63100|AT1G31150|AT5G65410|AT1G57820|AT4G00480|AT1G49480|AT4G31805|AT5G08550|AT4G20910|AT1G74850|AT5G66940|AT3G06160|AT4G35700|AT1G75240|AT5G58230|AT1G61110|AT3G49250|AT2G35310|AT4G31610|AT1G27360|AT5G60690|AT4G21750|AT1G30330|AT5G66770|AT4G23750|AT3G61250|AT1G16070|AT5G09790|AT1G48410|AT3G42670|AT5G37020|AT5G05610|AT2G24490|AT4G14770|AT2G28550|AT4G37750</td></tr>
</table>

<table>
<tr><td>19222</td><td>4.11E-05</td><td>1.22E-03</td><td>42</td><td>1825</td><td>270</td><td>22304</td><td>regulation of metabolic process</td><td>AT1G35490|AT1G59640|AT4G18390|AT1G63100|AT1G31150|AT5G65410|AT1G57820|AT4G00480|AT1G49480|AT4G31805|AT5G08550|AT4G20910|AT1G74850|AT5G66940|AT3G06160|AT4G35700|AT1G75240|AT1G78770|AT5G58230|AT1G61110|AT3G49250|AT2G35310|AT1G60860|AT4G31610|AT1G27360|AT5G60690|AT4G21750|AT1G30330|AT5G66770|AT4G23750|AT3G61250|AT1G16070|AT5G09790|AT1G48410|AT3G42670|AT5G37020|AT5G05610|AT2G24490|AT4G14770|AT2G28550|AT4G37750|AT5G57690</td></tr>
<tr><td>7275</td><td>4.81E-05</td><td>1.37E-03</td><td>39</td><td>1655</td><td>270</td><td>22304</td><td>multicellular organismal development</td><td>AT1G44900|AT5G14380|AT1G59640|AT3G23670|AT4G31820|AT4G18390|AT3G59420|AT1G70510|AT2G35340|AT4G20910|AT5G13300|AT1G02205|AT1G02800|AT1G23380|AT5G28640|AT3G10570|AT1G78770|AT5G58230|AT2G27990|AT1G61110|AT1G11130|AT5G23940|AT3G49670|AT2G13680|AT5G60690|AT4G21750|AT1G19850|AT1G30330|AT4G23750|AT5G09790|AT1G48410|AT1G18450|AT2G26330|AT5G37020|AT3G52940|AT3G63290|AT5G13290|AT2G28550|AT4G37750</td></tr>
<tr><td>48856</td><td>7.75E-05</td><td>2.13E-03</td><td>34</td><td>1392</td><td>270</td><td>22304</td><td>anatomical structure development</td><td>AT1G44900|AT5G14380|AT1G59640|AT4G31820|AT4G18390|AT3G59420|AT1G70510|AT2G35340|AT4G20910|AT5G13300|AT1G02205|AT1G23380|AT5G28640|AT3G10570|AT1G78770|AT5G58230|AT2G27990|AT1G11130|AT5G23940|AT3G49670|AT2G13680|AT5G60690|AT4G21750|AT1G19850|AT1G30330|AT4G23750|AT1G48410|AT1G18450|AT2G26330|AT5G37020|AT3G52940|AT5G13290|AT2G28550|AT4G37750</td></tr>
<tr><td>6996</td><td>8.11E-05</td><td>2.14E-03</td><td>18</td><td>526</td><td>270</td><td>22304</td><td>organelle organization</td><td>AT5G67270|AT3G54670|AT5G43990|AT3G06030|AT3G23670|AT1G04050|AT3G25500|AT3G20670|AT1G57820|AT4G05190|AT5G57320|AT1G76540|AT1G74850|AT1G18450|AT1G03780|AT1G51060|AT5G58230|AT1G06950</td></tr>
<tr><td>16310</td><td>1.25E-04</td><td>3.18E-03</td><td>25</td><td>910</td><td>270</td><td>22304</td><td>phosphorylation</td><td>AT5G43020|AT1G62950|AT3G24660|AT2G36350|AT1G55200|AT1G27190|AT1G49730|AT5G51560|AT3G51740|AT2G44830|AT3G57830|AT3G49670|AT1G09450|AT5G14210|AT5G67200|AT5G18910|AT2G26730|AT1G76540|AT4G36180|AT3G10540|AT5G51350|AT2G45590|AT1G60800|AT2G24230|AT4G34440</td></tr>
<tr><td>50793</td><td>1.33E-04</td><td>3.27E-03</td><td>12</td><td>273</td><td>270</td><td>22304</td><td>regulation of developmental process</td><td>AT1G11130|AT1G44900|AT4G20910|AT3G49670|AT1G76540|AT4G31820|AT4G18390|AT1G27360|AT2G13680|AT5G58230|AT5G13290|AT2G27990</td></tr>
<tr><td>7010</td><td>1.79E-04</td><td>4.26E-03</td><td>7</td><td>97</td><td>270</td><td>22304</td><td>cytoskeleton organization</td><td>AT4G05190|AT5G67270|AT5G57320|AT3G06030|AT3G23670|AT3G25500|AT1G03780</td></tr>
<tr><td>3006</td><td>2.04E-04</td><td>4.63E-03</td><td>23</td><td>829</td><td>270</td><td>22304</td><td>reproductive developmental process</td><td>AT1G11130|AT5G23940|AT3G49670|AT5G14380|AT1G59640|AT4G31820|AT3G59420|AT2G13680|AT1G70510|AT4G21750|AT1G19850|AT1G30330|AT4G23750|AT2G35340|AT4G20910|AT1G48410|AT1G18450|AT5G37020|AT3G10570|AT3G52940|AT5G58230|AT2G27990|AT2G28550</td></tr>
<tr><td>19219</td><td>2.08E-04</td><td>4.63E-03</td><td>35</td><td>1527</td><td>270</td><td>22304</td><td>regulation of nucleobase, nucleoside, nucleotide and nucleic acid metabolic process</td><td>AT1G35490|AT1G59640|AT4G18390|AT1G63100|AT1G31150|AT5G65410|AT1G57820|AT4G00480|AT1G49480|AT4G31805|AT5G08550|AT1G74850|AT5G66940|AT3G06160|AT4G35700|AT1G75240|AT1G78770|AT1G61110|AT2G35310|AT1G60860|AT4G31610|AT1G27360|AT5G60690|AT4G21750|AT1G30330|AT5G66770|AT4G23750|AT3G61250|AT1G16070|AT5G09790|AT5G37020|AT5G05610|AT4G14770|AT2G28550|AT4G37750</td></tr>
<tr><td>6464</td><td>2.51E-04</td><td>5.17E-03</td><td>30</td><td>1241</td><td>270</td><td>22304</td><td>protein modification process</td><td>AT5G43020|AT1G62950|AT3G24660|AT2G36350|AT1G55200|AT1G57820|AT1G27190|AT1G49730|AT1G27120|AT5G51560|AT3G51740|AT2G44830|AT3G57830|AT3G49670|AT4G26840|AT1G09450|AT2G32765|AT5G14210|AT5G67200|AT5G18910|AT1G11730|AT2G26730|AT1G76540|AT4G36180|AT3G10540|AT5G51350|AT2G45590|AT1G60800|AT2G24230|AT4G34440</td></tr>
<tr><td>42127</td><td>2.55E-04</td><td>5.17E-03</td><td>4</td><td>26</td><td>270</td><td>22304</td><td>regulation of cell proliferation</td><td>AT1G44900|AT4G34160|AT3G50070|AT4G37750</td></tr>
<tr><td>51171</td><td>2.59E-04</td><td>5.17E-03</td><td>35</td><td>1545</td><td>270</td><td>22304</td><td>regulation of nitrogen compound metabolic process</td><td>AT1G35490|AT1G59640|AT4G18390|AT1G63100|AT1G31150|AT5G65410|AT1G57820|AT4G00480|AT1G49480|AT4G31805|AT5G08550|AT1G74850|AT5G66940|AT3G06160|AT4G35700|AT1G75240|AT1G78770|AT1G61110|AT2G35310|AT1G60860|AT4G31610|AT1G27360|AT5G60690|AT4G21750|AT1G30330|AT5G66770|AT4G23750|AT3G61250|AT1G16070|AT5G09790|AT5G37020|AT5G05610|AT4G14770|AT2G28550|AT4G37750</td></tr>
</table>

<table>
<tr><td>48608</td><td>2.61E-04</td><td>5.17E-03</td><td>21</td><td>735</td><td>270</td><td>22304</td><td>reproductive structure development</td><td>AT1G11130|AT5G23940|AT3G49670|AT1G59640|AT4G31820|AT3G59420|AT1G70510|AT4G21750|AT1G19850|AT1G30330|AT4G23750|AT2G35340|AT4G20910|AT1G48410|AT1G18450|AT5G37020|AT3G10570|AT3G52940|AT5G58230|AT2G27990|AT2G28550</td></tr>
<tr><td>22403</td><td>2.98E-04</td><td>5.74E-03</td><td>6</td><td>75</td><td>270</td><td>22304</td><td>cell cycle phase</td><td>AT4G05190|AT4G34160|AT3G63130|AT1G76540|AT1G70210|AT1G03780</td></tr>
<tr><td>10556</td><td>3.32E-04</td><td>6.22E-03</td><td>34</td><td>1504</td><td>270</td><td>22304</td><td>regulation of macromolecule biosynthetic process</td><td>AT1G35490|AT1G59640|AT4G18390|AT1G63100|AT1G31150|AT5G65410|AT1G57820|AT4G00480|AT1G49480|AT4G31805|AT5G08550|AT1G74850|AT5G66940|AT3G06160|AT4G35700|AT1G75240|AT1G78770|AT1G61110|AT2G35310|AT4G31610|AT1G27360|AT5G60690|AT4G21750|AT1G30330|AT5G66770|AT4G23750|AT3G61250|AT1G16070|AT5G09790|AT5G37020|AT5G05610|AT4G14770|AT2G28550|AT4G37750</td></tr>
<tr><td>32502</td><td>3.47E-04</td><td>6.34E-03</td><td>39</td><td>1820</td><td>270</td><td>22304</td><td>developmental process</td><td>AT1G44900|AT5G14380|AT1G59640|AT3G23670|AT4G31820|AT4G18390|AT3G59420|AT1G70510|AT2G35340|AT4G20910|AT5G13300|AT1G02205|AT1G02800|AT1G23380|AT5G28640|AT3G10570|AT1G78770|AT5G58230|AT2G27990|AT1G61110|AT1G11130|AT5G23940|AT3G49670|AT2G13680|AT5G60690|AT4G21750|AT1G19850|AT1G30330|AT4G23750|AT5G09790|AT1G48410|AT1G18450|AT2G26330|AT5G37020|AT3G52940|AT3G63290|AT5G13290|AT2G28550|AT4G37750</td></tr>
<tr><td>22621</td><td>3.57E-04</td><td>6.36E-03</td><td>12</td><td>304</td><td>270</td><td>22304</td><td>shoot system development</td><td>AT1G11130|AT5G23940|AT5G13300|AT1G48410|AT4G31820|AT4G18390|AT5G28640|AT2G26330|AT4G21750|AT4G23750|AT5G58230|AT2G27990</td></tr>
<tr><td>43687</td><td>3.70E-04</td><td>6.36E-03</td><td>27</td><td>1093</td><td>270</td><td>22304</td><td>post-translational protein modification</td><td>AT5G43020|AT1G62950|AT3G24660|AT2G36350|AT1G55200|AT1G57820|AT1G27190|AT1G49730|AT5G51560|AT3G51740|AT2G44830|AT3G57830|AT3G49670|AT4G26840|AT1G09450|AT5G14210|AT5G67200|AT5G18910|AT2G26730|AT1G76540|AT4G36180|AT3G10540|AT5G51350|AT2G45590|AT1G60800|AT2G24230|AT4G34440</td></tr>
<tr><td>6796</td><td>3.78E-04</td><td>6.36E-03</td><td>25</td><td>979</td><td>270</td><td>22304</td><td>phosphate metabolic process</td><td>AT5G43020|AT1G62950|AT3G24660|AT2G36350|AT1G55200|AT1G27190|AT1G49730|AT5G51560|AT3G51740|AT2G44830|AT3G57830|AT3G49670|AT1G09450|AT5G14210|AT5G67200|AT5G18910|AT2G26730|AT1G76540|AT4G36180|AT3G10540|AT5G51350|AT2G45590|AT1G60800|AT2G24230|AT4G34440</td></tr>
<tr><td>6793</td><td>3.83E-04</td><td>6.36E-03</td><td>25</td><td>980</td><td>270</td><td>22304</td><td>phosphorus metabolic process</td><td>AT5G43020|AT1G62950|AT3G24660|AT2G36350|AT1G55200|AT1G27190|AT1G49730|AT5G51560|AT3G51740|AT2G44830|AT3G57830|AT3G49670|AT1G09450|AT5G14210|AT5G67200|AT5G18910|AT2G26730|AT1G76540|AT4G36180|AT3G10540|AT5G51350|AT2G45590|AT1G60800|AT2G24230|AT4G34440</td></tr>
<tr><td>48827</td><td>3.95E-04</td><td>6.40E-03</td><td>10</td><td>222</td><td>270</td><td>22304</td><td>phyllome development</td><td>AT5G23940|AT5G13300|AT1G48410|AT4G31820|AT4G18390|AT5G28640|AT2G26330|AT4G21750|AT4G23750|AT5G58230</td></tr>
<tr><td>51225</td><td>4.35E-04</td><td>6.57E-03</td><td>2</td><td>3</td><td>270</td><td>22304</td><td>spindle assembly</td><td>AT4G05190|AT1G03780</td></tr>
<tr><td>45449</td><td>4.50E-04</td><td>6.57E-03</td><td>33</td><td>1468</td><td>270</td><td>22304</td><td>regulation of transcription</td><td>AT1G35490|AT1G59640|AT4G18390|AT1G63100|AT1G31150|AT5G65410|AT1G57820|AT4G00480|AT1G49480|AT4G31805|AT5G08550|AT1G74850|AT5G66940|AT3G06160|AT4G35700|AT1G75240|AT1G61110|AT2G35310|AT4G31610|AT1G27360|AT5G60690|AT4G21750|AT1G30330|AT5G66770|AT4G23750|AT3G61250|AT1G16070|AT5G09790|AT5G37020|AT5G05610|AT4G14770|AT2G28550|AT4G37750</td></tr>
<tr><td>51325</td><td>4.59E-04</td><td>6.57E-03</td><td>3</td><td>13</td><td>270</td><td>22304</td><td>interphase</td><td>AT4G34160|AT1G76540|AT1G70210</td></tr>
<tr><td>51329</td><td>4.59E-04</td><td>6.57E-03</td><td>3</td><td>13</td><td>270</td><td>22304</td><td>interphase of mitotic cell cycle</td><td>AT4G34160|AT1G76540|AT1G70210</td></tr>
<tr><td>16043</td><td>4.60E-04</td><td>6.57E-03</td><td>24</td><td>935</td><td>270</td><td>22304</td><td>cellular component organization</td><td>AT1G11130|AT5G67270|AT5G23940|AT5G14380|AT1G08560|AT3G54670|AT5G43990|AT3G06030|AT3G23670|AT1G04050|AT3G25500|AT2G13680|AT3G20670|AT1G57820|AT4G05190|AT5G57320|AT1G76540|AT1G74850|AT1G18450|AT1G03780|AT1G51060|AT5G58230|AT2G41550|AT1G06950</td></tr>
<tr><td>7389</td><td>4.82E-04</td><td>6.57E-03</td><td>7</td><td>114</td><td>270</td><td>22304</td><td>pattern specification process</td><td>AT4G20910|AT5G13300|AT1G02800|AT5G60690|AT1G70510|AT1G19850|AT2G27990</td></tr>
<tr><td>9908</td><td>4.87E-04</td><td>6.57E-03</td><td>10</td><td>228</td><td>270</td><td>22304</td><td>flower development</td><td>AT1G11130|AT4G20910|AT3G49670|AT1G59640|AT4G31820|AT5G37020|AT3G10570|AT1G70510|AT1G19850|AT1G30330</td></tr>
</table>

31323	5.01E-04	6.57E-03	36	1664	270	22304	regulation of cellular metabolic process	AT1G35490|AT1G59640|AT4G18390|AT1G63100|AT1G31150|AT5G65410|AT1G57820|AT4G00480|AT1G49480|AT4G31805|AT5G08550|AT1G74850|AT5G66940|AT3G06160|AT4G35700|AT1G75240|AT1G78770|AT1G61110|AT2G35310|AT1G60860|AT4G31610|AT1G27360|AT5G60690|AT4G21750|AT1G30330|AT5G66770|AT4G23750|AT3G61250|AT1G16070|AT5G09790|AT5G37020|AT5G05610|AT4G14770|AT2G28550|AT4G37750|AT5G57690
51239	5.04E-04	6.57E-03	10	229	270	22304	regulation of multicellular organismal process	AT1G11130|AT1G44900|AT4G20910|AT3G49670|AT1G76540|AT4G31820|AT1G27360|AT2G13680|AT5G58230|AT5G13290
31326	5.07E-04	6.57E-03	34	1540	270	22304	regulation of cellular biosynthetic process	AT1G35490|AT1G59640|AT4G18390|AT1G63100|AT1G31150|AT5G65410|AT1G57820|AT4G00480|AT1G49480|AT4G31805|AT5G08550|AT1G74850|AT5G66940|AT3G06160|AT4G35700|AT1G75240|AT1G78770|AT1G61110|AT2G35310|AT4G31610|AT1G27360|AT5G60690|AT4G21750|AT1G30330|AT5G66770|AT4G23750|AT3G61250|AT1G16070|AT5G09790|AT5G37020|AT5G05610|AT4G14770|AT2G28550|AT4G37750
9889	5.07E-04	6.57E-03	34	1540	270	22304	regulation of biosynthetic process	AT1G35490|AT1G59640|AT4G18390|AT1G63100|AT1G31150|AT5G65410|AT1G57820|AT4G00480|AT1G49480|AT4G31805|AT5G08550|AT1G74850|AT5G66940|AT3G06160|AT4G35700|AT1G75240|AT1G78770|AT1G61110|AT2G35310|AT4G31610|AT1G27360|AT5G60690|AT4G21750|AT1G30330|AT5G66770|AT4G23750|AT3G61250|AT1G16070|AT5G09790|AT5G37020|AT5G05610|AT4G14770|AT2G28550|AT4G37750
80090	5.16E-04	6.57E-03	35	1604	270	22304	regulation of primary metabolic process	AT1G35490|AT1G59640|AT4G18390|AT1G63100|AT1G31150|AT5G65410|AT1G57820|AT4G00480|AT1G49480|AT4G31805|AT5G08550|AT1G74850|AT5G66940|AT3G06160|AT4G35700|AT1G75240|AT1G78770|AT1G61110|AT2G35310|AT1G60860|AT4G31610|AT1G27360|AT5G60690|AT4G21750|AT1G30330|AT5G66770|AT4G23750|AT3G61250|AT1G16070|AT5G09790|AT5G37020|AT5G05610|AT4G14770|AT2G28550|AT4G37750
10073	5.78E-04	7.23E-03	5	56	270	22304	meristem maintenance	AT1G44900|AT3G49670|AT1G23380|AT5G13290|AT2G27990
71103	6.62E-04	8.06E-03	6	87	270	22304	DNA conformation change	AT1G44900|AT3G20670|AT1G57820|AT1G51060|AT2G16440|AT5G58230
43412	6.67E-04	8.06E-03	31	1378	270	22304	macromolecule modification	AT5G43020|AT1G62950|AT3G24660|AT2G36350|AT1G55200|AT1G57820|AT1G27190|AT1G49730|AT1G27120|AT5G51560|AT3G51740|AT2G44830|AT3G57830|AT3G49250|AT3G49670|AT4G26840|AT1G09450|AT2G32765|AT5G14210|AT5G67200|AT5G18910|AT1G11730|AT2G26730|AT1G76540|AT4G36180|AT3G10540|AT5G51350|AT2G45590|AT1G60800|AT2G24230|AT4G34440
22603	7.32E-04	8.70E-03	4	34	270	22304	regulation of anatomical structure morphogenesis	AT1G11130|AT3G49670|AT1G76540|AT2G13680
6325	7.61E-04	8.90E-03	8	160	270	22304	chromatin organization	AT1G76540|AT5G43990|AT1G18450|AT1G04050|AT3G20670|AT1G57820|AT1G51060|AT5G58230
48509	8.57E-04	9.17E-03	5	61	270	22304	regulation of meristem development	AT1G44900|AT3G49670|AT1G76540|AT1G27360|AT5G13290
80	8.62E-04	9.17E-03	2	4	270	22304	G1 phase of mitotic cell cycle	AT4G34160|AT1G70210
51318	8.62E-04	9.17E-03	2	4	270	22304	G1 phase	AT4G34160|AT1G70210
70828	8.62E-04	9.17E-03	2	4	270	22304	heterochromatin organization	AT1G57820|AT5G58230
31507	8.62E-04	9.17E-03	2	4	270	22304	heterochromatin formation	AT1G57820|AT5G58230
7051	8.62E-04	9.17E-03	2	4	270	22304	spindle organization	AT4G05190|AT1G03780
48638	9.12E-04	9.56E-03	4	36	270	22304	regulation of developmental growth	AT1G44900|AT3G49670|AT2G13680|AT5G13290
19953	9.93E-04	1.03E-02	5	63	270	22304	sexual reproduction	AT5G07560|AT1G01370|AT1G18450|AT1G19890|AT4G37750
51276	1.09E-03	1.11E-02	9	210	270	22304	chromosome organization	AT3G54670|AT1G76540|AT5G43990|AT1G18450|AT1G04050|AT3G20670|AT1G57820|AT1G51060|AT5G58230
48367	1.14E-03	1.14E-02	11	300	270	22304	shoot development	AT5G23940|AT5G13300|AT1G48410|AT4G31820|AT4G18390|AT5G28640|AT2G26330|AT4G21750|AT4G23750|AT5G58230|AT2G27990

9791	1.18E-03	1.17E-02	22	884	270	22304	post-embryonic development	AT1G11130IAT5G23940IAT3G49670IAT1G59640IAT4G31820IAT3G59420IAT1G70510IAT4G21750IAT1G19850IAT1G30330IAT4G23750IAT2G35340IAT4G20910IAT1G48410IAT1G18450IAT2G26330IAT5G37020IAT3G10570IAT3G52940IAT5G58230IAT2G27990IAT2G28550
70925	1.43E-03	1.39E-02	2	5	270	22304	organelle assembly	AT4G05190IAT1G03780
7017	1.44E-03	1.39E-02	6	101	270	22304	microtubule-based process	AT1G12430IAT4G05190IAT3G63130IAT3G06030IAT1G18550IAT1G03780
6261	1.95E-03	1.85E-02	4	44	270	22304	DNA-dependent DNA replication	AT5G08550IAT1G44900IAT5G41880IAT2G16440
10496	2.12E-03	1.94E-02	2	6	270	22304	intercellular transport	AT2G33330IAT1G04520
10497	2.12E-03	1.94E-02	2	6	270	22304	plasmodesmata-mediated intercellular transport	AT2G33330IAT1G04520
10082	2.12E-03	1.94E-02	2	6	270	22304	regulation of root meristem growth	AT1G44900IAT5G13290
6355	2.15E-03	1.94E-02	20	810	270	22304	regulation of transcription, DNA-dependent	AT2G35310IAT1G35490IAT4G31610IAT1G31150IAT5G65410IAT5G60690IAT1G57820IAT4G21750IAT1G30330IAT4G23750IAT1G49480IAT4G31805IAT3G61250IAT5G09790IAT1G74850IAT3G06160IAT5G37020IAT5G05610IAT2G28550IAT4G37750
51252	2.24E-03	2.00E-02	20	813	270	22304	regulation of RNA metabolic process	AT2G35310IAT1G35490IAT4G31610IAT1G31150IAT5G65410IAT5G60690IAT1G57820IAT4G21750IAT1G30330IAT4G23750IAT1G49480IAT4G31805IAT3G61250IAT5G09790IAT1G74850IAT3G06160IAT5G37020IAT5G05610IAT2G28550IAT4G37750
10075	2.60E-03	2.29E-02	3	23	270	22304	regulation of meristem growth	AT1G44900IAT3G49670IAT5G13290
52249	2.95E-03	2.50E-02	2	7	270	22304	modulation of RNA levels in other organism involved in symbiotic interaction	AT4G20910IAT1G48410
9616	2.95E-03	2.50E-02	2	7	270	22304	virus induced gene silencing	AT4G20910IAT1G48410
52018	2.95E-03	2.50E-02	2	7	270	22304	modulation by symbiont of RNA levels in host	AT4G20910IAT1G48410
9965	3.04E-03	2.55E-02	6	117	270	22304	leaf morphogenesis	AT5G23940IAT5G13300IAT1G48410IAT4G18390IAT2G26330IAT5G58230
10016	3.08E-03	2.55E-02	7	157	270	22304	shoot morphogenesis	AT5G23940IAT5G13300IAT1G48410IAT4G18390IAT2G26330IAT5G58230IAT2G27990
9886	3.37E-03	2.76E-02	5	83	270	22304	post-embryonic morphogenesis	AT4G20910IAT1G59640IAT2G26330IAT3G59420IAT1G70510
30154	3.41E-03	2.76E-02	10	296	270	22304	cell differentiation	AT5G23940IAT3G49670IAT5G14380IAT4G18390IAT1G18450IAT2G13680IAT5G60690IAT4G21750IAT5G58230IAT2G27990
10564	3.71E-03	2.97E-02	3	26	270	22304	regulation of cell cycle process	AT1G76540IAT1G78770IAT2G33560
51817	3.90E-03	3.01E-02	2	8	270	22304	modification of morphology or physiology of other organism involved in symbiotic interaction	AT4G20910IAT1G48410
10143	3.90E-03	3.01E-02	2	8	270	22304	cutin biosynthetic process	AT5G23940IAT3G10570
44003	3.90E-03	3.01E-02	2	8	270	22304	modification by symbiont of host morphology or physiology	AT4G20910IAT1G48410
16568	3.93E-03	3.01E-02	5	86	270	22304	chromatin modification	AT1G76540IAT5G43990IAT1G04050IAT1G57820IAT5G58230
40008	4.41E-03	3.35E-02	4	55	270	22304	regulation of growth	AT1G44900IAT3G49670IAT2G13680IAT5G13290
31497	5.02E-03	3.70E-02	4	57	270	22304	chromatin assembly	AT3G20670IAT1G57820IAT1G51060IAT5G58230
48444	5.08E-03	3.70E-02	3	29	270	22304	floral organ morphogenesis	AT4G20910IAT1G59640IAT1G70510
48563	5.08E-03	3.70E-02	3	29	270	22304	post-embryonic organ morphogenesis	AT4G20910IAT1G59640IAT1G70510
48825	5.08E-03	3.70E-02	3	29	270	22304	cotyledon development	AT4G31820IAT4G21750IAT4G23750
3002	5.48E-03	3.95E-02	5	93	270	22304	regionalization	AT4G20910IAT5G13300IAT5G60690IAT1G70510IAT1G19850

<table>
<tr><td>9793</td><td>5.90E-03</td><td>4.18E-02</td><td>11</td><td>372</td><td>270</td><td>22304</td><td>embryonic development ending in seed dormancy</td><td>AT1G11130|AT5G23940|AT1G48410|AT4G31820|AT3G59420|AT3G52940|AT4G21750|AT1G19850|AT4G23750|AT5G58230|AT2G35340</td></tr>
<tr><td>6323</td><td>6.02E-03</td><td>4.18E-02</td><td>4</td><td>60</td><td>270</td><td>22304</td><td>DNA packaging</td><td>AT3G20670|AT1G57820|AT1G51060|AT5G58230</td></tr>
<tr><td>6268</td><td>6.16E-03</td><td>4.18E-02</td><td>2</td><td>10</td><td>270</td><td>22304</td><td>DNA unwinding involved in replication</td><td>AT1G44900|AT2G16440</td></tr>
<tr><td>10092</td><td>6.16E-03</td><td>4.18E-02</td><td>2</td><td>10</td><td>270</td><td>22304</td><td>specification of organ identity</td><td>AT4G20910|AT1G70510</td></tr>
<tr><td>10093</td><td>6.16E-03</td><td>4.18E-02</td><td>2</td><td>10</td><td>270</td><td>22304</td><td>specification of floral organ identity</td><td>AT4G20910|AT1G70510</td></tr>
<tr><td>10229</td><td>6.16E-03</td><td>4.18E-02</td><td>2</td><td>10</td><td>270</td><td>22304</td><td>inflorescence development</td><td>AT4G31820|AT2G27990</td></tr>
<tr><td>9790</td><td>6.34E-03</td><td>4.26E-02</td><td>12</td><td>429</td><td>270</td><td>22304</td><td>embryonic development</td><td>AT1G11130|AT1G44900|AT5G23940|AT1G48410|AT4G31820|AT3G59420|AT3G52940|AT4G21750|AT1G19850|AT4G23750|AT5G58230|AT2G35340</td></tr>
<tr><td>278</td><td>6.71E-03</td><td>4.47E-02</td><td>3</td><td>32</td><td>270</td><td>22304</td><td>mitotic cell cycle</td><td>AT4G34160|AT1G76540|AT1G70210</td></tr>
<tr><td>23052</td><td>6.79E-03</td><td>4.48E-02</td><td>22</td><td>1023</td><td>270</td><td>22304</td><td>signaling</td><td>AT1G11130|AT3G57830|AT3G49670|AT5G23400|AT5G43020|AT1G62950|AT3G25165|AT3G24660|AT3G25670|AT1G70895|AT5G14210|AT1G70510|AT5G46570|AT1G27190|AT2G33560|AT1G72260|AT1G76540|AT3G63300|AT4G36180|AT3G51740|AT5G51350|AT5G57690</td></tr>
<tr><td>9987</td><td>7.33E-03</td><td>4.80E-02</td><td>109</td><td>7393</td><td>270</td><td>22304</td><td>cellular process</td><td>AT1G44900|AT5G67270|AT1G13560|AT3G23670|AT1G04050|AT1G57820|AT1G70510|AT2G16640|AT5G46570|AT5G13000|AT3G51740|AT2G44830|AT5G44560|AT4G21070|AT3G49670|AT3G25165|AT1G09450|AT5G43990|AT1G16680|AT5G14210|AT3G20670|AT4G21750|AT1G72260|AT1G48410|AT2G24490|AT2G32670|AT3G54670|AT3G06030|AT3G24660|AT4G18390|AT2G36350|AT1G18550|AT5G08550|AT1G02205|AT1G49730|AT1G74850|AT5G58050|AT1G78770|AT1G11130|AT3G57830|AT3G49250|AT5G16690|AT5G23940|AT4G04930|AT3G25500|AT5G67200|AT5G18910|AT4G05190|AT4G34160|AT2G26730|AT1G18450|AT1G52570|AT3G10140|AT4G36180|AT5G37300|AT3G10540|AT1G80850|AT2G45590|AT2G41550|AT2G42120|AT4G34440|AT1G12430|AT5G14380|AT5G43020|AT5G41880|AT1G55200|AT2G16440|AT2G06925|AT1G27120|AT5G51560|AT4G34730|AT5G58230|AT5G16290|AT1G80070|AT4G26840|AT1G08560|AT2G13680|AT3G63130|AT1G76540|AT2G36190|AT5G42620|AT1G03780|AT1G51060|AT1G60800|AT4G12130|AT1G62950|AT1G30520|AT1G27190|AT4G24790|AT4G20910|AT5G57320|AT1G79820|AT1G70210|AT3G10570|AT4G31360|AT2G27990|AT2G32765|AT5G60690|AT4G23750|AT4G02150|AT1G11730|AT2G47610|AT5G63960|AT3G42670|AT1G08730|AT3G20260|AT5G51350|AT2G24230|AT1G06950</td></tr>
<tr><td>6270</td><td>7.47E-03</td><td>4.84E-02</td><td>2</td><td>11</td><td>270</td><td>22304</td><td>DNA-dependent DNA replication initiation</td><td>AT1G44900|AT2G16440</td></tr>
</table>
